# Supplementary material for: Is fatigue a cue to obtain iron supplements in Odisha, India? A mixed methods investigation
Source: BMJ Open. 2020 Oct 20;10(10):e037471. doi: 10.1136/bmjopen-2020-037471 (PMC7577027; doi:10.1136/bmjopen-2020-037471)
Supplement: Supplementary data [file bmjopen-2020-037471supp001.pdf]

Supplement 1

PERCEPTUAL MAPPING AND CARD SORTING QUESTIONNAIRE

FOR RESEARCH TEAM MEMBER USE ONLY/ କେବଳ ଅନୁସନ୍ଧାନକାରୀ ଦଳର ସଦସ୍ୟଙ୍କ ବ୍ୟବହାର ନିମନ୍ତେ:		
BLOCK	Which Block is the respondent from/ଉତ୍ତରଦାତା କେଉଁ ବ୍ଲକରୁ?	....
VILLAGE	Which village is the respondent from/ଉତ୍ତରଦାତା କେଉଁ ଗ୍ରାମରୁ?	
PARTICIPATION	The respondent is participating in a...<br>ଉତ୍ତରଦାତାଜଣକ କେଉଁଥିରେ ଭାଗ ନେଉଛନ୍ତି	Focus group discussion.......................1<br>ଦଳଗତ ଆଲୋଚନା...1<br>Key informant interview........................2<br>ପ୍ରମୁଖ ସୂଚନାଦାତାଙ୍କ ସହିତ ସାକ୍ଷାତକାର ....2<br>Perceptual Mapping -------------------3<br>ଧାରଣାତ୍ମକ ମାନଚିତ୍ରଣ -------------------------3
CODE_PM	What is the code number of the Perceptual Mapping?<br>ଏହି ଧାରଣାତ୍ମକ ମାନଚିତ୍ରଣର କୋଡ କଣ?	Code number/ କୋଡ ନମ୍ବର୍ __________<br>PM1<br>PM2<br>PM3<br>PM4<br>PM5<br>PM6<br>PM7<br>PM8<br>PM9

Yilma H, *et al. BMJ Open* 2020; 10:e037471. doi: 10.1136/bmjopen-2020-037471

		PM10<br>PM11<br>PM12
PARTICIPANT_PM	Participant category of Perceptual Mapping ଧାରଣାତ୍ମକ ମାନଚିତ୍ରଣରେ ଅଂଶଗ୍ରହଣ କରିଥିବା ଉତ୍ତରଦାତାଙ୍କ ପ୍ରକାର	Males 18 – 42 years old --------------------1<br>ପୁରୁଷ (୧୮-୪୨) (ବିବାହିତ) ------------------------1<br>WRA 15 – 35 years old ---------------------2<br>ପ୍ରଜନନ ବୟସ (୧୫-୩୫) ମଧ୍ୟରେ ଥିବା ମହିଳା ------2<br>Mothers in law – any age -----------------3<br>ଶାଶୁ (ସ୍ୱାମୀଙ୍କ ମା) ---------------------------------3
CODE_NUMBER_PM	Participant code number of the Perceptual Mapping ଧାରଣାତ୍ମକ ମାନଚିତ୍ରଣରେ ଭାଗ ନେଇଥିବା ସଦସ୍ୟଙ୍କ କୋଡ଼	
PATRICIPATION_DATE	On what date did the respondent participate/କେଉଁ ତାରିଖରେ ଉତ୍ତରଦାତା ଅଂଶଗ୍ରହଣ କରିଥିଲେ?	DD/MM: ________________________<br>ତାରିଖ / ମାସ ______________________

Thank you for participating in this study. I want to remind you that there are no right or wrong answers to these questions. We just want to know what your opinions are. Before we ask for your opinions, please tell us a few things about yourself/ଆପଣ ଏହି ଅଧ୍ୟୟନରେ ଅଂଶଗ୍ରହଣ କରିଥିବାରୁ ଧନ୍ୟବାଦ । ମୁଁ ଆପଣଙ୍କୁ ମନେ ପକେଇଦେବାକୁ ଚାହେଁ ଯେ ଏହି ସବୁ ପ୍ରଶ୍ନର ଠିକ କିମ୍ବା ଭୁଲ ଉତ୍ତର ନାହିଁ । ଆମେ କେବଳ ଆପଣଙ୍କ ମତାମତ ଗୁଡିକ ଜାଣିବାକୁ ଚାହୁଁଛୁ, ଦୟାକରି ଆପଣଙ୍କ ବିଷୟରେ ଆମକୁ କିଛି କୁହନ୍ତୁ ।

Section/ବିଭାଗ 1

Demographic Questionnaire/ଜନସଂଖ୍ୟା ସମ୍ବନ୍ଧୀୟ ପ୍ରଶ୍ନାବଳୀ

1	How old are you?<br>ଆପଣଙ୍କର ବୟସ କେତେ?	Age: ________________ (Unknown = -99)<br>ବୟସ: ------------------------ (ଜାଣି ନାହାନ୍ତି = -୯୯)
2	What is your sex?	Female/ମହିଳା 1

	ଆପଣଙ୍କର ଲିଙ୍ଗ କଣ?	Male/ପୁରୁଷ....................................2<br>No response/କୌଣସି ଉତ୍ତର ନାହିଁ..._......3
3	What is the highest level of school you have attended?<br>ଆପଣଙ୍କର ସର୍ବୋଚ୍ଚ ଶିକ୍ଷାଗତ ଯୋଗ୍ୟତା କଣ?	None/କିଛିନାହିଁ - 0<br>Primary school (incomplete)/ପ୍ରାଥମିକ ବିଦ୍ୟାଳୟ (ଅସମ୍ପୂର୍ଣ) - 1<br>Primary school (complete) /ପ୍ରାଥମିକ ବିଦ୍ୟାଳୟ (ସମ୍ପୂର୍ଣ) - 2<br>Secondary school (incomplete) /ମାଧ୍ୟମିକ ବିଦ୍ୟାଳୟ (ଅସମ୍ପୂର୍ଣ) - 3<br>Secondary school (complete) /ମାଧ୍ୟମିକ ବିଦ୍ୟାଳୟ (ସମ୍ପୂର୍ଣ) - 4<br>High Secondary/ Senior Secondary school (incomplete) /ଉଚ୍ଚ ମାଧ୍ୟମିକ ବିଦ୍ୟାଳୟ (ଅସମ୍ପୂର୍ଣ) - 5<br>High Secondary/ Senior school (complete) /ଉଚ୍ଚ ମାଧ୍ୟମିକ ବିଦ୍ୟାଳୟ (ସମ୍ପୂର୍ଣ) - 6<br>Tertiary/College/University (incomplete)/ମହାବିଦ୍ୟାଳୟ/ବିଶ୍ୱବିଦ୍ୟାଳୟ (ଅସମ୍ପୂର୍ଣ) - 7<br>Tertiary/College/University (complete)/ମହାବିଦ୍ୟାଳୟ/ବିଶ୍ୱବିଦ୍ୟାଳୟ (ସମ୍ପୂର୍ଣ) - 8<br>Other (Specify)/ଅନ୍ୟାନ୍ୟ (ଦର୍ଶାନ୍ତୁ):- 88<br>No response/କୌଣସି ଉତ୍ତରନାହିଁ............-99
4	Are you currently married or living together with a partner as if married?<br>ଆପଣ ବିବାହିତ କି ବା ଜଣେ ସାଥିଙ୍କ ସହିତ ବିବାହିତ ଭଳି ମିଶିକରି ରୁହନ୍ତି କି ?	Yes, currently married/ହଁ, ବିବାହିତ...........1<br>Yes, living with a partner/ହଁ, ଜଣେ ସାଥୀଙ୍କ ସହିତ ରହୁଛି ....2<br>No/ନାହିଁ...3<br>No response/ କୌଣସି ଉତ୍ତର ନାହିଁ...........-99
4a	IF NO: Are you divorced, separated, widowed, or never married or in a union?<br>(Skip if married or living with a partner.)	Never married or in a union/କେବେ ବିବାହ କରିନାହାନ୍ତି କିମ୍ବା କୌଣସି ସାଥୀଙ୍କ ସହିତ ରଖୁନାହାଁନ୍ତ...1<br>Divorced/separated/ଛାଡପତ୍ର/ଅଲଗା ରହୁଛନ୍ତି...2<br>Widow/widower / ବିଧବା / ବିପତି.............3

	ଯଦି ନା : ଆପଣ କଣ ଛାଡପତ୍ର ନେଇଛନ୍ତି, ଅଲଗା ରହୁଛନ୍ତି, ବିଧବା ବା କେବେ ବିବାହ କରିନାହାନ୍ତି କିମ୍ବା କୌଣସି ସାଥୀଙ୍କ ସହିତ ନାହାନ୍ତି ?<br>(ପଚାରନ୍ତୁ ନାହିଁ, ଯଦି ବିବାହିତ କିମ୍ବା ସାଥି ସହିତ ରହୁଛନ୍ତି)	No response/ କୌଣସି ଉତ୍ତର ନାହିଁ.............-99
5	What is your religion?<br>ଆପଣଙ୍କର ଧର୍ମ କଣ ?	Hindu/ହିନ୍ଦୁ ....1<br>Muslim/ମୁସଲିମ ....2<br>Christian/ଖ୍ରୀଷ୍ଟିୟାନ.............................3<br>Sikh/ଶିଖ.......................................4<br>Buddhist/ବୁଦ୍ଧ...................................5<br>Jain/ଜୈନ...6<br>Other/ଅନ୍ୟାନ୍ୟ..................................7<br>No response/ କୌଣସି ଉତ୍ତର ନାହିଁ.........-99
CASTE	What is your caste ?<br>ଆପଣଙ୍କର ଜାତି କଣ ?	Scheduled Caste ଅନୁସୂଚିତ ଜାତି - 1<br>Scheduled Tribe ଅନୁସୂଚିତ ଜନଜାତି - 2<br>OBC ଅନ୍ୟାନ୍ୟ ପଛୁଆ ବର୍ଗର ଜାତି - 3<br>Other Caste ଅନ୍ୟାନ୍ୟ ଜାତି - 4<br>No response/ କୌଣସି ଉତ୍ତର ନାହିଁ.........-99
6	How many children do you have?<br>ଆପଣଙ୍କର କେତୋଟି ସନ୍ତାନ ଅଛନ୍ତି ?	None/କିଛିନାହିଁ....................................0<br>One/ଗୋଟିଏ.......................................1<br>Two/ଦୁଇଟି...2<br>Three/ତିନୋଟି....................................3<br>Four/ଚାରିଟି......................................4<br>Five or more/ପାଞ୍ଚ କିମ୍ବା ଅଧିକ................5<br>No response// କୌଣସି ଉତ୍ତର ନାହିଁ...........-99
7	How often do you watch television?<br>ଆପଣ କେତେ ବ୍ୟବଧାନରେ ଟିଭି ଦେଖନ୍ତି ?	Not at all/କେବେ ନାହିଁ................................0<br>Less than once a week/ସପ୍ତାହରେ ଥରକରୁ କମ୍...1<br>At least once a week/ ଅତିକମ୍ ରେ ସପ୍ତାହକୁ ଥରେ 2

		Almost Every Day/ପ୍ରାୟ ପ୍ରତ୍ୟକ ଦିନ............3
7a	On an average day, for how long do you watch television?<br>ଗୋଟିଏ ଦିନରେ, ପାଖାପାଖି କେତେ ସମୟ ଟିଭି ଦେଖନ୍ତି ?	______Hours per day/ OR ____ minutes per day<br>ଦିନରେ .... ଘଣ୍ଟା କିମ୍ବା ଦିନରେ .... ମିନିଟ୍
8	How often do you listen to the radio?<br>ଆପଣ କେତେଥର ରେଡିଓ ଶୁଣନ୍ତି ?	Not at all/କେବେ ନାହିଁ...............................0<br>Less than once a week/ ସପ୍ତାହରେ ଥରକରୁ କମ୍...1<br>At least once a week/ ଅତିକମ୍ ରେ ସପ୍ତାହକୁ ଥରେ ....2<br>Almost Every Day/ ପ୍ରାୟ ସବୁଦିନ.................3
8a	On an average day, for how long do you listen to the radio?<br>ଗୋଟିଏ ଦିନରେ ପାଖାପାଖି କେତେ ସମୟ ରେଡିଓ ଶୁଣନ୍ତି?	______Hours per day OR ____ minutes per day<br>ଦିନରେ .... ଘଣ୍ଟା କିମ୍ବା ଦିନରେ .... ମିନିଟ
9.0	How often do you talk on a mobile phone?<br>ଆପଣ କେତେଥର ମୋବାଇଲରେ କଥା ହୁଅନ୍ତି?	Not at all/କେବେ ନାହିଁ...............................0<br>Less than once a week/ ସପ୍ତାହରେ ଥରକରୁ କମ୍...1<br>At least once a week/ ଅତିକମ୍ ରେ ସପ୍ତାହକୁ ଥରେ ....2<br>Almost Every Day/ପ୍ରାୟ ସବୁଦିନ.................3
9	Do you use a smart phone?<br>ଆପଣ ସ୍ମାର୍ଟ ଫୋନ୍ ବ୍ୟବହାର କରନ୍ତି କି ?	Yes/ହଁ...1<br>No/ନା..0
9a	On an average day, for how long do you talk on a mobile phone?<br>ଗୋଟିଏ ଦିନରେ, ପାଖାପାଖି କେତେ ସମୟ ମୋବାଇଲରେ କଥା ହୁଅନ୍ତି?	______Hours per day OR ____ minutes per day<br>ଦିନରେ .... ଘଣ୍ଟା କିମ୍ବା ଦିନରେ .... ମିନିଟ
10	How often do you read the newspaper?<br>ଆପଣ କେତେଥର ଖବରକାଗଜ ପଢନ୍ତି?	Not at all/କେବେ ନାହିଁ...............................0<br>Less than once a week/ ସପ୍ତାହରେ ଥରକରୁ କମ୍...1<br>At least once a week/ ଅତିକମ୍ ରେ ସପ୍ତାହକୁ ଥରେ ....2<br>Almost Every Day/ପ୍ରାୟ ସବୁଦିନ.................3

Yilma H, *et al. BMJ Open* 2020; 10:e037471. doi: 10.1136/bmjopen-2020-037471

10a	On an average day, for how long do you read the newspaper?<br>ଗୋଟିଏ ଦିନରେ, ପାଖାପାଖି କେତେ ସମୟ ଖବରକାଗଜ ପଢନ୍ତି?	______Hours per day OR ____ minutes per day<br>ଦିନରେ .... ଘଣ୍ଟା କିମ୍ବା ଦିନରେ .... ମିନିଟ
11	Have you ever attended a self-help group meeting?<br>ଆପଣ କେବେ ସ୍ୱୟଂ ସହାୟକ ଦଳର ବୈଠକରେ ଯୋଗ ଦେଇଛନ୍ତି କି ?	Yes/ହଁ..1<br>No/ନା..0
11a	Are you currently a part of a self-help group/ଆପଣ ବର୍ତ୍ତମାନ ସ୍ୱୟଂ ସହାୟକ ଦଳର ଜଣେ /ସଦସ୍ୟ କି?	Yes/ହଁ..1<br>No/ନା..0
12	Have you ever taken iron & folic acid supplements (IFA)/ଆପଣ କେବେ ବି ଆଇରନ ଏବଂ ଫଲିକ ଏସିଡର ପରିପୂରକ ଖାଇଛନ୍ତି କି ?	Yes/ହଁ..1<br>No/ନା..0
12a	Are you currently taking iron & folic acid supplements (IFA)/ଆପଣ ବର୍ତ୍ତମାନ ଆଇରନ ଏବଂ ଫଲିକ ଏସିଡର ପରିପୂରକ ଖାଉଛନ୍ତି କି ?	Yes/ହଁ..1<br>No/ନା..0
13	Has a doctor or healthcare provider ever told you that you have anemia/ଆପଣଙ୍କର ରକ୍ତହୀନତା ଅଛି ବୋଲି ଆପଣଙ୍କୁ ଡାକ୍ତର କିମ୍ବା କୌଣସି ସ୍ୱାସ୍ଥ୍ୟ ପ୍ରଦାନକାରୀ କେବେ କହିଛନ୍ତି କି ?	Yes/ହଁ..1<br>No/ନା..0
13a	Do you currently have anemia/ବର୍ତ୍ତମାନ ଆପଣଙ୍କର ରକ୍ତହୀନତା ରହିଛି କି ?	Yes/ହଁ..1<br>No/ନା..0

Yilma H, *et al. BMJ Open* 2020; 10:e037471. doi: 10.1136/bmjopen-2020-037471

Section/ବିଭାଗ 2

I am now going to show you two cards at a time. One card has the picture of one object and the second card has the picture of a second object. Please tell me how similar the second card is to the first. To make this a bit easier, I will show you the second card and first ask you whether it is similar or different. If you say "similar," then I will ask you whether it is somewhat similar or very similar; if you say "different," then I will ask you whether it is somewhat different or very different.

ଏବେ ମୁଁ ଆପଣଙ୍କୁ ଏକ ସମୟରେ ଦୁଇଟି କାର୍ଡ ଦେଖାଇବାକୁ ଯାଉଛି | ଗୋଟିଏ କାର୍ଡରେ ଗୋଟିଏ ବିଷୟରେ ଛବି ଏବଂ ଦ୍ୱିତୀୟ କାର୍ଡରେ ଦ୍ୱିତୀୟ ବିଷୟରେ ଛବି ରହିଛି । ଦୟାକରି ମୋତେ କୁହନ୍ତୁ ଦ୍ୱିତୀୟ କାର୍ଡଟି ପ୍ରଥମ କାର୍ଡ ସହିତ କେତେ ସମାନ ଅଛି । ଆମେ ଏହାକୁ ଆଉ ଟିକେ ସହଜ କରିବା ପାଇଁ ମୁଁ ଆପଣଙ୍କୁ ଦ୍ୱିତୀୟ କାର୍ଡଟି ଦେଖାଇବି ଏବଂ ପ୍ରଥମେ ପଚାରିବି ଏହା ସମାନ କିମ୍ବା ଅଲଗା । ଯଦି ଆପଣ ସମାନ କହନ୍ତି ତେବେ କିଛି ସମାନ ନା ସମ୍ପୂର୍ଣ ସମାନ ବୋଲି ମୁଁ ପଚାରିବି; ଯଦି ଆପଣ ଅଲଗା କହନ୍ତି ତେବେ କିଛି ଅଲଗା ନା ସମ୍ପୂର୍ଣ ଅଲଗା ବୋଲି ମୁଁ ପଚାରିବି ।

[Show respondent each card pair: 1 versus 2, 3, 4,..... 16; 2 versus 3, 4, 5, ... 16; etc.]

[ଉତ୍ତରଦାତାଙ୍କୁ ପ୍ରତ୍ୟେକ କାର୍ଡର ଯୋଡି ଦେଖାନ୍ତୁ: 1 ସହିତ 2, 3, 4 ... 16; 2 ସହିତ 3, 4, 5, ... 16 ଇତ୍ୟାଦି]

1. IFA Pill/ଆଇରନ ବଟିକା
2. Clinic/କ୍ଲିନିକ
3. Kiosk
4. Self-help group/ସ୍ୱୟଂ ସହାୟକ ଦଳ
5. Traditional healer/ ପାରମ୍ପରିକ / ଚିକିତ୍ସକ / ବୈଦ୍ୟ
6. Physician/ ଚିକିତ୍ସକ / ଡାକ୍ତର
7. Fatigue/କ୍ଳାନ୍ତି
8. Prenatal care/ ଗର୍ଭାବସ୍ଥା ସମୟରେ ଯତ୍ନ
9. Green vegetables/ସବୁଜ ପନିପରିବା
10. Meat/ମାଂସ
11. Pregnancy/ଗର୍ଭାବସ୍ଥା
12. Stomach ache/ପେଟ କାଟିବା

[For each pair, first ask: Is XXXX similar or different from YYYY/ପ୍ରତ୍ୟେକ ଯୋଡି ପାଇଁ ପ୍ରଥମେ ପଚାରନ୍ତୁ XXXX ସମାନ କିମ୍ବା YYYY ଠାରୁ ଅଲଗା ।

Yilma H, *et al. BMJ Open* 2020; 10:e037471. doi: 10.1136/bmjopen-2020-037471

If Respondent says, “similar,” then ask: Would you say just a bit similar or very similar?

ଯଦି ଉତ୍ତରଦାତା ସମାନ କହନ୍ତି ତେବେ ପଚାରନ୍ତୁ ଆପଣ କିଛି ସମାନ କହୁଛନ୍ତି କିମ୍ବା ସମ୍ପୂର୍ଣ ସମାନ କହୁଛନ୍ତି?

If Respondent says, “different,” then ask: Would you say just a bit different or very different?

ଯଦି ଉତ୍ତରଦାତା ଅଲଗା କହନ୍ତି ତେବେ ପଚାରନ୍ତୁ ଆପଣ କିଛି ଅଲଗା କହୁଛନ୍ତି କିମ୍ବା ସମ୍ପୂର୍ଣ ଅଲଗା କହୁଛନ୍ତି?]

Yilma H, *et al. BMJ Open* 2020; 10:e037471. doi: 10.1136/bmjopen-2020-037471

Card 1	Card 2	1 = very different/ସମ୍ପୂର୍ଣ ଅଲଗା<br>2 = somewhat differentକିଛି ଅଲଗା<br>3 = somewhat similarକିଛି ସମାନ<br>4 = very similarସମ୍ପୂର୍ଣ ସମାନ
1. IFA pill/ଆଇରନ ବଟିକା	2	
	3	
	4	
	5	
	6	
	7	
	8	
	9	
	10	
	11	
	12	
2. Clinic କ୍ଲିନିକ	3	
	4	
	5	
	6	
	7	
	8	
	9	
	10	
	11	
	12	

Card 1	Card 2	1 = very different/ ସମ୍ପୂର୍ଣ ଅଲଗା<br>2 = somewhat different କିଛି ଅଲଗା<br>3 = somewhat similar କିଛି ସମାନ<br>4 = very similar ସମ୍ପୂର୍ଣ ସମାନ
3. Kiosk	4	
	5	
	6	
	7	
	8	
	9	
	10	
	11	
	12	
4. SHG ସ୍ୱୟଂ ସହାୟକ ଦଳ	5	
	6	
	7	
	8	
	9	
	10	
	11	
	12	

Card 1	Card 2	1 = very different/ସମ୍ପୂର୍ଣ ଅଲଗା<br>2 = somewhat differentକିଛି ଅଲଗା<br>3 = somewhat similar କିଛି ସମାନ<br>4 = very similar ସମ୍ପୂର୍ଣ ସମାନ
5. Traditional healer ପାରମ୍ପାରିକ ଚିକିତ୍ସକ / ବୈଦ୍ୟ	6	
	7	
	8	
	9	
	10	
	11	
	12	
6. Physician ଡାକ୍ତର	7	
	8	
	9	
	10	
	11	
	12	
7. Fatigue କ୍ଲାନ୍ତି	8	
	9	
	10	
	11	
	12	

Yilma H, *et al. BMJ Open* 2020; 10:e037471. doi: 10.1136/bmjopen-2020-037471

Card 1	Card 2	1 = very different/ ସମ୍ପୂର୍ଣ୍ଣ ଅଲଗା<br>2 = somewhat different କିଛି ଅଲଗା<br>3 = somewhat similar କିଛି ସମାନ<br>4 = very similar ସମ୍ପୂର୍ଣ୍ଣ ସମାନ
8. Prenatal care ଗର୍ଭାବସ୍ଥା ସମୟରେ ଯତ୍ନ	9	
	10	
	11	
	12	
9. Green vegetables ସବୁଜ ପନିପରିବା	10	
	11	
	12	
10. Meat ମାଂସ	11	
	12	
11. Pregnancy/ଗର୍ଭାବସ୍ଥା	12	

Yilma H, *et al. BMJ Open* 2020; 10:e037471. doi: 10.1136/bmjopen-2020-037471

Section/ବିଭାଗ 3

Importance for Health and Well-Being/ସ୍ୱାସ୍ଥ୍ୟ ଏବଂ ଭଲ ରହିବା ପାଇଁ ମହତ୍ୱ

I will now show you the same 12 cards. Please put these cards in three different piles. In the first pile, put all the cards that you think are very important for your health and well-being. In the second pile, put all the cards that you think are just a little bit important for your health and well-being. In the third pile, put all the cards that you think are not important at all for your health and well-being. [Do the exercise and write response in the first table. Then shuffle the cards.]

ଏବେ ମୁଁ ଆପଣଙ୍କୁ ୧ ୨ଟି କାର୍ଡ ଦେଖାଇବି । ଏହି କାର୍ଡଗୁଡିକ ତିନୋଟି ଅଲଗା ଗଦାରେ ରଖନ୍ତୁ | ଆପଣ ଯେଉଁ ସବୁକାର୍ଡ ଗୁଡିକ ଭାବୁଛନ୍ତି ଆପଣଙ୍କର ସ୍ୱାସ୍ଥ୍ୟ ଏବଂ ଭଲରହିବା ପାଇଁ ଜରୁରୀ ସେଗୁଡିକ ପ୍ରଥମ ଗଦାରେ ରଖନ୍ତୁ | ଯେଉଁସବୁ କାର୍ଡଗୁଡିକ ଆପଣଙ୍କ ସ୍ୱାସ୍ଥ୍ୟ ଏବଂ ଭଲ ରହିବା ପାଇଁ କମ ଜରୁରୀ ସେଗୁଡିକ ଦ୍ୱିତୀୟ ଗଦାରେ ରଖନ୍ତୁ | ସେହିପରି, ଯେଉଁସବୁ କାର୍ଡଗୁଡିକ ଆପଣଙ୍କ ସ୍ୱାସ୍ଥ୍ୟ ଏବଂ ଭଲରହିବା ପାଇଁ ଜମା ଜରୁରୀ ନୁହେଁ, ସେଗୁଡିକ ତୃତୀୟ ଗଦାରେ ଅଛି ? (ଏହି ଖେଳଟିକୁ କରାନ୍ତୁ ଏବଂ ଉତ୍ତରକୁ ପ୍ରଥମ ଟେବୁଲରେ ଲେଖନ୍ତୁ | ତାହାପରେ କାର୍ଡଗୁଡିକ ମିଶାଇଦିଅନ୍ତୁ |)

Easy/ସହଜ

This time please think about how easy it is to get (or to use or to do) each item shown in the card. If you think the item is very easy to get (or to use or to do), put it in the first pile. If you think the item is sometimes easy and sometimes difficult to get (or to use or to do), put it in the second pile. If you think it is very hard to get (or to use or to do), put it in the third pile. [Write response in the second table. Then shuffle.]

ଦୟାକରି ଏଥର ଚିନ୍ତା କରନ୍ତୁ କାର୍ଡରେ ଦେଖା ଯାଇଥିବା ପ୍ରତ୍ୟେକ ଜିନିଷକୁ କେତେ ସହଜରେ ପାଇ ପାରିବେ । ଯଦି ଭାବୁଛନ୍ତି ଏହି ଜିନିଷଗୁଡ଼ିକ ବହୁତ ସହଜରେ ପାଇପାରିବେ (ବା ବ୍ୟବହାରରେ ଲଗାଇପାରିବେ ବା କାମରେ ଲଗାଇପାରିବେ) ସେଗୁଡିକ ପ୍ରଥମ ଗଦାରେ ରଖନ୍ତୁ | ଯଦି ଭାବୁଛନ୍ତି ଏହି ଜିନିଷଗୁଡ଼ିକ ପାଇବାକୁ ବେଳେବେଳେ ସହଜ ଏବଂ ବେଳେବେଳେ କଷ୍ଟ (ବା ବ୍ୟବହାରରେ ଲଗାଇପାରିବେ ବା କାମରେ ଲଗାଇପାରିବେ), ତାହାକୁ ତୃତୀୟ ଗଦାରେ ରଖନ୍ତୁ | ଯଦି ଭାବୁଛନ୍ତି, ଏହା ପାଇବାକୁ (ବା ବ୍ୟବହାରରେ ଲଗାଇପାରିବେ ବା କାମରେ ଲଗାଇପାରିବେ) ବହୁତ କଷ୍ଟସାଧ ତାହାକୁ ତୃତୀୟ ଗଦାରେ ରଖନ୍ତୁ | (ଉତ୍ତରଗୁଡିକ ଦ୍ୱିତୀୟ ଟେବୁଲ ରେ ଲେଖନ୍ତୁ ଏବଂ ସେଗୁଡିକ ପରେ ମିଶାଇଦିଅନ୍ତୁ |)

Like/ପସନ୍ଦ

Finally, we will now sort these into three piles according to how much you like each card. If you like it a lot, put it in the first pile. If you like it just a bit or dislike it just a bit, put it in the second pile. And, if you dislike it a lot, put it in the third pile. [Record in the third table] ଶେଷରେ, ଆପଣ ପ୍ରତ୍ୟକ କାର୍ଡକୁ କେତେ ପସନ୍ଦ କଲେ, ସେହି ଅନୁସାରେ ଆଗେ ଏହାକୁ ତିନୋଟି ଗଦାରେ ସଜାଇ ରଖିବା | ଯଦି ଆପଣ ଏହାକୁ ବହୁତ ପସନ୍ଦ କରନ୍ତି, ତାହେଲେ

Yilma H, *et al. BMJ Open* 2020; 10:e037471. doi: 10.1136/bmjopen-2020-037471

ତାକୁ ପ୍ରଥମ ଗଦାରେ ରଖନ୍ତୁ | ଯଦି ଆପଣ ଏହାକୁ କମ ପସନ୍ଦ କରନ୍ତି ବା କମ ପସନ୍ଦ କରନ୍ତି ନାହିଁ, ତାହାକୁ ଦ୍ୱିତୀୟ ଗଦାରେ ରଖନ୍ତୁ | ଯଦି ଆପଣ ସମ୍ପୃକ୍ତ ପସନ୍ଦ କରନ୍ତି ନାହିଁ, ତାହାକୁ ତୃତୀୟ ଗଦାରେ ରଖନ୍ତୁ) (ତୃତୀୟ ଟେବୁଲରେ ଲେଖନ୍ତୁ)

Write the card number in each column.

PILE 1:<br>Very important<br>ବହୁତ ଜରୁରୀ	PILE2:<br>Somewhat important<br>କିଛିମାତ୍ରାରେ ଜରୁରୀ	PILE 3:<br>Unimportant<br>ଜରୁରୀ ନୁହେଁ

PILE 1:<br>Very easy to get<br>ବହୁତ ସହଜରେ ପାଇ ହେବ	PILE 2:<br>Somewhat easy<br>କମ୍ ସହଜରେ ପାଇ ହେବ	PILE 3:<br>Very hard to get<br>ପାଇବାକୁ ବହୁତ କଷ୍ଟସାଧ୍ୟ

PILE 1:<br>Like a lot<br>ବହୁତ ପସନ୍ଦ କରନ୍ତି	PILE 2:<br>Like somewhat<br>କିଛି ପସନ୍ଦ କରନ୍ତି	PILE 3:<br>Dislike<br>ପସନ୍ଦ କରନ୍ତି ନାହିଁ

Yilma H, *et al. BMJ Open* 2020; 10:e037471. doi: 10.1136/bmjopen-2020-037471
