# Supplementary material for: Is fatigue a cue to obtain iron supplements in Odisha, India? A mixed methods investigation
Source: BMJ Open. 2020 Oct 20;10(10):e037471. doi: 10.1136/bmjopen-2020-037471 (PMC7577027; doi:10.1136/bmjopen-2020-037471)
Supplement: Author's manuscript [file bmjopen-2020-037471.draft_revisions.pdf]

BMJ Open

BMJ Open is committed to open peer review. As part of this commitment we make the peer review history of every article we publish publicly available.

When an article is published we post the peer reviewers' comments and the authors' responses online. We also post the versions of the paper that were used during peer review. These are the versions that the peer review comments apply to.

The versions of the paper that follow are the versions that were submitted during the peer review process. They are not the versions of record or the final published versions. They should not be cited or distributed as the published version of this manuscript.

BMJ Open is an open access journal and the full, final, typeset and author-corrected version of record of the manuscript is available on our site with no access controls, subscription charges or pay-per-view fees (http://bmjopen.bmj.com).

If you have any questions on BMJ Open's open peer review process please email info.bmjopen@bmj.com

BMJ Open

Anemia reduction in India: exploring fatigue as a cue to seek treatment

Journal:	*BMJ Open*
Manuscript ID	bmjopen-2020-037471
Article Type:	Original research
Date Submitted by the Author:	04-Feb-2020
Complete List of Authors:	Yilma, Hagere; Prevention and Community Health<br>Sedlander, Erica; George Washington University Milken Institute of Public Health, Prevention and Community Health<br>Rimal, Rajiv; Johns Hopkins Bloomberg School of Public Health Center for Teaching and Learning, Health, Behavior and Society<br>Pattnaik, Soumik; D-COR Consulting
Keywords:	PUBLIC HEALTH, QUALITATIVE RESEARCH, Nutrition < TROPICAL MEDICINE, Anaemia < HAEMATOLOGY

SCHOLARONE™
Manuscripts

I, the Submitting Author has the right to grant and does grant on behalf of all authors of the Work (as defined in the below author licence), an exclusive licence and/or a non-exclusive licence for contributions from authors who are: i) UK Crown employees; ii) where BMJ has agreed a CC-BY licence shall apply, and/or iii) in accordance with the terms applicable for US Federal Government officers or employees acting as part of their official duties; on a worldwide, perpetual, irrevocable, royalty-free basis to BMJ Publishing Group Ltd ("BMJ") its licensees and where the relevant Journal is co-owned by BMJ to the co-owners of the Journal, to publish the Work in this journal and any other BMJ products and to exploit all rights, as set out in our licence.

The Submitting Author accepts and understands that any supply made under these terms is made by BMJ to the Submitting Author unless you are acting as an employee on behalf of your employer or a postgraduate student of an affiliated institution which is paying any applicable article publishing charge ("APC") for Open Access articles. Where the Submitting Author wishes to make the Work available on an Open Access basis (and intends to pay the relevant APC), the terms of reuse of such Open Access shall be governed by a Creative Commons licence – details of these licences and which Creative Commons licence will apply to this Work are set out in our licence referred to above.

Other than as permitted in any relevant BMJ Author's Self Archiving Policies, I confirm this Work has not been accepted for publication elsewhere, is not being considered for publication elsewhere and does not duplicate material already published. I confirm all authors consent to publication of this Work and authorise the granting of this licence.

For peer review only

Title: Anemia reduction in India: exploring fatigue as a cue to seek treatment

Authors: Hagere Yilma, MPH[1], Erica Sedlander, DrPH (c) MPH[1], Rajiv N. Rimal, PhD[2], Soumik Pattnaik[3]

[1] George Washington University, Milken Institute School of Public Health, Department of Prevention and Community Health
[2] Johns Hopkins University Bloomberg School of Public Health, Health, Behavior, and Society
[3] DCOR Consulting

Hagere Yilma, MPH (corresponding author)
950 New Hampshire, Department of Prevention and Community Health
202.994.1486
https://orcid.org/0000-0002-4042-3484
hyilma@gwu.edu

Erica Sedlander, DrPH (c), MPH
950 New Hampshire, Department of Prevention and Community Health
202.994.0849
esedlander@gwu.edu

Rajiv N. Rimal, PhD
615 N Wolfe St, Department of Health, Behavior, and Society
rrimal1@jhu.edu

Soumik Pattnaik
H.B Colony, Punjabi Colony, Satya Nagar, Bhubaneswar, Odisha, India
+91 94384 63984
soumik.dcor@gmail.com

For peer review only

Abstract

Objectives: This study aims to understand if the main symptom of anemia, fatigue, is an adequate health concern that can cue women of reproductive age and their referent groups to obtain iron supplements.

Design: cross-sectional perceptual mapping and card sorting activity and 16 focus group discussions with women of reproductive age, mothers-in-law, and males.

Setting: Bhubaneswar, Odisha, India

Participants: Women of reproductive age (age 15-49; *n* =30), mothers-in-law (*n* = 30), and married men (*n* = 30) were randomly selected to participate in the perceptual mapping and card sorting exercise. A separate sample of each group was randomly selected for the focus group discussions (*n* = 148).

Primary and secondary outcome measures: We collected perceptions of dissimilarity between anemia-related items, including fatigue, iron supplements, and iron distribution channels, as well as the extent to which these items were perceived as important to health or likable. Focus group discussions collected information about gender roles and norms.

Results: Cognitive maps and card sorting revealed that fatigue was conceptualized distinctly from items related to iron supplement distribution channels and that perceptions around fatigue's importance to health were low among women and their referent groups. Women reported that fatigue is a part of their daily life; they also expressed that their family's well-being takes precedence over their own.

Conclusion: Our results indicate that fatigue is currently not an adequate cue to seek treatment, perhaps due to the normalization of fatigue as a part of women's daily life. Future anemia reduction interventions should shift perceptions of fatigue among women and their referent groups so that it is recognized as a symptom of illness. Additionally,

front-line workers should also distribute iron supplements to non-pregnant, out-of-school women.

Keywords: anemia; gender norms; iron folic acid; perceptual mapping; women's health

For peer review only

Strengths and Limitations of the study

- We used an innovative methodology known as perceptual mapping, coupled with a card sorting exercise and qualitative interviews to understand how women of reproductive age, and their relevant referent groups (i.e., mothers-in-law and males) perceive fatigue.
- Qualitative focus groups were used to minimize the ambiguity of the perceptual mapping plots.
- The size of each subsample was small (n=30 each), reducing our ability to find significant differences from the card sorting activity.

For peer review only

For peer review only - http://bmjopen.bmj.com/site/about/guidelines.xhtml

Background

Anemia is a condition characterized by a low red blood cell count that affects a quarter of all people worldwide (Benoist et al., 2008). If left untreated, anemia can lead to a number of poor health outcomes, including poor mental health (Strauss & Auerbach, 2018; Wouters et al., 2019), reduced work capacity (Gardner, Edgerton, Senewiratne, Barnard, & Ohira, 1977), pregnancy complications (Allen, 2000; Brabin, Hakimi, & Pelletier, 2001), and sometimes death (Wouters et al., 2019). Women of reproductive age are the most vulnerable for anemia (Benoist et al., 2008), as blood loss during menstruation and childbirth raises their risk (Beard, 2000; Cooke, McCavit, Buchanan, & Powers, 2017; Hallberg, Högdahl, Nilsson, & Rybo, 1966; Milman, 2011). Recent research suggests that social structure may exacerbate anemia prevalence in women, particularly as it relates to gender norms (Aurino, 2017; Bedwell, Jose, 2011; Spielvogel, Bellido, & Vitzthum, 2017).

The World Health Organization (WHO) has made anemia one of six global nutritional targets (World Health Organization, 2014). Among the primary recommendations for the treatment and prevention of anemia is iron and folic acid supplementation, an effective strategy in low- and middle- income countries (WHO, 2016; Ahmed et al., 2010; Vir, Singh, Nigam, & Jain, 2008). Dosage recommendations vary by age, gender, and pregnancy status, but WHO recommends daily IFA supplementation among non-pregnant women of reproductive age in countries where prevalence exceeds 40% (WHO, 2016).

In India, where over half of women of reproductive age are anemic (International Institute for Population Sciences, 2017), the promotion of iron folic acid to children and

For peer review only - http://bmjopen.bmj.com/site/about/guidelines.xhtml

women of reproductive age has been a part of the nutritional agenda since 1970 (Kumar, 1999; Bellad, Kapil, & Gupta, 2018). However the expansion of promotion to non-pregnant, out-of-school women only began in 2013 through the National Iron Plus Initiative (Kapil & Bhadoria, 2014). Despite India's life-course approach to promote iron supplements to all women its consumption remains low and anemia prevalence is still high (IIPS, 2017; Nguyen, Scott, Avula, Tran, Menon, 2018). This may be due to gaps in current iron supplement distribution practices of the India health system. Distribution outside of clinical settings target pregnant or in-school children; frontline workers provide tablets to pregnant women, and adolescent girls can get iron supplements weekly in school. Out-of-school, non-pregnant women do not have this access and must first go to a health facility to get tested before receiving iron supplements (UNICEF, 2018; Rai, Fawzi, Barik, & Chowdhury, 2018).

This process requires women to recognize they may be at risk for anemia. Yet, fatigue, one of the primary recognizable symptoms of anemia, may not be understood as a manifestation of the illness among women in India (Bager, 2014; Sobrero et al., 2001). Less than 20% of women who are aware of anemia in the state of Odisha, India recognize tiredness or weakness as symptom of anemia (UNICEF, 2018). Additionally, a 2017 study among pregnant women in rural parts of India found that women who recognized that they were fatigued still did not seek medical attention (Vincent et al., 2017). Studies in other parts of the world with high anemia prevalence have found that culture and gender norms around health responsibility can shape perceptions of anemia, which can in turn influence treatment-seeking behaviors among non-pregnant women (Bedwell et al., 2017). Reasons for the persistent high prevalence of anemia in India may be a failure to

For peer review only

understand fatigue as a cue to seek medical care, particularly among those who are non-pregnant.

We explore the extent to which women perceive fatigue as a cue to obtain medical care among women of reproductive age in Odisha, India. While the literature around the conceptualization of fatigue as a symptom of illness is limited, recent research in India have shown that inequitable gender roles may indirectly affect a woman's ability or desire to access iron supplements (Chaterjee & Fernandes, 2017; Diamond-Smith, Gupta, Kaur, & Kumar, 2016; Aurino, 2017; Bedwell et al., 2017; Jose, 2011). Other studies also show that women in Odisha tend to have less power within the household than their husbands, and carry the majority of the domestic workload burden (Seymour, 2010). As daily life is characterized by large amounts of physical labor, fatigue may be considered a normal part of everyday life among women of reproductive age, and thus, not a cue to obtain iron supplements. Clearly, knowledge about the prevalence and risks of anemia is critical as risk perception may be connected to gender roles. If women believe that their role as women is to bear fatigue and not to worry about prevention efforts, increasing risk perception may be an important first step.

Additionally, studies in India have highlighted the influence of shared-decision making within households as a key factor in anemia prevalence among women (Krupp et al., 2018; Varghese & Roy, 2019). How this relates to different conceptualizations of fatigue by various household members – husbands, mothers-in-law, and others – has not yet been explored, to the best of our knowledge. Yet, this seems like an important consideration: If women themselves do not equate fatigue with a need to seek medical

For peer review only - http://bmjopen.bmj.com/site/about/guidelines.xhtml

care, do influential others in her home view fatigue in the same manner? Hence, our research questions are:

RQ1. Are perceptions of fatigue a cue to obtain iron supplements among women of reproductive age in Odisha, India?

RQ2. What are the differences in perceptions of fatigue across women's referent groups (their mothers-in-law and husbands)?

Methods

We report findings from two methods, one quantitative and the other qualitative, used in this paper. First, we used perceptual mapping techniques to understand how our participants conceptualized fatigue. Subsequently, we conducted focus group discussions to determine the extent to which findings from the quantitative method could be triangulated with those from the quantitative method.

Perceptual Mapping

We used multiple methods to understand how conceptualizations of fatigue may relate with iron supplement-seeking behaviors among women of reproductive age in Odisha, India. We explore the extent to which women perceive fatigue as a cue to obtain medical care (Research Question 1). To answer this question, we used perceptual mapping, a marketing technique that depicts how participants perceive items in relation to one another. Perceptual mapping is often used in commercial marketing (Hastings,

For peer review only - http://bmjopen.bmj.com/site/about/guidelines.xhtml

Angus, & Bryant, 2011; Adaval, Coupey, & Narayanan, 2015; Iacobucci, Grisaffe, & DeSarbo, 2017), however it has recently gained momentum in health behavior communication (Pivonka, Seymour, McKenna, Baxter, & Williams, 2011; Bass et al., 2018; Van Voorhees et al., 2010; Ruggieri, Bass, Alhajji, & Gordon, 2018). In the current study, we expand on the public health application of perceptual mapping by using this technique to understand how women understand fatigue in relation to items associated with the acquisition of iron tablets. We follow the perceptual mapping activity with a card sorting exercise to understand if cognitive (i.e. perceived importance to health) and affective (i.e. dislike) perceptions could inform the results of the perceptual maps.

Participants

We recruited and randomly selected WRA (n=30), MIL (n=30), and men (n=30) from the Angul District of Odisha, India to participate in the study. All participants were above the age of 18, lived in the Angul District, and speak Odia.

Patient and public involvement

This study did not involve patients or the public in its development or implementation. Upon completion of the study, results were shared with various stakeholders at a two-day public convening in Bhubaneswar, Odisha, India.

Procedure

The George Washington University Institutional Review Board (IRB) and a local ethics board, D-COR Consulting Institutional Ethics Committee (IEC) reviewed and approved this study before recruitment began. During the perceptual mapping exercise, we showed participants two cards at a time, each with a picture of a different anemia-

For peer review only - http://bmjopen.bmj.com/site/about/guidelines.xhtml

related item, and we asked them to compare how similar the two were to each other. We recorded their rating on a scale from 1 to 4, where 4 indicated the most similarity. We had 12 cards in all, and all 66 permutations (1 versus 2, 1 versus 3, … 11 versus 12) were shown to each participant.

We also asked participants to do a card sorting exercise upon completing the perceptual mapping exercise. In this portion of the study, we asked participants to place the same cards into three piles based on the items level of importance for their health and well-being. Upon completing this task, participants were then asked to sort the cards into another set of three piles based on the likability of the item. Data collectors recorded the compositions of each pile.

Instruments

We used large cards with the images of interest printed on them. We selected images based on discussions with local researchers and a review of the literature to ensure images fit the cultural context. The 12 cards included an image of one of the following: IFA tablets, a clinic, pharmaceutical kiosks, women empowerment groups known as self-help groups, traditional healer, physician, fatigue, prenatal care, green vegetable, meat/fish, pregnancy, or stomach ache. Labels of each item were also included on each card. Our colleagues in Odisha, India assessed the cards for face validity.

Analysis

Participant ratings were recoded to indicate dissimilarity and then organized into matrixes. We used multidimensional scaling (MDS) to graphically plot the averaged

For peer review only - http://bmjopen.bmj.com/site/about/guidelines.xhtml

dissimilarities of the overall sample as well as each subgroup recorded (i.e., women of reproductive age, mothers-in-law, and men; Figures 1-3). MDS is a common method to analyze perceptual mapping data (Bijmolt & Wedel, 1999). Theoretically, the resulting maps can display items on multiple axes to represent underlying dimensions of perception. For simplicity and ease of interpretation, they are often distilled down to two (*x* and *y*) dimensions. The maps display dissimilarity so that the further two points are from each other, the more the participants perceived dissimilarity between the two items. Conversely, the closer the two points are on the map, the more the participants perceived similarity.

We converted the responses from the card sorting exercise into ranks from 0-2, where a score of 0 was given to cards that were placed in the “not very important to health” pile or the “dislike” pile, and a score of 2 was given to the cards that were in the “very important to health” pile or “like a lot” pile, for their respective parameters. Scores for liking were reverse coded so that higher values indicated a dislike. We analyzed the card sorting data using an ANOVA across the three participant groups. We conducted post-hoc analyses to determine homogenous subsets when omnibus differences were detected. There we no missing responses. All analysis were done in SPSS v.25.

Qualitative Method

Our qualitative method comprised focus group discussions.

Instrument development. We developed the interview and focus group guides based on the project’s theoretical frameworks, a review of the literature, and feedback from co-investigators in India. Interview guides covered general questions about what

For peer review only - http://bmjopen.bmj.com/site/about/guidelines.xhtml

women do on a typical day, their concerns and aspirations, and roles in the family and community.

Sampling. We used a random sampling procedure to select participants for focus group discussions. To do so, researchers first tabulated all the homes in each of the four villages where we worked and obtained the total number of eligible participants. Based on the number of participants needed for the focus groups, against a sampling frame that consisted of the entire village, we used a proportional skip pattern that began with a randomly selected initial participant to identify households from which to select every succeeding participant for each sub-group.

Data collection. We conducted 16 focus groups with women of reproductive age, mothers-in-law, adolescent girls, and husbands (n = 124). The sample description is published elsewhere (under review).

Trained researchers (who were native speakers) conducted all focus groups, and we matched interviewer and interviewee on gender. Audio recordings were transcribed in Odiya and subsequently translated into English for data analysis.

Analysis. We uploaded transcripts and the codebook into NVivo v.12 for analysis (Nvivo, 2015). We developed a draft codebook then applied a thematic analysis to characterize and subsequently modify emergent themes through an iterative approach that combined data collection and analysis concurrently (Guest, 2011). This allowed us to determine when saturation occurred, when no new themes emerged from the data.

Results

Sample

For peer review only - http://bmjopen.bmj.com/site/about/guidelines.xhtml

The demographic distribution of each subgroup can be found in Table 1. The average age of men in the sample was 35 years (*SD* = 6.00), and that of women was 28 years (*SD* = 4.76). The average age of mothers-in-law was 56 years (*SD* = 7.58). The majority of women of reproductive age had completed at least secondary school, while the majority of men had not completed secondary school. The majority of mothers-in-law had not received any education. Additionally, the majority of participants had at least two children. All but one participant was also married; the singular participant who was unmarried was widowed. All participants were Hindu. Almost all of the participants belonged to a scheduled caste, tribe, or "other backwards caste."

While the majority of women of reproductive age had taken iron folic acid in the past (86.7%), very few were taking it at the time of the survey (10%). Only about a quarter of mothers-in-law had ever taken iron folic acid, but 9.1% were taking it at the time of the survey. None of the men surveyed reported currently taking iron folic acid, and only 13.3% had ever taken it. About half of women of reproductive age had ever received an anemia diagnosis, and 14.3 reported currently being anemic at the time of the survey. The demographic distribution of participants who took part in the focus group discussion was very similar to that of the participants who took part in the perceptual mapping activity. This table is published elsewhere.

Perceptions of Fatigue

Perceptual maps. The perceptual maps for women of reproductive age show a conceptual separation, as evidenced by their spatial distance, between fatigue and the practical IFA distribution channels for non-pregnant women; one quadrant of the map

For peer review only - http://bmjopen.bmj.com/site/about/guidelines.xhtml

contains fatigue, while a separate one contains the items related to the iron supplement distribution channels for non-pregnant women, including physicians, clinics, and pharmaceutical kiosks (Figure 1). These three items are clustered together in one quadrant, indicating that they are perceived as similar to each other. Iron folic acid is also plotted in the same quadrant as this cluster, indicating that women of reproductive age may conceptualize physicians, clinics, and pharmaceutical kiosks as links to iron supplements. The separation of fatigue from these iron supplement distribution channels indicates that fatigue may not act as a cue to obtain iron supplements.

Card Sorting Exercise. Confirming these findings, the card sorting exercise showed that women of reproductive age were less likely to report fatigue as important to health than the items related iron supplement distribution channels for non-pregnant women (Table 2); fatigue was considered to be only somewhat important to health by women ($M = 0.77$, $SD = 0.73$), while the iron supplement channels, including physicians ($M = 1.80$, $SD = 0.55$), clinics ($M = 1.80$, $SD = 0.48$), and pharmaceutical kiosks ($M = 1.77$, $SD = 0.50$), were considered very important to health.

Differences in the conceptualization of fatigue and the items related to iron supplement distribution channels for non-pregnant women may also be explained by differences in affective ratings (Table 2). Fatigue was reported to be disliked by women ($M = 1.47$, $SD = 0.68$), while the other IFA channel-related items were hardly disliked at all (physician: $M = 0.20$, $SD = 0.41$; clinics: $M = 0.23$, $SD = 0.50$; pharmaceutical kiosks: $M = 0.27$, $SD = 0.58$).

For peer review only - http://bmjopen.bmj.com/site/about/guidelines.xhtml

Focus Group Discussions. WRA reported that fatigue, and oftentimes, extreme fatigue, was a normal part of daily life. Women accepted fatigue as part of their role as wives and mothers; they take care of all of the household responsibilities including all care for the children, serve their husband and in-laws, and often work outside of the home to earn extra money for the family. One woman said, "We have to work all the time, go to the mountains and fields, have children to take care of. We get no time to rest. We work in unhappiness and eat in pain." Women did not think of fatigue as something that they could change and they did not associate their own fatigue with the possibility of having anemia. Although most women knew that fatigue is a symptom of anemia, they underestimated the prevalence of anemia in their village despite endemic rates. A frontline health worker said, "In our village, in a year, 2-3-4 women have it [anemia]...mainly pregnant women." This is, of course, a far cry from what the actual numbers would be, based on data from the National Family Health Survey (IIPS, 2017), which shows that over half of women of reproductive age are anemic.

For peer review only

Perceptions of Fatigue across WRA Referent Groups

Perceptual maps. Men (Figure 2) and mothers-in-law (Figure 3) also perceived fatigue as different from the other items related to the iron supplement distribution channels for non-pregnant women. The perceptual map generated according to men's responses displays physicians, pregnancy, and prenatal care as plotted closely together in the same quadrant. This cluster also contains iron folic acid, indicating that men understand these items and iron supplements as existing in the same cognitive space. Similarly, the perceptual map generated for mothers-in-law displays physicians, clinics,

For peer review only - http://bmjopen.bmj.com/site/about/guidelines.xhtml

and pharmaceutical kiosks as a cluster plotted near the plot-point for iron folic acid. Fatigue, however, is plotted in a separate quadrant from these items related to the iron supplement distribution channels for non-pregnant women. The separation of fatigue from these channels indicates that fatigue may not act as a cue to obtain iron supplements among husbands and mothers-in-law.

Card Sorting Exercise. We also explored differences in perception across participant groups through the card sorting analysis. Perceptions of importance to health didn't differ across participant groups for any of the anemia-related items shown, with the exception of fatigue ($F = 9.66$, $p < 0.001$) and stomachache ($F = 5.76$, $p < 0.01$), which were significantly different between women of reproductive age and their referent groups. Affective perceptions (i.e. dislike) related to fatigue were also statistically different across the three groups as well ($F = 8.29$, $p < 0.001$).

Post-hoc Least Significant Differences (LSD) tests revealed that women and men had similar perceptions of fatigue across these cognitive and affective dimensions (i.e., importance to health and dislike), but MILs had significantly different perceptions. Fatigue was perceived with the same level of importance for health by both men ($M = 0.80$, $SD = 0.61$) and women ($M = 0.80$, $SD = 0.61$). However, mothers-in-law had significantly lower perceptions about the importance of fatigue ($M = 0.17$, $SD = 0.53$) than men ($p < 0.001$) and women ($p < 0.001$). Additionally, men reported a similar dislike of fatigue as women ($M = 1.23$, $SD = 0.73$, n.s.) while mothers-in-law disliked fatigue to a much greater extent ($M = 1.87$, $SD = 0.35$, $p < 0.05$).

Focus Group Discussions. Men acknowledged that women bear the brunt of the work at home and often face extreme fatigue but did not make the connection that anemia

For peer review only - http://bmjopen.bmj.com/site/about/guidelines.xhtml

could be at play. A husband from a focus group said, “They [women] don’t have time to do all these works. To do the outside works after completing the household works, they don’t get enough time.” Mothers-in-law felt that their daughters-in-law had it “easier” today than when they were young and showed less empathy than men for their daughters-in-law heavy workload. They also mentioned that younger women seek medical care more often than their generation. A mother-in-law said, “We never had those tablets [IFA]. We had no idea that we are pregnant until the 5th month but women of today know everything from the 1st month and run to the hospital.”

Discussion

This study aimed to understand the ways in which women conceptualized fatigue in order to determine if it is an adequate cue to seek iron supplements. Additionally, this study investigated perceptions of fatigue among mothers-in-law and husbands of women of reproductive age. We found that fatigue may not act as a sufficient cue for women to seek treatment for anemia. This was indicated by a perceptual distinction we observed between fatigue and the cluster of items related to the iron supplement distribution channels, which included physicians, clinics, and pharmaceutical kiosks. Additionally, the card sorting exercise showed cognitive and affective gaps in perceptions between fatigue, on the one hand, and the items related to the iron supplement distribution channels for non-pregnant women, on the other. In particular, women did not perceive fatigue as an important consideration for their health, although they reported dislike for it. If women do not perceive illness when fatigue is present, they may not seek treatment

For peer review only - http://bmjopen.bmj.com/site/about/guidelines.xhtml

(including asking for iron supplements) for it unless they are pregnant. This may exacerbate an existing gap in iron supplement distribution to non-pregnant, out-of-school women, who make up the largest proportion of women with anemia (IIPS, 2017).

We also investigated how fatigue is perceived among the women's referent groups within a household, namely their mothers-in-law and husbands. Men considered fatigue a low health concern, in a manner consistent with women themselves. Their mothers-in-law harbored attitudes that were even more dismissive of fatigue; they did not consider fatigue important to health at all. Studies show that mothers-in-law are involved in decision-making processes among anemic women in India (Ramachandran, Canny, Das, & Cutrell, 2010); among pregnant women, their support can greatly influence women's decision to obtain iron supplements (Varghese & Roy, 2019; Das & Sarkar, 2014). If mothers-in-law continue to believe and perpetuate the idea that fatigue is not a symptom of illness, women of reproductive age will also follow suit. Even in situations where the women of reproductive age does not agree with her mother in law, it may be difficult for her to obtain IFA against her mother in laws requests. Although the literature on the influence of mothers-in-law on the health-seeking behaviors of their daughters-in-law, studies have shown that is difficult for women to challenge their mothers-in-law's wishes (Allendorf, 2013). Therefore, in line with the recommendations of other studies, we highlight the importance of shifting mothers-in-law's perceptions of fatigue (Varghese & Roy, 2019). Shifting these perceptions in mothers-in-law can translate to shifted perceptions in women of reproductive age. Additionally, it is imperative that the mother-in-law does not act as a barrier for iron supplementation when a woman of reproductive age recognizes her fatigue as a symptom of anemia.

For peer review only - http://bmjopen.bmj.com/site/about/guidelines.xhtml

FATIGUE AS A CUE TO SEEK TREATMENT

Additionally, our qualitative results help to explain our perceptual mapping findings. Unequal gender roles that place the majority of household duties on the woman may normalize fatigue in women and negatively affecting women's risk perception, thus fatigue is not a cue to seek treatment. Gender norms are a sub-set of social norms that describe how people of a particular gender are expected to behave, in a given social context (ALIGN, 2019). As stated earlier, gender norms may contribute to the perceptions of illness, including fatigue (Chaterjee & Fernandes, 2017; Diamond-Smith, Gupta, Kaur, & Kumar, 2016; Aurino, 2017; Bedwell et al., 2017; Jose, 2011). Women are expected to carry the majority of the household workload. Our focus group discussions showed that women prioritize the wellbeing of their family over their own. Thus, they accept the fatigue that they feel as a part of their household role.

To our knowledge, this is the first study that explores the gaps in IFA distribution for non-pregnant women through an examination of the social context of Indian women living in Odisha. The current available health services require that non-pregnant, out-of-school, WRA seek IFA for their anemia, rather than receive it unprompted. However, the most recognizable symptom of anemia, fatigue, is not a cue to obtain IFA. This study shows that fatigue is not an adequate cue to obtain IFA among non-pregnant women in Odisha, India, resulting in a failure to obtain IFA and treat their anemia. We recommend improving the available services to non-pregnant WRA so that they can obtain IFA through frontline workers. However, within the current health practice landscape of Odisha, India, we recommend increasing knowledge around the connection between fatigue and anemia among MILs and WRA to improve IFA use among WRA. Efforts that

For peer review only - http://bmjopen.bmj.com/site/about/guidelines.xhtml

promote perceptions of risk for anemia can also contribute on this front. Future studies should measure IFA acquisition and adherence in non-pregnant women.

Acknowledgments: This study was funded by the Bill and Melinda Gates Foundation under Grant OPP1182519. The funders had no role in study design, data collection and analysis, decision to publish, or preparation of the manuscript.

Author Contributions: HY, ES, and RR made substantial contributions to the conception or design of the work, the analysis, and the interpretation of the work. SP also contributed to the design of the study, and led the data acquisition of data with help from ES and RR. All authors were involved in drafting the work or revising it critically and the final approval of the version to be published. All authors are accountable for all aspects of the work.

Declaration Statement: The authors of this paper have no conflicts of interest to report.

Ethics statement: This study was reviewed and approved by the George Washington University Institutional Review Board (IRB) as well as D-COR Consulting Institutional Ethics Committee (IEC). Data collectors obtained informed consent from all participants in the study.

Data Availability: The data associated with this study can be made available upon request. Anyone who wishes to have access to the de-identified data from the perceptual mapping activity, card sorting activity, or focus group discussions can email the study data manager, Hagere Yilma (corresponding author), at hyilma@gwu.edu. All data is de-identified. Investigators are permitted to access the data for any research questions that have yet to be explored by the study team.

For peer review only - http://bmjopen.bmj.com/site/about/guidelines.xhtml

For peer review only

For peer review only - http://bmjopen.bmj.com/site/about/guidelines.xhtml

References

Adaval, R., Coupey, E., & Narayanan, S. (2015). Direct mapping of consumer perceptions. Paper presented at the *Proceedings of the 1995 Academy of Marketing Science (AMS) Annual Conference,* pp. 255-263.

Ahmed, F., Khan, M. R., Akhtaruzzaman, M., Karim, R., Williams, G., Torlesse, H., et al. (2010). Long-term intermittent multiple micronutrient supplementation enhances hemoglobin and micronutrient status more than iron folic acid supplementation in bangladeshi rural adolescent girls with nutritional anemia. *The Journal of Nutrition, 140*(10), 1879-1886.

Allen, L. H. (2000). Anemia and iron deficiency: Effects on pregnancy outcome. *The American Journal of Clinical Nutrition, 71*(5), 1284S.

Allendorf, K. (2013). Going nuclear? family structure and young women's health in india, 1992–2006. *Demography, 50*(3), 853-880.

Aurino, E. (2017). Do boys eat better than girls in india? longitudinal evidence on dietary diversity and food consumption disparities among children and adolescents. *Economics & Human Biology, 25*, 99-111.

Bass, S. B., Jessop, A., Maurer, L., Gashat, M., Al Hajji, M., & Gutierrez, M. (2018). Mapping the barriers and facilitators of HCV treatment initiation in methadone maintenance therapy patients: Implications for intervention development. *Journal of Health Communication, 23*(1), 117-127.

Bass, S. B., Wolak, C., Greener, J., Tedaldi, E., Nanavati, A., Ruppert, K., et al. (2016). Using perceptual mapping methods to understand gender differences in perceived

barriers and benefits of clinical research participation in urban minority HIV patients. *AIDS Care, 28*(4), 528-536.

Beard, J. L. (2000). Iron requirements in adolescent females. *The Journal of Nutrition, 130*(2), 442S.

Bedwell, R. M., Spielvogel, H., Bellido, D., & Vitzthum, V. J. (2017). Factors influencing the use of biomedical health care by rural bolivian anemic women: Structural barriers, reproductive status, gender roles, and concepts of anemia. *PloS One, 12*(1), e0170475.

Bellad, A., Kapil, R., & Gupta, A. (2018). National guidelines for prevention and control of iron deficiency anemia in india. *Indian Journal of Community Health, 30*(01), 89-94.

Benoist, B. d., McLean, E., Egll, I., & Cogswell, M. (2008). Worldwide prevalence of anaemia 1993-2005: WHO global database on anaemia. *Worldwide Prevalence of Anaemia 1993-2005: WHO Global Database on Anaemia.,*

Bijmolt, T. H., & Wedel, M. (1999). A comparison of multidimensional scaling methods for perceptual mapping.*Journal of Marketing Research, 36*(2), 277-285.

Brabin, B. J., Hakimi, M., & Pelletier, D. (2001). An analysis of anemia and pregnancy-related maternal mortality. *The Journal of Nutrition, 131*(2), 615S.

Cooke, A. G., McCavit, T. L., Buchanan, G. R., & Powers, J. M. (2017). Iron deficiency anemia in adolescents who present with heavy menstrual bleeding. *Journal of Pediatric and Adolescent Gynecology, 30*(2), 247-250.

For peer review only - http://bmjopen.bmj.com/site/about/guidelines.xhtml

Cooper, L. G. (1983). A review of multidimensional scaling in marketing research. *Applied Psychological Measurement, 7*(4), 427-450.

Das, A., & Sarkar, M. (2014). Pregnancy-related health information-seeking behaviors among rural pregnant women in india: Validating the wilson model in the indian context. *The Yale Journal of Biology and Medicine, 87*(3), 251.

Gardner, G. W., Edgerton, V. R., Senewiratne, B., Barnard, R. J., & Ohira, Y. (1977). Physical work capacity and metabolic stress in subjects with iron deficiency anemia. *The American Journal of Clinical Nutrition, 30*(6), 910-917.

Ghanekar, J., Kanani, S., & Patel, S. (2002). Toward better compliance with iron–folic acid supplements: Understanding the behavior of poor urban pregnant women through ethnographic decision models in vadodara, india. *Food and Nutrition Bulletin, 23*(1), 65-72.

Hallberg, L., Högdahl, A., Nilsson, L., & Rybo, G. (1966). Menstrual blood loss and iron deficiency. *Acta Medica Scandinavica, 180*(5), 639-650.

Iacobucci, D., Grisaffe, D., & DeSarbo, W. (2017). Statistical perceptual maps: Using confidence region ellipses to enhance the interpretations of brand positions in multidimensional scaling. *Journal of Marketing Analytics, 5*(3-4), 81-98.

International Institute for Population Sciences. (2017). *National family health survey (NFHS-4), 2015-16*. India. Mumbai: IIPS.

Jose, S. (2011). Adult undernutrition in india: Is there a huge gender gap? *Economic and Political Weekly,* , 95-102.

Kapil, U., & Bhadoria, A. S. (2014). National iron-plus initiative guidelines for control of iron deficiency anaemia in india, 2013. *Natl Med J India, 27*(1), 27-29.

Krawczyk, M., & Xiang, Z. (2016). Perceptual mapping of hotel brands using online reviews: A text analytics approach. *Information Technology & Tourism, 16*(1), 23-43.

Kumar, A. (1999). National nutritional anaemia control programme in india. *Indian Journal of Public Health., 43*(1), 3-5.

Milman, N. (2011). Postpartum anemia I: Definition, prevalence, causes, and consequences. *Annals of Hematology, 90*(11), 1247.

Nguyen, P. H., Sanghvi, T., Kim, S. S., Tran, L. M., Afsana, K., Mahmud, Z., et al. (2017). Factors influencing maternal nutrition practices in a large scale maternal, newborn and child health program in bangladesh. *PLoS One, 12*(7), e0179873.

Nguyen, P. H., Scott, S., Avula, R., Tran, L. M., & Menon, P. (2018). Trends and drivers of change in the prevalence of anaemia among 1 million women and children in india, 2006 to 2016. *BMJ Global Health, 3*(5), e001010.

Rai, R. K., Fawzi, W. W., Barik, A., & Chowdhury, A. (2018a). The burden of iron-deficiency anaemia among women in india: How have iron and folic acid interventions fared? *WHO South-East Asia Journal of Public Health, 7*(1), 18.

Ramachandran, D., Canny, J., Das, P. D., & Cutrell, E. (2010). Mobile-izing health workers in rural india. Paper presented at the *Proceedings of the SIGCHI Conference on Human Factors in Computing Systems,* pp. 1889-1898.

For peer review only - http://bmjopen.bmj.com/site/about/guidelines.xhtml

Ruggieri, D. G., Bass, S. B., Alhajji, M., & Gordon, T. F. (2018). Understanding parents' perceptions of school-based BMI screening and BMI report cards using perceptual mapping: Implications for school nurses. *The Journal of School Nursing,* , 1059840518789243.

Sobrero, A., Puglisi, F., Guglielmi, A., Belvedere, O., Aprile, G., Ramello, M., et al. (2001). Fatigue: A main component of anemia symptomatology. Paper presented at the *Seminars in Oncology, , 28.* pp. 15-18.

Strauss, W. E., & Auerbach, M. (2018). Health-related quality of life in patients with iron deficiency anemia: Impact of treatment with intravenous iron. *Patient Related Outcome Measures, 9*, 285.

UNICEF, & AIIMS. (2018). *Process documentation of national iron plus initiative (NIPI) – towards an anaemia free odisha, 2016-17*. Bhubaneswar, Odisha: UNICEF.

Varghese, R., & Roy, M. (2019). Coresidence with mother-in-law and maternal anemia in rural india. *Social Science & Medicine, 226*, 37-46.

Vincent, A., Keerthana, K., Damotharan, K., Newtonraj, A., Bazroy, J., & Manikandan, M. (2017). Health care seeking behaviour of women during pregnancy in rural south india: A qualitative study. *International Journal of Community Medicine and Public Health, 4*(10), 3636-3639.

Vir, S. C., Singh, N., Nigam, A. K., & Jain, R. (2008). Weekly iron and folic acid supplementation with counseling reduces anemia in adolescent girls: A large-scale effectiveness study in uttar pradesh, india. *Food and Nutrition Bulletin, 29*(3), 186-194.

For peer review only - http://bmjopen.bmj.com/site/about/guidelines.xhtml

Wiradnyani, L. A. A., Khusun, H., Achadi, E. L., Ocviyanti, D., & Shankar, A. H. (2016). Role of family support and women's knowledge on pregnancy-related risks in adherence to maternal iron–folic acid supplementation in indonesia. *Public Health Nutrition, 19*(15), 2818-2828.

World Health Organization. (2014). *Global nutrition targets 2025: Anaemia policy brief* World Health Organization.

World Health Organization. (2016). *Guideline: Daily iron supplementation in adult women and adolescent girls*. Geneva, Switzerland: World Health Organization.

Wouters, H. J., van der Klauw, Melanie M, de Witte, T., Stauder, R., Swinkels, D. W., Wolffenbuttel, B. H., et al. (2019). Association of anemia with health-related quality of life and survival: A large population-based cohort study.*Haematologica, 104*(3), 468-476.

ew only

For peer review only - http://bmjopen.bmj.com/site/about/guidelines.xhtml

Table 1. Demographic Information- Perceptual Mapping and Card Sorting

	**Men** (n=30)	**WRA** (n=30)	**Mothers-in-Law** (n=30)
	M (SD)	***M (SD)***	***M (SD)***
**Age**	34.67 (6.00)	27.90 (4.76)	55.87 (7.58)
	**n (%)**	**n (%)**	**n (%)**
**School**			
None	3 (10)	4 (13.3)	21 (70.0)
Up to Primary	12 (40)	8 (26.7)	6 (20.0)
Up to Secondary	6 (20)	7 (23.3)	2 (6.7)
Up to High Secondary	5 (16.6)	10 (33.4)	1 (3.3)
Up to Tertiary	4 (13.3)	1 (3.3)	0 (0)
**Married**	30 (100)	30 (100)	29 (96.7)[a]
**Religion-Hindu**	100 (100)	100 (100)	100 (100)
**Caste**			
Scheduled Caste/ Tribe	18 (60.0)	16 (53.3)	17 (56.6)
Other Backwards Caste	11 (36.7)	12 (40.0)	13 (43.3)
Other Caste	1 (3.3)	2 (6.7)	0 (0)
**Children**			
None	0 (0)	3 (10)	0 (0)
1	4 (13.3)	7 (23.3)	3 (10.0)
2	12 (40.0)	10 (33.3)	3 (10.0)

For peer review only

	Men	WRA	Mothers-in-Law
3	10 (33.3)	7 (23.3)	11 (36.7)
4 or more	4 (13.3)	3 (10.0)	13 (43.3)
**Ever taken IFA**	4 (13.3)	26 (86.7)	7 (23.3)
**Currently taking IFA**	0 (0)	3 (10.0)	2 (9.1)
**Diagnoses of anemia ever**	1 (3.3)	16 (53.3)	4 (13.3)
**Currently anemic?**	5 (21.7)	4 (14.3)	3 (13.0)

Note: Under "School", "I" indicates incomplete and "C" indicates complete; [a] The one mother-in-law who wasn't married was a widow.

*p < 0.05, **p < 0.01, ***p < 0.001

For peer review only

Table 2. Importance to Health and Dislike Rating Across Participant Category

		WRA	Males	MILs	
Item	Rating Parameter	*M* (SD)	*M* (SD)	*M* (SD)	*F*
IFA pill	Importance	1.67 (0.68)	1.87 (0.35)	1.73 (0.52)	2.39
	Dislike	0.53 (0.73)	0.20 (0.48)	0.47 (0.73)	2.15
Clinic	Importance	1.80 (0.48)	1.93 (0.25)	1.83 (0.38)	0.98
	Dislike	0.23 (0.50)	0.20 (0.41)	0.23 (0.50)	0.05
Kiosk	Importance	1.77 (0.50)	1.93 (0.25)	1.87 (0.35)	1.44
	Dislike	0.27 (0.58)	0.17 (0.38)	0.13 (0.43)	0.64
Self-Help Groups	Importance	1.37 (0.72)	1.27 (0.74)	1.57 (0.68)	1.38
	Dislike	0.30 (0.47)[a]	0.73 (0.74)[b]	0.50 (0.68)[a,b]	3.44*
Traditional healer	Importance	0.80 (0.76)	1.23 (0.82)	1.03 (0.67)	2.50
	Dislike	1.07 (0.78)	0.97 (0.85)	1.07 (0.69)	0.17
Physician	Importance	1.80 (0.55)	2.00 (0)	1.90 (0.31)	2.27
	Dislike	0.20 (0.41)	0.10 (0.31)	0.20 (0.48)	0.61
Fatigue	Importance	0.77 (0.73)[a]	0.80 (0.61)[a]	0.17 (0.53)[b]	9.66***
	Dislike	1.47 (0.68)[a]	1.23 (0.73)[a]	1.87 (0.35)[b]	8.29***
Prenatal care	Importance	1.43 (0.73)	1.40 (0.67)	1.67 (0.61)	1.40
	Dislike	0.70 (0.89)	0.57 (0.50)	0.33 (0.66)	2.12
Green veggies	Importance	1.63 (0.62)	1.63 (0.49)	1.80 (0.41)	1.06
	Dislike	0.37 (0.61)	0.30 (0.60)	0.13 (0.35)	1.52
Meat	Importance	1.23 (0.68)	0.97 (0.76)	1.37 (0.61)	2.62
	Dislike	0.77 (0.73)	0.90 (0.76)	0.60 (0.62)	1.36

For peer review only - http://bmjopen.bmj.com/site/about/guidelines.xhtml

Pregnancy	Importance	1.13 (0.82)	1.23 (0.63)	1.57 (0.68)	3.04
	Dislike	0.80 (0.85)[a]	0.80 (0.48)[a]	0.30 (0.60)[b]	5.74**
Stomach-ache	Importance	0.63 (0.67)[a]	0.87 (0.68)[a]	0.30 (0.60)[b]	5.76**
	Dislike	1.47 (0.68)[a]	1.17 (0.65)[b]	1.83 (0.46)[c]	9.15***

Note: The letters a and b denote homogenous subsets yielded from the post-hoc analysis when omnibus significance was detected.

*p < 0.05, **p < 0.01, ***p < 0.001

For peer review only

Figure Captions.

Figure 1. Perceptual Map derived for Women of Reproductive Age. Dissimilarity plot across two-dimensions (corresponding to the x and y axis) as displayed by SPSS v. 25; plot point labels have been edited for reader clarity.

Figure 2. Perceptual Map derived for Males. Dissimilarity plot across two-dimensions (corresponding to the x and y axis) as displayed by SPSS v. 25; plot point labels have been edited for reader clarity.

Figure 3. Perceptual Map derived for Mothers-in-Law. Dissimilarity plot across two-dimensions (corresponding to the x and y axis) as displayed by SPSS v. 25; plot point labels have been edited for reader clarity.

For peer review only

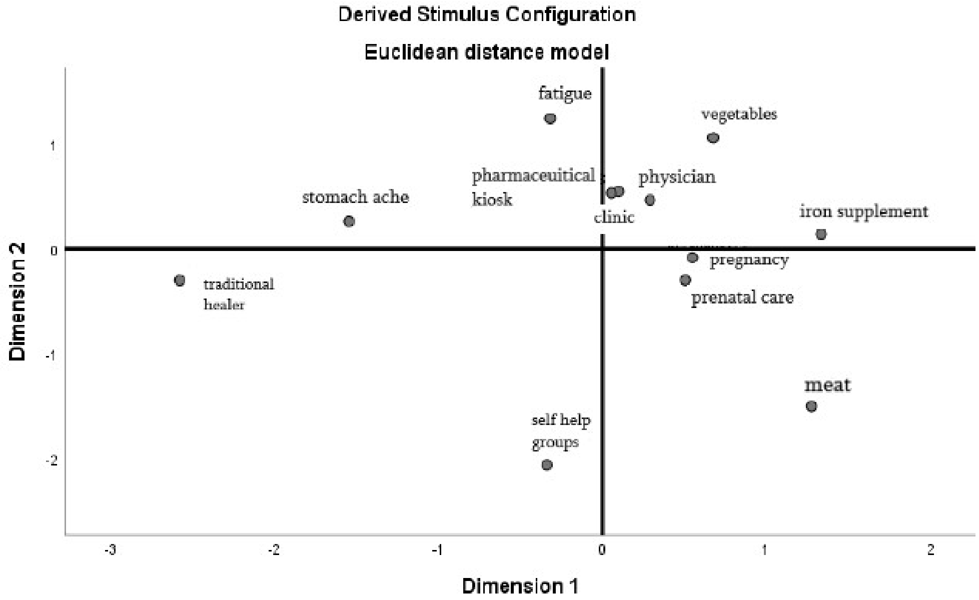

Figure 1. Perceptual Map derived for Women of Reproductive Age. Dissimilarity plot across two-dimensions (corresponding to the x and y axis) as displayed by SPSS v. 25; plot point labels have been edited for reader clarity.

171x107mm (96 x 96 DPI)

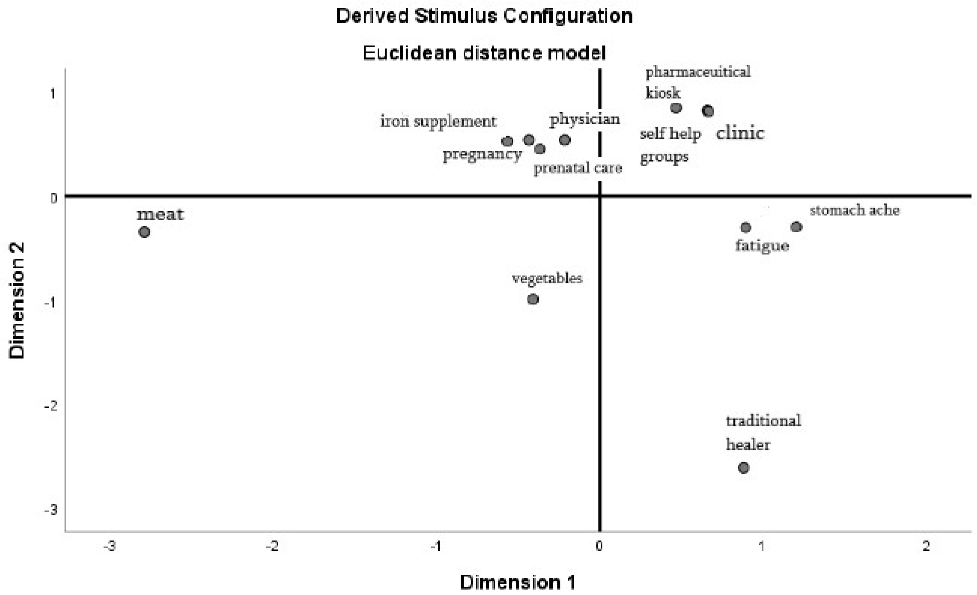

Figure 2. Perceptual Map derived for Males. Dissimilarity plot across two-dimensions (corresponding to the x and y axis) as displayed by SPSS v. 25; plot point labels have been edited for reader clarity.

175x109mm (96 x 96 DPI)

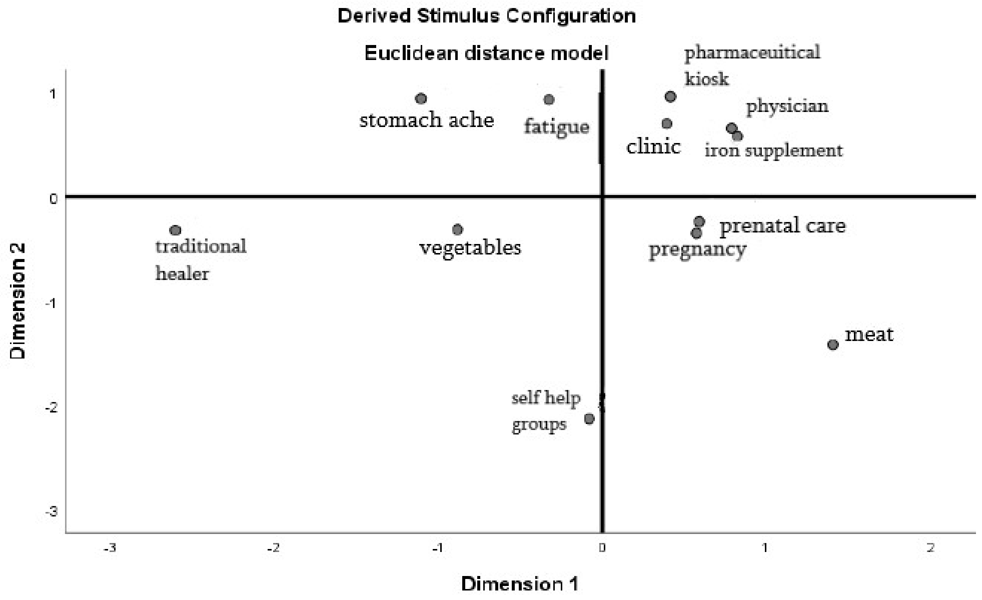

Figure 2. Perceptual Map derived for Males. Dissimilarity plot across two-dimensions (corresponding to the x and y axis) as displayed by SPSS v. 25; plot point labels have been edited for reader clarity.
Figure 3. Perceptual Map derived for Mothers-in-Law. Dissimilarity plot across two-dimensions (corresponding to the x and y axis) as displayed by SPSS v. 25; plot point labels have been edited for reader clarity.

177x111mm (96 x 96 DPI)

BMJ Open

Is fatigue a cue to obtain iron supplements in Odisha India? A mixed methods investigation

Journal:	*BMJ Open*
Manuscript ID	bmjopen-2020-037471.R1
Article Type:	Original research
Date Submitted by the Author:	29-May-2020
Complete List of Authors:	Yilma, Hagere; Prevention and Community Health<br>Sedlander, Erica; George Washington University Milken Institute of Public Health, Prevention and Community Health<br>Rimal, Rajiv; Johns Hopkins Bloomberg School of Public Health Center for Teaching and Learning, Health, Behavior and Society<br>Pattnaik, Soumik; D-COR Consulting
**Primary Subject Heading**:	Global health
Secondary Subject Heading:	Public health, Global health
Keywords:	PUBLIC HEALTH, QUALITATIVE RESEARCH, Nutrition < TROPICAL MEDICINE, Anaemia < HAEMATOLOGY

SCHOLARONE™
Manuscripts

I, the Submitting Author has the right to grant and does grant on behalf of all authors of the Work (as defined in the below author licence), an exclusive licence and/or a non-exclusive licence for contributions from authors who are: i) UK Crown employees; ii) where BMJ has agreed a CC-BY licence shall apply, and/or iii) in accordance with the terms applicable for US Federal Government officers or employees acting as part of their official duties; on a worldwide, perpetual, irrevocable, royalty-free basis to BMJ Publishing Group Ltd ("BMJ") its licensees and where the relevant Journal is co-owned by BMJ to the co-owners of the Journal, to publish the Work in this journal and any other BMJ products and to exploit all rights, as set out in our licence.

The Submitting Author accepts and understands that any supply made under these terms is made by BMJ to the Submitting Author unless you are acting as an employee on behalf of your employer or a postgraduate student of an affiliated institution which is paying any applicable article publishing charge ("APC") for Open Access articles. Where the Submitting Author wishes to make the Work available on an Open Access basis (and intends to pay the relevant APC), the terms of reuse of such Open Access shall be governed by a Creative Commons licence – details of these licences and which Creative Commons licence will apply to this Work are set out in our licence referred to above.

Other than as permitted in any relevant BMJ Author's Self Archiving Policies, I confirm this Work has not been accepted for publication elsewhere, is not being considered for publication elsewhere and does not duplicate material already published. I confirm all authors consent to publication of this Work and authorise the granting of this licence.

For peer review only

Title: Is fatigue a cue to obtain iron supplements in Odisha India? A mixed methods investigation

Authors: Hagere Yilma, MPH[1], Erica Sedlander, DrPH, MPH[1], Rajiv N. Rimal, PhD[2], Soumik Pattnaik, MS[3]

[1] George Washington University, Milken Institute School of Public Health, Department of Prevention and Community Health
[2] Johns Hopkins University Bloomberg School of Public Health, Health, Behavior, and Society
[3] DCOR Consulting

Hagere Yilma, MPH (corresponding author)
950 New Hampshire, Department of Prevention and Community Health
202.994.1486
https://orcid.org/0000-0002-4042-3484
hyilma@gwu.edu

Erica Sedlander, DrPH, MPH
950 New Hampshire, Department of Prevention and Community Health
202.994.0849
esedlander@gwu.edu

Rajiv N. Rimal, PhD
624 N. Broadway, Department of Health, Behavior, and Society
rimal@jhu.edu

Soumik Pattnaik, MS
H.B Colony, Punjabi Colony, Satya Nagar, Bhubaneswar, Odisha, India
+91 94384 63984
soumik.dcor@gmail.com

For peer review only

Abstract

Objectives: This study aims to understand if the main symptom of anemia, fatigue, is a health concern that can cue women of reproductive age and their referent groups to obtain iron supplements.

Design: This is a mixed methods study that consisted of a cross-sectional perceptual mapping and card sorting activity along with 16 focus group discussions with women of reproductive age, mothers-in-law, and men. Participants of the perceptual mapping and card sorting activity were asked to compare images of anemia related items and concepts. Participants of the focus group discussion were asked about the average life of a women in Odisha, aspirations among women, and perceptions of anemia and iron supplementation in the community. The quantitative methods were analyzed through multidimensional scaling and ANOVAs in SPSS. The qualitative methods were analyzed through applied thematic analysis using NVIVO.

Setting: Bhubaneswar, Odisha, India

Participants: Women of reproductive age (n =30), mothers-in-law (n = 30), and married men (n = 30) were randomly selected to participate in the perceptual mapping and card sorting exercise. A separate sample of each group was randomly selected for the focus group discussions (n = 148).

Primary and secondary outcome measures: We collected perceptions of dissimilarity between anemia-related items, including fatigue and medical items; the extent to which these items were perceived as important to health or likable; qualitative information about gender norms.

Results: Cognitive maps and card sorting revealed that fatigue was conceptualized distinctly from items related to medical treatment and that perceptions around fatigue's

importance to health were low. Women from the focus groups reported that fatigue is a part of their daily life.

Conclusion: Our results indicate that fatigue is currently not an adequate cue to seek treatment, perhaps due to the normalization of fatigue as a part of women's daily life.

Keywords: anemia; gender norms; iron folic acid; perceptual mapping; women's health

For peer review only

For peer review only - http://bmjopen.bmj.com/site/about/guidelines.xhtml

Strengths and Limitations of the study

- We adapted a marketing methodology known as perceptual mapping, coupled with a card sorting exercise and focus group discussions to understand how women of reproductive age, and their relevant referent groups (i.e., mothers-in-law and men) perceive fatigue.
- Qualitative focus groups were used to minimize the ambiguity of the perceptual mapping plots.
- The size of each subsample was small (n=30 each), reducing our ability to find significant differences from the card sorting activity.

For peer review only

Background

Anemia is a condition characterized by a low red blood cell count that affects a quarter of all people worldwide.[1] If left untreated, anemia can lead to a number of poor health outcomes, including poor mental health,[2-3] reduced work capacity,[4] pregnancy complications,[5-6] and sometimes death.[3] Women of reproductive age are the most vulnerable for anemia,[1] as blood loss during menstruation and childbirth raises their risk.[7-10] Recent research suggests that social structure may exacerbate anemia prevalence in women, particularly as it relates to gender norms.[11-14, 1] Gender norms are a subset of social norms, which refer to expectations around how people of a specific gender are expected to behave.[15]

The World Health Organization (WHO) has made anemia one of six global nutritional targets.[16] Among the primary recommendations for the treatment of anemia is iron and folic acid supplementation, an effective strategy in low- and middle- income countries.[17-19] Dosage recommendations vary by age, gender, and pregnancy status, but WHO recommends iron folic acid supplementation for non-pregnant women of reproductive age in countries where prevalence exceeds 40%.[17]

In India, where over half of women of reproductive age are anemic.[20] the promotion of iron folic acid to children and women of reproductive age has been a part of the nutritional agenda since 1970.[21-22] However the expansion of promotion to non-pregnant, out-of-school women only began in 2013 through the National Iron Plus Initiative.[23] Despite India's recent life-course approach to promote iron supplements to all women, its consumption remains low and anemia prevalence is still high.[20, 24] This may be due to gaps in current iron supplement distribution practices in the Indian health

For peer review only - http://bmjopen.bmj.com/site/about/guidelines.xhtml

system. Distribution outside of clinical settings target pregnant women or in-school adolescents; frontline workers provide tablets to pregnant women, and adolescent girls can get iron supplements weekly in school. Out-of-school, non-pregnant women do not have this access and must first go to a health facility to get tested before receiving treatment, which is typically iron supplementation for mild and moderate forms of anemia.[25, 26] This process requires an ability to detect anemia symptoms within oneself and seek medical care.

However, the primary manifestation of anemia, fatigue, may be ignored as a sign of illness.[27-28] In a review of the evolving conceptualizations of fatigue across time, Loriol (2017) notes that, historically, fatigue is considered a necessary outcome for physical energy expenditure and, consequently, commonly attributed to a day's hard work. The result is a prevailing social expectation that the more the one expends physical energy, the more fatigued one is. This is seen in the inverse association between fatigue and socioeconomic status.[29] Therefore, women who exert a lot of physical energy in a day may not feel their fatigue is cause for medical alarm. Rather, they may feel that the fatigue they experience is a normal outcome of their hard work.

It is no surprise then that more than 80% of women in Odisha, India do not recognize tiredness or weakness as symptom of anemia.[26] Studies in similar cultures have also shown that women may not readily associate fatigue with illness.[30] In fact, anemic women who reported fatigue during pregnancy did not seek medical attention because they believed their anemia-related fatigue was just a normal part of pregnancy.[31-32]

For peer review only

For peer review only - http://bmjopen.bmj.com/site/about/guidelines.xhtml

FATIGUE AS A CUE TO SEEK TREATMENT

While the anemia literature among non-pregnant women in India is sparse, research in other parts of the world has shown that fatigue represents a conceptually different dimension of symptoms than somatic symptoms, even when both forms share an underlying cause.[33-34] Further, Ball et al. (2017) demonstrate that women's perceptions of fatigue symptoms are influenced by familial factors, whereas men hold perceptions of fatigue that are influenced by genetic factors.[33] A study in an area with high anemia prevalence showed that culture and gender norms can shape perceptions of anemia, which can in turn influence treatment-seeking behaviors among non-pregnant women.[13] Therefore, a similar normalization of fatigue may exist among non-pregnant women in rural India, as expectations for labor may lead to exhaustion that is difficult to disentangle form anemia-related fatigue. In fact, fatigue is commonly associated with activities like caretaking and employment in women but not men.[35]

We explore the extent to which women perceive fatigue as a cue to obtain medical care among women of reproductive age in Odisha, India. While the literature around the conceptualization of fatigue as a symptom of illness is limited, recent research in India have shown that inequitable gender norms may indirectly affect a woman's ability or desire to access iron supplements.[11-14, 36] Other studies also show that women in Odisha tend to have less power within the household than their husbands, and carry the majority of the domestic workload burden.[37] As daily life is characterized by large amounts of physical labor (for both men and women), fatigue may be considered a normal part of everyday life among women of reproductive age, and thus, not a cue to obtain iron supplements. Clearly, knowledge about the prevalence and risks of anemia is critical as risk perception may be connected to gender norms. If women believe that

For peer review only - http://bmjopen.bmj.com/site/about/guidelines.xhtml

fatigue is just a necessary outcome of their role as women, rather than a symptom of illness, increasing risk perception may be an important first step.

Additionally, studies in India have highlighted the influence of shared-decision making within households as a key factor in anemia prevalence among women.[38-39] How this relates to different conceptualizations of fatigue by various household members – husbands, mothers-in-law, and others – has not yet been explored, to the best of our knowledge. Yet, this is an important consideration: If women themselves do not equate fatigue with a need to seek medical care, do influential others in her home view fatigue in the same manner? Hence, our research questions are:

RQ1. Are perceptions of fatigue a cue to obtain iron supplements among women of reproductive age in Odisha, India?

RQ2. What are the differences in perceptions of fatigue across women's referent groups (their mothers-in-law and husbands)?

Methods

We used multiple methods to understand how conceptualizations of fatigue may relate to treatment-seeking behaviors among women of reproductive age in Odisha, India. We report findings from two methods, one quantitative and the other qualitative, used in this paper. First, we used perceptual mapping techniques and a card sorting activity to understand how our participants conceptualized fatigue.

Perceptual mapping is often used in commercial marketing,[40-42] however it has recently gained momentum in health behavior communication.[43-47] In the current

For peer review only - http://bmjopen.bmj.com/site/about/guidelines.xhtml

study, we expand on the public health application of perceptual mapping by using this technique to understand how women understand fatigue in relation to items associated with the acquisition of iron tablets. We follow the perceptual mapping activity with a card sorting exercise to understand if cognitive (i.e. perceived importance to health) and affective (i.e. dislike) perceptions could inform the results of the perceptual maps.

While perceptual maps can highlight conceptual associations (or disassociations) between concepts, it is difficult to pinpoint the dimensions of perception around which these similarities or differences exist. We included a card sorting activity to probe the cognitive and affective dimensions of perception. However, a qualitative component is also needed to delineate why these associations or disassociations may exist. Therefore, we followed the quantitative methods with qualitative focus group discussions to triangulate our findings and identify the social and cultural underpinnings of how fatigue is conceptualized.

All components of this study come from formative research conducted for the Reduction in Anemia through Normative Innovations (RANI) Project. The RANI Project is implementing and evaluating a social norms-based anemia reduction intervention. The overall goals of the formative research components were larger than the research questions presented here; all of the goals of the formative research beyond this study are outlined the RANI project formative research protocol.[48]

Sampling

The perceptual mapping and card sorting activity collected data from a different sample than the focus group discussion to accommodate the larger formative research goals of the methods and limit participant burden. However, all samples came from the

For peer review only - http://bmjopen.bmj.com/site/about/guidelines.xhtml

Angul District of Odisha, India and shared the same random sampling procedure. To do so, researchers first tabulated all the homes in each of the four villages where we worked and obtained the total number of eligible participants. Based on the number of participants needed for the focus groups, against a sampling frame that consisted of the entire village, we used a proportional skip pattern that began with a randomly selected initial participant to identify households from which to select every succeeding participant for each sub-group. From the remaining households, a similar selection process was applied to select the participants for the perceptual mapping and card sorting activity.

We conducted 16 focus groups with women of reproductive age, mothers-in-law, adolescent girls, and husbands (n = 124). The sample description is published elsewhere.[11] The final sample for the perceptual mapping and card sorting exercise comprised women of reproductive age (n=30), mothers-in-law (n=30), and men (n=30). All participants were required to live in the Angul District, speak Odiya, and give written consent before participating. Parental permission was obtained for all participants younger than 18 years old. A small gift of a bowl and spoon was given as incentive for participating.

Patient and Public Involvement

This study did not involve patients or the public in its development or implementation. Upon completion of the study, results were shared with various stakeholders at a two-day convening in Bhubaneswar, Odisha, India.

Procedure

For peer review only - http://bmjopen.bmj.com/site/about/guidelines.xhtml

The XXX University Institutional Review Board (IRB) and a local ethics board, D-COR Consulting Institutional Ethics Committee (IEC) reviewed and approved this study before recruitment began.

Perceptual Mapping. During the perceptual mapping exercise, we showed participants two cards at a time, each with a picture of a different anemia-related item, and we asked them to compare how similar the two were to each other. We recorded their rating on a scale from 1 to 4, where 4 indicated the most similarity. We had 12 cards in all, and all 66 permutations (1 versus 2, 1 versus 3, … 11 versus 12) were shown to each participant. A more detailed explanation of the perceptual mapping procedure can be found in the Formative Research Protocol.[48]

Card Sorting. We also asked participants to do a card sorting exercise upon completing the perceptual mapping exercise. In this portion of the study, we asked participants to place the same cards into three piles based on the items level of importance for their health and well-being. Upon completing this task, participants were then asked to sort the cards into another set of three piles based on the likability of the item. Data collectors recorded the compositions of each pile.

Focus Group Discussions. Trained researchers (native speakers from Odisha India) conducted all focus groups, and we matched interviewer and interviewee on gender. For each focus group, one member of the research staff observed the group while taking notes and researchers wrote field notes after each interview. Audio recordings were transcribed in Odiya and subsequently translated into English for data analysis. A more detailed description of the focus group discussion procedures can be found in a separate study.[11]

For peer review only - http://bmjopen.bmj.com/site/about/guidelines.xhtml

Instruments

All instruments were developed based on discussions with local researchers and a review of the literature to ensure cultural and theoretical relevance.[25, 49-50] For the perceptual mapping and card sorting activity, we used large cards with the images of interest printed on them. The 12 cards included an image of one of the following: iron tablets, a clinic, pharmaceutical kiosks, women empowerment groups known as self-help groups, traditional healer, physician, fatigue, prenatal care, green vegetable, meat/fish, pregnancy, or stomach ache. Labels of each item were also included on each card. Our colleagues in Odisha, India assessed the cards for face validity. Interview guides for the focus group discussions covered general questions about what women do on a typical day, their concerns and aspirations, and roles in the family and community. The questionnaire for the perceptual mapping and card sorting activity (Supplement 1), along with the focus group discussion guides (Supplement 2) are included as supplementary material.

Analysis

Perceptual Maps. Participant ratings were recoded to indicate dissimilarity and then organized into matrixes. We used multidimensional scaling (MDS) to graphically plot the averaged dissimilarities of the overall sample as well as each subgroup recorded (i.e., women of reproductive age, mothers-in-law, and men; Figures 1-3). MDS is a common method to analyze perceptual mapping data.[51] Theoretically, the resulting maps can display items on multiple axes to represent underlying dimensions of perception. For simplicity and ease of interpretation, they are often distilled down to two (x and y) dimensions. The maps display dissimilarity so that the further two points are

For peer review only - http://bmjopen.bmj.com/site/about/guidelines.xhtml

from each other, the more the participants perceived dissimilarity between the two items. Conversely, the closer the two points are on the map, the more the participants perceived similarity.

Card Sorting. We converted the responses from the card sorting exercise into ranks from 0-2, where a score of 0 was given to cards that were placed in the “not very important to health” pile or the “dislike” pile, and a score of 2 was given to the cards that were in the “very important to health” pile or “like a lot” pile, for their respective parameters. Scores for liking were reverse coded so that higher values indicated a dislike. We analyzed the card sorting data using an ANOVA across the three participant groups. We conducted post-hoc analyses to determine homogenous subsets when omnibus differences were detected. There we no missing responses. All analysis were done in SPSS v.25.

Focus Group Discussions. We uploaded transcripts and the codebook into NVivo v.12 for analysis (Nvivo, 2015). We developed a draft codebook then applied thematic analysis to characterize and subsequently modify emergent themes through an iterative approach that combined data collection and analysis concurrently.[52] This allowed us to determine when saturation occurred, when no new themes emerged from the data.

Research Team and Reflexivity

Of the four authors, one is from Angul, India, one is from Nepal, and two are from the United States. Their backgrounds vary from overseeing data collection efforts to social norms/gender norms, and global behavioral health. Each researcher holds a master’s and/or doctoral degree and their familiarity with anemia in India range from

For peer review only - http://bmjopen.bmj.com/site/about/guidelines.xhtml

academic research to personal experience. Three of the authors, SP, ES, and RR, were present for the data collection training and piloting. One of the authors, SP, was present for all data collection. None of the authors have any personal relationship to the participants or any direct benefit from examining the relationship between anemia and fatigue.

Results

Sample

The demographic distribution of each subgroup can be found in Table 1. The average age of men in the sample was 35 years (*SD* = 6.00), and that of women was 28 years (*SD* = 4.76). The average age of mothers-in-law was 56 years (*SD* = 7.58). The majority of women of reproductive age had completed at least secondary school, while the majority of men had not completed secondary school. The majority of mothers-in-law had not received any education. Additionally, the majority of participants had at least two children. All but one participant was also married; the singular participant who was unmarried was widowed. All participants were Hindu. Almost all of the participants belonged to a scheduled caste, tribe, or "other backwards caste."

While the majority of women of reproductive age had taken iron folic acid in the past (86.7%), very few were taking it at the time of the survey (10%). Only about a quarter of mothers-in-law had ever taken iron folic acid, but 9.1% were taking it at the time of the survey. None of the men surveyed reported currently taking iron folic acid, and only 13.3% had ever taken it. About half of women of reproductive age had ever

For peer review only

received an anemia diagnosis, and 14.3 reported currently being anemic at the time of the survey. The demographic distribution of participants who took part in the focus group discussion was very similar to that of the participants who took part in the perceptual mapping activity.[11]

Perceptions of Fatigue

Perceptual maps. The perceptual maps for women of reproductive age show a conceptual separation, as evidenced by their spatial distance, between fatigue and the practical iron folic acid distribution channels for non-pregnant women; one quadrant of the map contains fatigue, while a separate one contains the items related to the iron supplement distribution channels for non-pregnant women, including physicians, clinics, and pharmaceutical kiosks (Figure 1). These three items are clustered together in one quadrant, indicating that they are perceived as similar to each other. Iron folic acid is also plotted in the same quadrant as this cluster, indicating that women of reproductive age may conceptualize physicians, clinics, and pharmaceutical kiosks as links to iron supplements. The separation of fatigue from these iron supplement distribution channels indicates that fatigue may not act as a cue to obtain iron supplements.

Card Sorting Exercise. Confirming these findings, the card sorting exercise showed that women of reproductive age were less likely to report fatigue as important to health than the items related iron supplement distribution channels for non-pregnant women (Table 2); fatigue was considered to be only somewhat important to health by women (M = 0.77, SD = 0.73), while the iron supplement channels, including physicians (M = 1.80, SD = 0.55), clinics (M = 1.80, SD = 0.48), and pharmaceutical kiosks (M = 1.77, SD = 0.50), were considered very important to health.

For peer review only - http://bmjopen.bmj.com/site/about/guidelines.xhtml

Differences in the conceptualization of fatigue and the items related to iron supplement distribution channels for non-pregnant women may also be explained by differences in affective ratings (Table 2). Fatigue was reported to be disliked by women (M = 1.47, SD = 0.68), while the other iron folic acid channel-related items were hardly disliked at all (physician: M = 0.20, SD = 0.41; clinics: M = 0.23, SD = 0.50; pharmaceutical kiosks: M = 0.27, SD = 0.58).

Focus Group Discussions. Women reported that fatigue, and oftentimes, extreme fatigue, was a normal part of daily life. They accepted fatigue as part of their role as wives and mothers; they take care of all of the household responsibilities including all care for the children, serve their husband and in-laws, and often work outside of the home to earn extra money for the family. One woman said, *"We have to work all the time, go to the mountains and fields, have children to take care of. We get no time to rest. We work in unhappiness and eat in pain"* (woman of reproductive age). Women did not think of fatigue as something that they could change and they did not associate their own fatigue with the possibility of having anemia. Although most women knew that fatigue is a symptom of anemia, they underestimated the prevalence of anemia in their village despite endemic rates. A frontline health worker said, *"In our village, in a year, 2-3-4 women have it [anemia]...mainly pregnant women"* (frontline health worker). This is, of course, a far cry from what the actual numbers would be, based on data from the National Family Health Survey,[20] which shows that over half of women of reproductive age are anemic.

Perceptions of Fatigue across Referent Groups

Perceptual maps. Men (Figure 2) and mothers-in-law (Figure 3) also perceived fatigue as different from the other items related to the iron supplement distribution

For peer review only - http://bmjopen.bmj.com/site/about/guidelines.xhtml

channels for non-pregnant women. The perceptual map generated according to men's responses displays physicians, pregnancy, and prenatal care as plotted closely together in the same quadrant. This cluster also contains iron folic acid, indicating that men understand these items and iron supplements as existing in the same cognitive space. Similarly, the perceptual map generated for mothers-in-law displays physicians, clinics, and pharmaceutical kiosks as a cluster plotted near the plot-point for iron folic acid. Fatigue, however, is plotted in a separate quadrant from these items related to the iron supplement distribution channels for non-pregnant women. The separation of fatigue from these channels indicates that fatigue may not act as a cue to obtain iron supplements among husbands and mothers-in-law.

Card Sorting Exercise. We also explored differences in perception across participant groups through the card sorting analysis. Perceptions of importance to health didn't differ across participant groups for any of the anemia-related items shown, with the exception of fatigue ($F = 9.66$, $p < 0.001$) and stomachache ($F = 5.76$, $p < 0.01$), which were significantly different between women of reproductive age and their referent groups. Affective perceptions (i.e. dislike) related to fatigue were also statistically different across the three groups as well ($F = 8.29$, $p < 0.001$).

Post-hoc Least Significant Differences (LSD) tests revealed that women and men had similar perceptions of fatigue across these cognitive and affective dimensions (i.e., importance to health and dislike), but mothers-in-law had significantly different perceptions. Fatigue was perceived with the same level of importance for health by both men ($M = 0.80$, $SD = 0.61$) and women ($M = 0.80$, $SD = 0.61$). However, mothers-in-law had significantly lower perceptions about the importance of fatigue ($M = 0.17$, $SD = 0.53$)

For peer review only - http://bmjopen.bmj.com/site/about/guidelines.xhtml

than men ($p < 0.001$) and women ($p < 0.001$). Additionally, men reported a similar dislike of fatigue as women ($M = 1.23$, $SD = 0.73$, n.s.) while mothers-in-law disliked fatigue to a much greater extent ($M = 1.87$, $SD = 0.35$, $p < 0.05$).

Focus Group Discussions. Men acknowledged that women bear the brunt of the work at home and often face extreme fatigue but did not make the connection that anemia could be at play. A husband from a focus group said, *"They [women] don't have time to do all these works. To do the outside works after completing the household works, they don't get enough time"* (husband). Mothers-in-law felt that their daughters-in-law had it "easier" today than when they were young and showed less empathy than men for their daughters-in-law heavy workload. They also mentioned that younger women today seek medical care more often than their generation. A mother-in-law said, "*We never had those tablets [iron supplements]. We had no idea that we are pregnant until the 5th month but women of today know everything from the 1st month and run to the hospital*" (mother-in-law).

Discussion

This study aimed to understand if the conceptualization of fatigue allowed it to act as a cue for illness in women of reproductive age as well as referent others in a woman's network (i.e., mothers-in-law and husbands). We found that fatigue may not act as a sufficient cue for women to seek treatment for anemia. Nor is fatigue conceptualized in a similar manner across the referent groups of women of reproductive age. These findings were notable in the perceptual maps and card sorting activity, and further manifest through the focus group discussions.

For peer review only - http://bmjopen.bmj.com/site/about/guidelines.xhtml

FATIGUE AS A CUE TO SEEK TREATMENT

The perceptual mapping activity showed a distinction between fatigue and the cluster of items related to the iron supplement distribution channels, which included physicians, clinics, and pharmaceutical kiosks. These clinical items represent necessary steps in the iron folic acid acquisition process for non-pregnant women; the disassociation between fatigue and these clinical items indicates the conceptual distinction between fatigue and a need for medical care. Additionally, the card sorting exercise showed cognitive and affective gaps in perceptions between fatigue, on the one hand, and the aforementioned clinical items, on the other. In particular, women did not perceive fatigue as an important consideration for their health, although they reported dislike for it. These findings are in line with previous literature that discusses the normalization of fatigue as an outcome of hard work rather than an illness.[29]

The qualitative results support the lack of association between fatigue and medical care in the perceptual mapping and card sorting activity. The focus group discussions clarify these findings through the identification of gender norms that may set the conditions for what mental and physical phenomenon are perceived as illness.[11-14, 36] Women in the focus group discussions highlight a prevailing gender norm that women are expected to maintain a large amount of the household duties through the exertion of physical labor, which in turn, can lead to the expectation of fatigue.[29] Women in the focus groups also indicated that they prioritize the wellbeing of their family over their own. Thus, they accept the fatigue that they feel as a normal outcome of their household role. These unequal gender norms that place the majority of household duties on the woman may normalize fatigue in women and negatively affect their risk

For peer review only - http://bmjopen.bmj.com/site/about/guidelines.xhtml

perception. Thus, fatigue is not an adequate cue that illness is present, and consequently, does not act as a cue to seek treatment.

This study also aimed to investigate conceptualizations of fatigue in mothers-in-law and men. The perceptual maps created for mothers-in-law showed a slightly larger dissociation between fatigue and the clinical items related to ion folic acid supplementation than was observed in women of reproductive age. While perceptual mapping methods do not allow for the determination of statistically significant differences, the results of the focus group discussion show that the older generation of women believe that younger women have more sensitive perceptions of health. The mothers-in-law in this study harbored attitudes that were even more dismissive of fatigue than men or younger women; they did not consider fatigue important to health. These results indicate that the normalization of symptoms, like fatigue, may be stronger among older generations of women.

We found a different conceptualization of fatigue in men. The card sorting activity revealed a smaller gap in cognitive and affective perceptions of fatigue; while men may consider fatigue a low health concern, they also do not mind it very much. The results of the focus group discussions offer the explanation that men may not mind fatigue as much as the older generations of women because they are subject to less expectations of labor. The men in the focus group discussion show a sympathy for the workload of women, indicating that they, as men, have a lighter workload. The results of the qualitative investigations coupled with the quantitative findings indicates that fatigue is normalized to a lesser extent in men than it is in women.

For peer review only - http://bmjopen.bmj.com/site/about/guidelines.xhtml

FATIGUE AS A CUE TO SEEK TREATMENT

The conceptualization of fatigue as illness is critical for non-pregnant, out-of-school women who are currently not given iron supplements as part of regular care by front line health workers. If women do not perceive illness when fatigue is present, they may not seek treatment for it (including asking for iron supplements) unless they are pregnant. This may exacerbate an existing gap in iron supplement distribution to non-pregnant, out-of-school women, who make up the largest proportion of women with anemia.[20] Future anemia reduction interventions should take into account the social and cultural underpinnings related to perceptions of illness among women.

Studies show that mothers-in-law are involved in decision-making processes among anemic women in India.[39, 53-54] If mothers-in-law continue to believe and perpetuate the idea that fatigue is not a symptom of illness, women of reproductive age may also follow suit. Even in situations where the women of reproductive age does not agree with her mother-in-law, it may be difficult for her to obtain iron supplements against her mother in laws requests.[55] Therefore, in line with the recommendations of other studies, we highlight the importance of shifting mothers-in-law's perceptions of fatigue and anemia, so that perceptions can shift in women of reproductive age.[39] Additionally, it is imperative that the mother-in-law does not act as a barrier for iron supplementation when a woman of reproductive age recognizes her fatigue as a symptom of anemia.

To our knowledge, this is the first study that explores the gaps in iron folic acid distribution for non-pregnant women through an examination of the social context of Indian women living in Odisha. The current available health services require that non-pregnant, out-of-school, women seek treatment for their anemia, rather than receive iron

For peer review only - http://bmjopen.bmj.com/site/about/guidelines.xhtml

supplements unprompted. However, the most recognizable symptom of anemia, fatigue, is not a cue for illness, and thus, does not act as a cue to seek treatment. This study shows that fatigue is not an adequate cue to seek treatment among non-pregnant women in Odisha, India, resulting in a failure to treat their anemia. We recommend improving the available services to non-pregnant women so that they can obtain iron folic acid through frontline workers. However, within the current health practice landscape of Odisha, India, we recommend increasing knowledge around the connection between fatigue and anemia among mothers-in-laws, husbands, and women of reproductive age to improve iron supplementation use among women. Efforts that promote perceptions of risk for anemia can also contribute on this front. Future studies should measure iron supplement acquisition and adherence in non-pregnant women.

Limitations. Findings from this study provide important contributions to the anemia-reduction literature. However, there are several limitations in this study that must be acknowledged. First, the quantitative methods used in this analysis leave gaps in interpretation. Perceptual maps are a useful tool for depicting the relative conceptualization of items and concepts, but it gives little explanation for the dimension of perception that are depicted. We included a card sorting analysis to attempt to understand the cognitive and affective dimensions. However, there is no method to triangulate these two quantitative methods to understand if the maps are created on these two dimensions. For this reason, we have included a qualitative component, which we have triangulated with the results observed in the quantitative component. We have also used the qualitative component to provide context to the results of the quantitative assessment, despite the fact that they come from different samples. The use of different

For peer review only - http://bmjopen.bmj.com/site/about/guidelines.xhtml

samples to triangulate may pose as a limitation, but it is worthwhile to note both components share a common sampling procedure and sampling frame.

Second, we note that there are limitations in the way that fatigue can be depicted through our quantitative methods. Participants of the perceptual mapping and card sorting activity were shown a picture of a woman experiencing fatigue, followed by a depiction of the word in the local language. It is possible that the term was too technical to be understood properly. However, the face validity of the cards were evaluated by co-investigators in the field minimize the chances of misinterpretation. We also note that the broader goal of the qualitative component was to understand key barriers and facilitators of iron supplementation. As a result, participants were not explicitly asked about their conceptualizations of fatigue. Hence, it is possible that their responses may shift if asked about it directly. Despite this, respondents still revealed a normalization of hard labor among women that corresponds to normalizations of fatigue in the literature.[29]

Furthermore, the four rural communities we studied in Angul, Odisha may not be representative of rural India as a whole, or even some urban areas of Angul. However, our use of random sampling for both the focus groups and the perceptual mapping provides a more representative sample and therefore, may make the findings more generalizable.

For peer review only - http://bmjopen.bmj.com/site/about/guidelines.xhtml

Funding: This study was funded by the Bill and Melinda Gates Foundation under Grant OPP1182519. The funders had no role in study design, data collection and analysis, decision to publish, or preparation of the manuscript.

Author Contributions: HY, ES, and RR made substantial contributions to the conception or design of the work, the analysis, and the interpretation of the work. SP also contributed to the design of the study, and led the data acquisition of data with help from ES and RR. All authors were involved in drafting the work or revising it critically and the final approval of the version to be published. All authors are accountable for all aspects of the work.

Declaration Statement: The authors of this paper have no conflicts of interest to report.

Ethics statement: This study was reviewed and approved by the George Washington University Institutional Review Board (IRB) as well as D-COR Consulting Institutional Ethics Committee (IEC). Data collectors obtained informed consent from all participants in the study.

Data Availability: The data associated with this study can be made available upon request. Anyone who wishes to have access to the de-identified data from the perceptual mapping activity, card sorting activity, or focus group discussions can email the study data manager, Hagere Yilma (corresponding author), at hyilma@gwu.edu. All data is de-identified. Investigators are permitted to access the data for any research questions that have yet to be explored by the study team.

For peer review only

References

1. Benoist, B. d., McLean, E., Egll, I., & Cogswell, M. (2008). Worldwide prevalence of anaemia 1993-2005: WHO global database on anaemia. *Worldwide Prevalence of Anaemia 1993-2005: WHO Global Database on Anaemia.*
2. Strauss, W. E., & Auerbach, M. (2018). Health-related quality of life in patients with iron deficiency anemia: Impact of treatment with intravenous iron. *Patient Related Outcome Measures, 9*, 285.
3. Wouters, H. J., van der Klauw, Melanie M, de Witte, T., Stauder, R., Swinkels, D. W., Wolffenbuttel, B. H., et al. (2019). Association of anemia with health-related quality of life and survival: A large population-based cohort study.*Haematologica, 104*(3), 468-476.
4. Gardner, G. W., Edgerton, V. R., Senewiratne, B., Barnard, R. J., & Ohira, Y. (1977). Physical work capacity and metabolic stress in subjects with iron deficiency anemia. *The American Journal of Clinical Nutrition, 30*(6), 910-917.
5. Allen, L. H. (2000). Anemia and iron deficiency: Effects on pregnancy outcome. *The American Journal of Clinical Nutrition, 71*(5), 1284S.
6. Brabin, B. J., Hakimi, M., & Pelletier, D. (2001). An analysis of anemia and pregnancy-related maternal mortality. *The Journal of Nutrition, 131*(2), 615S.
7. Beard, J. L. (2000). Iron requirements in adolescent females. *The Journal of Nutrition, 130*(2), 442S.
8. Cooke, A. G., McCavit, T. L., Buchanan, G. R., & Powers, J. M. (2017). Iron deficiency anemia in adolescents who present with heavy menstrual bleeding. *Journal of Pediatric and Adolescent Gynecology, 30*(2), 247-250.

For peer review only - http://bmjopen.bmj.com/site/about/guidelines.xhtml

9. Hallberg, L., Högdahl, A., Nilsson, L., & Rybo, G. (1966). Menstrual blood loss and iron deficiency. *Acta Medica Scandinavica, 180*(5), 639-650.

10. Milman, N. (2011). Postpartum anemia I: Definition, prevalence, causes, and consequences. *Annals of Hematology, 90*(11), 1247.

11. XXX (2020). Moving beyond individual barriers and identifying multi-level strategies to reduce anemia in Odisha India. *BMC Public Health, 20*, 1-16.

12. Aurino, E. (2017). Do boys eat better than girls in India? Longitudinal evidence on dietary diversity and food consumption disparities among children and adolescents. *Economics & Human Biology, 25*, 99-111.

13. Bedwell, R. M., Spielvogel, H., Bellido, D., & Vitzthum, V. J. (2017). Factors influencing the use of biomedical health care by rural bolivian anemic women: Structural barriers, reproductive status, gender roles, and concepts of anemia. PloS One, 12(1), e0170475.

14. Jose, S. (2011). Adult undernutrition in India: Is there a huge gender gap? *Economic and Political Weekly*, 95-102.

15. ALIGN. Advancing Learning and Innovation on Gender Norms. (2019). https://www.alignplatform.org/FAQ. Accessed May 26th, 2020.

16. World Health Organization. (2014). *Global nutrition targets 2025: Anaemia policy brief*. Geneva, Switzerland: World Health Organization.

17. Ahmed, F., Khan, M. R., Akhtaruzzaman, M., Karim, R., Williams, G., Torlesse, H., et al. (2010). Long-term intermittent multiple micronutrient supplementation enhances hemoglobin and micronutrient status more than iron folic acid

For peer review only - http://bmjopen.bmj.com/site/about/guidelines.xhtml

supplementation in bangladeshi rural adolescent girls with nutritional anemia. *The Journal of Nutrition, 140*(10), 1879-1886.

18. Vir, S. C., Singh, N., Nigam, A. K., & Jain, R. (2008). Weekly iron and folic acid supplementation with counseling reduces anemia in adolescent girls: A large-scale effectiveness study in uttar pradesh, india. *Food and Nutrition Bulletin, 29*(3), 186-194.

19. World Health Organization. (2016). *Guideline: Daily iron supplementation in adult women and adolescent girls*. Geneva, Switzerland: World Health Organization.

20. International Institute for Population Sciences. (2017). *National family health survey (NFHS-4), 2015-16*. India. Mumbai: IIPS.

21. Bellad, A., Kapil, R., & Gupta, A. (2018). National guidelines for prevention and control of iron deficiency anemia in india. *Indian Journal of Community Health, 30*(01), 89-94.

22. Kumar, A. (1999). National nutritional anaemia control programme in India. *Indian Journal of Public Health, 43*(1), 3-5.

23. Kapil, U., & Bhadoria, A. S. (2014). National iron-plus initiative guidelines for control of iron deficiency anaemia in India, 2013. *Natl Med J India, 27*(1), 27-29.

24. Nguyen, P. H., Scott, S., Avula, R., Tran, L. M., & Menon, P. (2018). Trends and drivers of change in the prevalence of anaemia among 1 million women and children in India, 2006 to 2016. *BMJ Global Health, 3*(5), e001010.

25. Rai, R. K., Fawzi, W. W., Barik, A., & Chowdhury, A. (2018). The burden of iron-deficiency anaemia among women in India: How have iron and folic acid interventions fared? *WHO South-East Asia Journal of Public Health, 7*(1), 18.

For peer review only - http://bmjopen.bmj.com/site/about/guidelines.xhtml

26. UNICEF, & AIIMS. (2018). *Process documentation of national iron plus initiative (NIPI) – towards an anaemia free Odisha, 2016-17*. Bhubaneswar, Odisha: UNICEF.

27. Bager, P. (2014). Fatigue and acute/chronic anaemia. *Dan Med J*, *61*(4), B4824.

28. Sobrero, A., Puglisi, F., Guglielmi, A., Belvedere, O., Aprile, G., Ramello, M., et al. (2001). Fatigue: A main component of anemia symptomatology. Paper presented at the *Seminars in Oncology, 28.* pp. 15-18.

29. Loriol, M. (2017). A sociological stance on fatigue and tiredness: Social inequalities, norms and representations. *Neurophysiologie Clinique/Clinical Neurophysiology, 47*(2), 87-94.

30. Maaita, M., Irtaimeh, T., Altaleb, N., & Ibrahim, R. (2018). Perception of iron deficiency anemia (IDA) and compliance of iron supplements among pregnant women admitted to the labor ward at King Hussein Medical Center, Amman, Jordan. *JRMS, 25*(2), 12-18.

31. Shankar, M., & Reddy, B. (2012). Anaemia in pregnancy still a major cause of morbidity and mortality: insights from Koppal district, Karnataka, India. *Reproductive health matters, 20*(40).

32. Vincent, A., Keerthana, K., Damotharan, K., Newtonraj, A., Bazroy, J., & Manikandan, M. (2017). Health care seeking behaviour of women during pregnancy in rural south India: A qualitative study. *International Journal of Community Medicine and Public Health, 4*(10), 3636-3639.

33. Ball, H. A., Siribaddana, S. H., Sumathipala, A., Kovas, Y., Glozier, N., Rijsdijk, F., ... & Hotopf, M. (2011). Genetic and environmental contributions to the overlap

For peer review only - http://bmjopen.bmj.com/site/about/guidelines.xhtml

between psychological, fatigue and somatic symptoms: a twin study in Sri Lanka. *Twin Research and Human Genetics, 14*(1), 53-63.

34. Ray, C., Weir, W. R. C., Cullen, S., & Phillips, S. (1992). Illness perception and symptom components in chronic fatigue syndrome. *Journal of Psychosomatic Research, 36*(3), 243-256.

35. Bensing, J. M., Hulsman, R. L., & Schreurs, K. M. (1999). Gender differences in fatigue: biopsychosocial factors relating to fatigue in men and women*. Medical care, 37(*10), 1078-1083.

36. Diamond-Smith, N. G., Gupta, M., Kaur, M., & Kumar, R. (2016). Determinants of persistent anemia in poor, urban pregnant women of Chandigarh city, North India: a mixed method approach. Food and nutrition bulletin, 37(2), 132-143.

37. Seymour, S. C. (2010). Environmental change, family adaptations, and child development: Longitudinal research in India. Journal of cross-cultural psychology, 41(4), 578-591.

38. Krupp, K., Placek, C. D., Wilcox, M., Ravi, K., Srinivas, V., Arun, A., & Madhivanan, P. (2018). Financial decision making power is associated with moderate to severe anemia: A prospective cohort study among pregnant women in rural South India. *Midwifery*, *61*, 15-21.

39. Varghese, R., & Roy, M. (2019). Coresidence with mother-in-law and maternal anemia in rural India. *Social Science & Medicine, 226*, 37-46.

40. Adaval, R., Coupey, E., & Narayanan, S. (2015). Direct mapping of consumer perceptions. Paper presented at the *Proceedings of the 1995 Academy of Marketing Science (AMS) Annual Conference,* pp. 255-263.

41. Hastings, G., Angus, K., & Bryant, C. (Eds.). (2011). *The Sage handbook of social marketing*. Sage.

42. Iacobucci, D., Grisaffe, D., & DeSarbo, W. (2017). Statistical perceptual maps: Using confidence region ellipses to enhance the interpretations of brand positions in multidimensional scaling. *Journal of Marketing Analytics, 5*(3-4), 81-98.

43. Bass, S. B., Jessop, A., Maurer, L., Gashat, M., Al Hajji, M., & Gutierrez, M. (2018). Mapping the barriers and facilitators of HCV treatment initiation in methadone maintenance therapy patients: Implications for intervention development. *Journal of Health Communication, 23*(1), 117-127.

44. Bass, S. B., Wolak, C., Greener, J., Tedaldi, E., Nanavati, A., Ruppert, K., et al. (2016). Using perceptual mapping methods to understand gender differences in perceived barriers and benefits of clinical research participation in urban minority HIV patients. *AIDS Care, 28*(4), 528-536.

45. Pivonka, E., Seymour, J., McKenna, J., Baxter, S. D., & Williams, S. (2011). Development of the behaviorally focused Fruits & Veggies—More Matters public health initiative. Journal of the American Dietetic Association, 111(10), 1570-1577.

46. Ruggieri, D. G., Bass, S. B., Alhajji, M., & Gordon, T. F. (2018). Understanding parents' perceptions of school-based BMI screening and BMI report cards using perceptual mapping: Implications for school nurses. *The Journal of School Nursing*, 1059840518789243.

47. Van Voorhees, B. W., Watson, N., Bridges, J. F., Fogel, J., Galas, J., Kramer, C., ... & Landsback, J. (2010). Development and pilot study of a marketing strategy for primary care/Internet–based depression prevention intervention for adolescents (The

CATCH-IT Intervention). Primary care companion to the Journal of clinical psychiatry, 12(3).

48. XXX (2018). The RANI Project: A socio-normative intervention to reduce anemia in Odisha, India: A formative research protocol. *Gates open research*, 2.

49. Nguyen, P. H., Sanghvi, T., Kim, S. S., Tran, L. M., Afsana, K., Mahmud, Z., et al. (2017). Factors influencing maternal nutrition practices in a large scale maternal, newborn and child health program in Bangladesh. *PLoS One, 12*(7), e0179873.

50. Tinago, C. B., Annang Ingram, L., Blake, C. E., & Frongillo, E. A. (2017). Individual and structural environmental influences on utilization of iron and folic acid supplementation among pregnant women in Harare, Zimbabwe. *Maternal & child nutrition*, 13(3), e12350.

51. Bijmolt, T. H., & Wedel, M. (1999). A comparison of multidimensional scaling methods for perceptual mapping. *Journal of Marketing Research, 36*(2), 277-285.

52. Guest, G., MacQueen, K. M., & Namey, E. E. (2011). *Applied thematic analysis*. Sage Publications.

53. Das, A., & Sarkar, M. (2014). Pregnancy-related health information-seeking behaviors among rural pregnant women in india: Validating the wilson model in the indian context. *The Yale Journal of Biology and Medicine, 87*(3), 251.

54. Ramachandran, D., Canny, J., Das, P. D., & Cutrell, E. (2010). Mobile-izing health workers in rural india. Paper presented at the *Proceedings of the SIGCHI Conference on Human Factors in Computing Systems,* pp. 1889-1898.

55. Allendorf, K. (2013). Going nuclear? family structure and young women's health in india, 1992–2006. *Demography, 50*(3), 853-880.

For peer review only - http://bmjopen.bmj.com/site/about/guidelines.xhtml

Table 1. Demographic Information- Perceptual Mapping and Card Sorting

	Men (n=30)	WRA (n=30)	MILs (n=30)
	M (SD)	*M (SD)*	*M (SD)*
**Age**	34.67 (6.00)	27.90 (4.76)	55.87 (7.58)
	**n (%)**	**n (%)**	**n (%)**
**School**			
None	3 (10)	4 (13.3)	21 (70.0)
Up to Primary	12 (40)	8 (26.7)	6 (20.0)
Up to Secondary	6 (20)	7 (23.3)	2 (6.7)
Up to High Secondary	5 (16.6)	10 (33.4)	1 (3.3)
Up to Tertiary	4 (13.3)	1 (3.3)	0 (0)
**Married**	30 (100)	30 (100)	29 (96.7)[a]
**Religion-Hindu**	100 (100)	100 (100)	100 (100)
**Caste**			
Scheduled Caste/ Tribe	18 (60.0)	16 (53.3)	17 (56.6)
Other Backwards Caste	11 (36.7)	12 (40.0)	13 (43.3)
Other Caste	1 (3.3)	2 (6.7)	0 (0)
**Children**			
None	0 (0)	3 (10)	0 (0)
1	4 (13.3)	7 (23.3)	3 (10.0)
2	12 (40.0)	10 (33.3)	3 (10.0)

For peer review only - http://bmjopen.bmj.com/site/about/guidelines.xhtml

FATIGUE AS A CUE TO SEEK TREATMENT

	Men	WRA	MILs
3	10 (33.3)	7 (23.3)	11 (36.7)
4 or more	4 (13.3)	3 (10.0)	13 (43.3)
**Ever taken IFA**	4 (13.3)	26 (86.7)	7 (23.3)
**Currently taking IFA**	0 (0)	3 (10.0)	2 (9.1)
**Diagnoses of anemia ever**	1 (3.3)	16 (53.3)	4 (13.3)
**Currently anemic?**	5 (21.7)	4 (14.3)	3 (13.0)

Note: Under “School”, “I” indicates incomplete and “C” indicates complete; [a] The one mother-in-law who wasn’t married was a widow. WRA = women of reproductive age; MILs = Mothers-in-law; IFA = iron folic acid.

*p < 0.05, **p < 0.01, ***p < 0.001

For peer review only - http://bmjopen.bmj.com/site/about/guidelines.xhtml

Table 2. Importance to Health and Dislike Rating Across Participant Category

		WRA	Men	MILs	
Item	Rating Parameter	*M* (SD)	*M* (SD)	*M* (SD)	*F*
IFA pill	Importance	1.67 (0.68)	1.87 (0.35)	1.73 (0.52)	2.39
	Dislike	0.53 (0.73)	0.20 (0.48)	0.47 (0.73)	2.15
Clinic	Importance	1.80 (0.48)	1.93 (0.25)	1.83 (0.38)	0.98
	Dislike	0.23 (0.50)	0.20 (0.41)	0.23 (0.50)	0.05
Kiosk	Importance	1.77 (0.50)	1.93 (0.25)	1.87 (0.35)	1.44
	Dislike	0.27 (0.58)	0.17 (0.38)	0.13 (0.43)	0.64
Self-Help Groups	Importance	1.37 (0.72)	1.27 (0.74)	1.57 (0.68)	1.38
	Dislike	0.30 (0.47)[a]	0.73 (0.74)[b]	0.50 (0.68)[a,b]	3.44*
Traditional healer	Importance	0.80 (0.76)	1.23 (0.82)	1.03 (0.67)	2.50
	Dislike	1.07 (0.78)	0.97 (0.85)	1.07 (0.69)	0.17
Physician	Importance	1.80 (0.55)	2.00 (0)	1.90 (0.31)	2.27
	Dislike	0.20 (0.41)	0.10 (0.31)	0.20 (0.48)	0.61
Fatigue	Importance	0.77 (0.73)[a]	0.80 (0.61)[a]	0.17 (0.53)[b]	9.66***
	Dislike	1.47 (0.68)[a]	1.23 (0.73)[a]	1.87 (0.35)[b]	8.29***
Prenatal care	Importance	1.43 (0.73)	1.40 (0.67)	1.67 (0.61)	1.40
	Dislike	0.70 (0.89)	0.57 (0.50)	0.33 (0.66)	2.12
Green veggies	Importance	1.63 (0.62)	1.63 (0.49)	1.80 (0.41)	1.06
	Dislike	0.37 (0.61)	0.30 (0.60)	0.13 (0.35)	1.52
Meat	Importance	1.23 (0.68)	0.97 (0.76)	1.37 (0.61)	2.62
	Dislike	0.77 (0.73)	0.90 (0.76)	0.60 (0.62)	1.36

For peer review only - http://bmjopen.bmj.com/site/about/guidelines.xhtml

Pregnancy	Importance	1.13 (0.82)	1.23 (0.63)	1.57 (0.68)	3.04
	Dislike	0.80 (0.85)[a]	0.80 (0.48)[a]	0.30 (0.60)[b]	5.74**
Stomach-ache	Importance	0.63 (0.67)[a]	0.87 (0.68)[a]	0.30 (0.60)[b]	5.76**
	Dislike	1.47 (0.68)[a]	1.17 (0.65)[b]	1.83 (0.46)[c]	9.15***

Note: The letters a and b denote homogenous subsets yielded from the post-hoc analysis when omnibus significance was detected. WRA = women of reproductive age; MILs = Mothers-in-law; IFA = iron folic acid.

*p < 0.05, **p < 0.01, ***p < 0.001

For peer review only

Figure Captions.

Figure 1. Perceptual Map derived for Women of Reproductive Age. Dissimilarity plot across two-dimensions (corresponding to the x and y axis) as displayed by SPSS v. 25; plot point labels have been edited for reader clarity.

Figure 2. Perceptual Map derived for Men. Dissimilarity plot across two-dimensions (corresponding to the x and y axis) as displayed by SPSS v. 25; plot point labels have been edited for reader clarity.

Figure 3. Perceptual Map derived for Mothers-in-Law. Dissimilarity plot across two-dimensions (corresponding to the x and y axis) as displayed by SPSS v. 25; plot point labels have been edited for reader clarity.

For peer review only

For peer review only - http://bmjopen.bmj.com/site/about/guidelines.xhtml

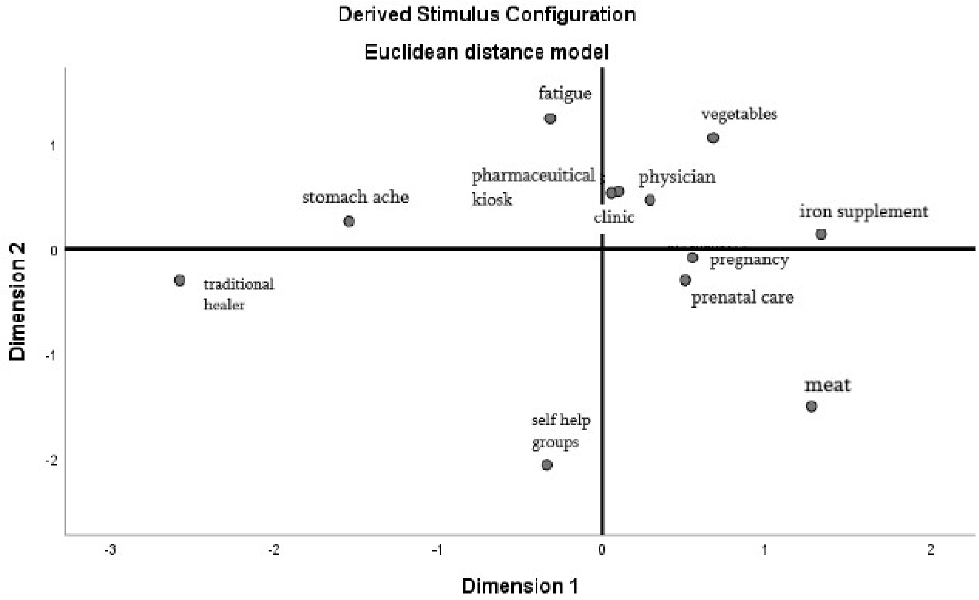

Figure 1. Perceptual Map derived for Women of Reproductive Age. Dissimilarity plot across two-dimensions (corresponding to the x and y axis) as displayed by SPSS v. 25; plot point labels have been edited for reader clarity.

171x107mm (96 x 96 DPI)

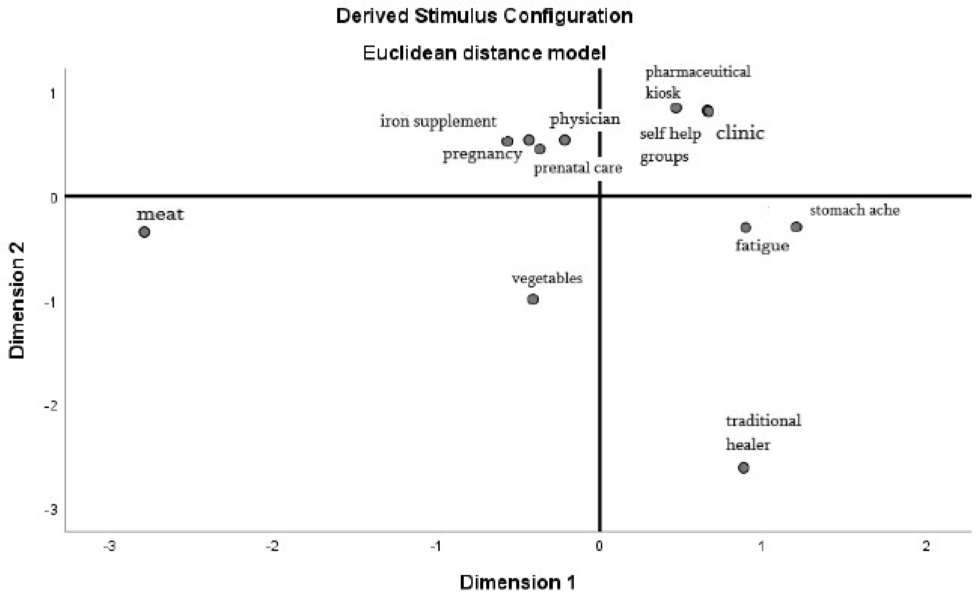

Figure 2. Perceptual Map derived for Males. Dissimilarity plot across two-dimensions (corresponding to the x and y axis) as displayed by SPSS v. 25; plot point labels have been edited for reader clarity.

175x109mm (96 x 96 DPI)

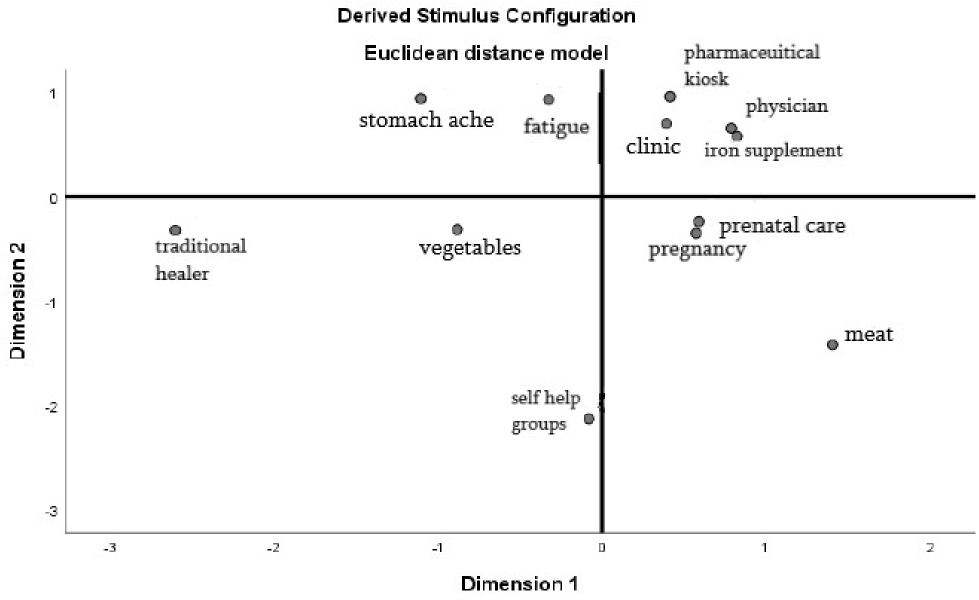

Figure 2. Perceptual Map derived for Males. Dissimilarity plot across two-dimensions (corresponding to the x and y axis) as displayed by SPSS v. 25; plot point labels have been edited for reader clarity.
Figure 3. Perceptual Map derived for Mothers-in-Law. Dissimilarity plot across two-dimensions (corresponding to the x and y axis) as displayed by SPSS v. 25; plot point labels have been edited for reader clarity.

177x111mm (96 x 96 DPI)

Supplement 1

PERCEPTUAL MAPPING AND CARD SORTING QUESTIONNAIRE

FOR RESEARCH TEAM MEMBER USE ONLY/ କେବଳ ଅନୁସନ୍ଧାନକାରୀ ଦଳର ସଦସ୍ୟଙ୍କ ବ୍ୟବହାର ନିମନ୍ତେ:		
BLOCK	Which Block is the respondent from/ଉତ୍ତରଦାତା କେଉଁ ବ୍ଲକରୁ?	....
VILLAGE	Which village is the respondent from/ଉତ୍ତରଦାତା କେଉଁ ଗ୍ରାମରୁ?	
PARTICIPATION	The respondent is participating in a...<br>ଉତ୍ତରଦାତାଜଣକ କେଉଁଥିରେ ଭାଗ ନେଉଛନ୍ତି	Focus group discussion.......................1<br>ଦଳଗତ ଆଲୋଚନା...1<br>Key informant interview........................2<br>ପ୍ରମୁଖ ସୂଚନାଦାତାଙ୍କ ସହିତ ସାକ୍ଷାତକାର ....2<br>Perceptual Mapping -------------------3<br>ଧାରଣାତ୍ମକ ମାନଚିତ୍ରଣ --------------------------3
CODE_PM	What is the code number of the Perceptual Mapping?<br>ଏହି ଧାରଣାତ୍ମକ ମାନଚିତ୍ରଣର କୋଡ କଣ?	Code number/ କୋଡ ନମ୍ବର୍ __________<br>PM1<br>PM2<br>PM3<br>PM4<br>PM5<br>PM6<br>PM7<br>PM8<br>PM9

For peer review only - http://bmjopen.bmj.com/site/about/guidelines.xhtml

		PM10<br>PM11<br>PM12
PARTICIPANT_PM	Participant category of Perceptual Mapping ଧାରଣାତ୍ମକ ମାନଚିତ୍ରଣରେ ଅଂଶଗ୍ରହଣ କରିଥିବା ଉତ୍ତରଦାତାଙ୍କ ପ୍ରକାର	Males 18 – 42 years old --------------------1<br>ପୁରୁଷ (୧୮-୪୨) (ବିବାହିତ) ------------------------1<br>WRA 15 – 35 years old ---------------------2<br>ପ୍ରଜନନ ବୟସ (୧୫-୩୫) ମଧ୍ୟରେ ଥିବା ମହିଳା ------2<br>Mothers in law – any age -----------------3<br>ଶାଶୁ (ସ୍ୱାମୀଙ୍କ ମା) ---------------------------------3
CODE_NUMBER_PM	Participant code number of the Perceptual Mapping ଧାରଣାତ୍ମକ ମାନଚିତ୍ରଣରେ ଭାଗ ନେଇଥିବା ସଦସ୍ୟଙ୍କ କୋଡ଼	
PATRICIPATION_DATE	On what date did the respondent participate/କେଉଁ ତାରିଖରେ ଉତ୍ତରଦାତା ଅଂଶଗ୍ରହଣ କରିଥିଲେ?	DD/MM: ________________________<br>ତାରିଖ / ମାସ ______________________

Thank you for participating in this study. I want to remind you that there are no right or wrong answers to these questions. We just want to know what your opinions are. Before we ask for your opinions, please tell us a few things about yourself/ଆପଣ ଏହି ଅଧ୍ୟୟନରେ ଅଂଶଗ୍ରହଣ କରିଥିବାରୁ ଧନ୍ୟବାଦ । ମୁଁ ଆପଣଙ୍କୁ ମନେ ପକେଇଦେବାକୁ ଚାହେଁ ଯେ ଏହି ସବୁ ପ୍ରଶ୍ନର ଠିକ କିମ୍ବା ଭୁଲ ଉତ୍ତର ନାହିଁ । ଆମେ କେବଳ ଆପଣଙ୍କ ମତାମତ ଗୁଡିକ ଜାଣିବାକୁ ଚାହୁଁଛୁ, ଦୟାକରି ଆପଣଙ୍କ ବିଷୟରେ ଆମକୁ କିଛି କୁହନ୍ତୁ ।

Section/ବିଭାଗ 1

Demographic Questionnaire/ଜନସଂଖ୍ୟା ସମ୍ବନ୍ଧୀୟ ପ୍ରଶ୍ନାବଳୀ

1	How old are you?<br>ଆପଣଙ୍କର ବୟସ କେତେ?	Age: ________________ (Unknown = -99)<br>ବୟସ: ------------------------ (ଜାଣି ନାହାନ୍ତି = -୯୯)
2	What is your sex?	Female/ମହିଳା 1

For peer review only - http://bmjopen.bmj.com/site/about/guidelines.xhtml

	ଆପଣଙ୍କର ଲିଙ୍ଗ କଣ?	Male/ପୁରୁଷ....................................2<br>No response/କୌଣସି ଉତ୍ତର ନାହିଁ..._......3
3	What is the highest level of school you have attended?<br>ଆପଣଙ୍କର ସର୍ବୋଚ୍ଚ ଶିକ୍ଷାଗତ ଯୋଗ୍ୟତା କଣ?	None/କିଛିନାହିଁ - 0<br>Primary school (incomplete)/ପ୍ରାଥମିକ ବିଦ୍ୟାଳୟ (ଅସମ୍ପୂର୍ଣ) - 1<br>Primary school (complete) /ପ୍ରାଥମିକ ବିଦ୍ୟାଳୟ (ସମ୍ପୂର୍ଣ) - 2<br>Secondary school (incomplete) /ମାଧ୍ୟମିକ ବିଦ୍ୟାଳୟ (ଅସମ୍ପୂର୍ଣ) - 3<br>Secondary school (complete) /ମାଧ୍ୟମିକ ବିଦ୍ୟାଳୟ (ସମ୍ପୂର୍ଣ) - 4<br>High Secondary/ Senior Secondary school (incomplete) /ଉଚ୍ଚ ମାଧ୍ୟମିକ ବିଦ୍ୟାଳୟ (ଅସମ୍ପୂର୍ଣ) - 5<br>High Secondary/ Senior school (complete) /ଉଚ୍ଚ ମାଧ୍ୟମିକ ବିଦ୍ୟାଳୟ (ସମ୍ପୂର୍ଣ) - 6<br>Tertiary/College/University (incomplete)/ମହାବିଦ୍ୟାଳୟ/ବିଶ୍ୱବିଦ୍ୟାଳୟ (ଅସମ୍ପୂର୍ଣ) - 7<br>Tertiary/College/University (complete)/ମହାବିଦ୍ୟାଳୟ/ବିଶ୍ୱବିଦ୍ୟାଳୟ (ସମ୍ପୂର୍ଣ) - 8<br>Other (Specify)/ଅନ୍ୟାନ୍ୟ (ଦର୍ଶାନ୍ତୁ):- 88<br>No response/କୌଣସି ଉତ୍ତରନାହିଁ...........-99
4	Are you currently married or living together with a partner as if married?<br>ଆପଣ ବିବାହିତ କି ବା ଜଣେ ସାଥିଙ୍କ ସହିତ ବିବାହିତ ଭଳି ମିଶିକରି ରୁହନ୍ତି କି ?	Yes, currently married/ହଁ, ବିବାହିତ...........1<br>Yes, living with a partner/ହଁ, ଜଣେ ସାଥୀଙ୍କ ସହିତ ରହୁଛି ....2<br>No/ନାହିଁ...3<br>No response/ କୌଣସି ଉତ୍ତର ନାହିଁ...........-99
4a	IF NO: Are you divorced, separated, widowed, or never married or in a union?<br>(Skip if married or living with a partner.)	Never married or in a union/କେବେ ବିବାହ କରିନାହାନ୍ତି କିମ୍ବା କୌଣସି ସାଥୀଙ୍କ ସହିତ ରଖୁନାହାଁନ୍ତ...1<br>Divorced/separated/ଛାଡପତ୍ର/ଅଲଗା ରହୁଛନ୍ତି...2<br>Widow/widower / ବିଧବା / ବିପତି.............3

For peer review only - http://bmjopen.bmj.com/site/about/guidelines.xhtml

	ଯଦି ନା : ଆପଣ କଣ ଛାଡପତ୍ର ନେଇଛନ୍ତି, ଅଲଗା ରହୁଛନ୍ତି, ବିଧବା ବା କେବେ ବିବାହ କରିନାହାନ୍ତି କିମ୍ବା କୌଣସି ସାଥୀଙ୍କ ସହିତ ନାହାନ୍ତି ?<br>(ପଚାରନ୍ତୁ ନାହିଁ, ଯଦି ବିବାହିତ କିମ୍ବା ସାଥି ସହିତ ରହୁଛନ୍ତି)	No response/ କୌଣସି ଉତ୍ତର ନାହିଁ.............-99
5	What is your religion?<br>ଆପଣଙ୍କର ଧର୍ମ କଣ ?	Hindu/ହିନ୍ଦୁ ....1<br>Muslim/ମୁସଲିମ ....2<br>Christian/ଖ୍ରୀଷ୍ଟିୟାନ..............................3<br>Sikh/ଶିଖ.......................................4<br>Buddhist/ବୁଦ୍ଧ...................................5<br>Jain/ଜୈନ..6<br>Other/ଅନ୍ୟାନ୍ୟ..................................7<br>No response/ କୌଣସି ଉତ୍ତର ନାହିଁ.........-99
CASTE	What is your caste ?<br>ଆପଣଙ୍କର ଜାତି କଣ ?	Scheduled Caste ଅନୁସୂଚିତ ଜାତି - 1<br>Scheduled Tribe ଅନୁସୂଚିତ ଜନଜାତି - 2<br>OBC ଅନ୍ୟାନ୍ୟ ପଛୁଆ ବର୍ଗର ଜାତି - 3<br>Other Caste ଅନ୍ୟାନ୍ୟ ଜାତି - 4<br>No response/ କୌଣସି ଉତ୍ତର ନାହିଁ.........-99
6	How many children do you have?<br>ଆପଣଙ୍କର କେତୋଟି ସନ୍ତାନ ଅଛନ୍ତି ?	None/କିଛିନାହିଁ....................................0<br>One/ଗୋଟିଏ.....................................1<br>Two/ଦୁଇଟି...2<br>Three/ତିନୋଟି....................................3<br>Four/ଚାରିଟି.......................................4<br>Five or more/ପାଞ୍ଚ କିମ୍ବା ଅଧିକ................5<br>No response// କୌଣସି ଉତ୍ତର ନାହିଁ...........-99
7	How often do you watch television?<br>ଆପଣ କେତେ ବ୍ୟବଧାନରେ ଟିଭି ଦେଖନ୍ତି ?	Not at all/କେବେ ନାହିଁ...............................0<br>Less than once a week/ସପ୍ତାହରେ ଥରକରୁ କମ୍...1<br>At least once a week/ ଅତିକମ୍ ରେ ସପ୍ତାହକୁ ଥରେ 2

For peer review only - http://bmjopen.bmj.com/site/about/guidelines.xhtml

		Almost Every Day/ପ୍ରାୟ ପ୍ରତ୍ୟକ ଦିନ............3
7a	On an average day, for how long do you watch television?<br>ଗୋଟିଏ ଦିନରେ, ପାଖାପାଖ୍ କେତେ ସମୟ ଟିଭି ଦେଖନ୍ତି ?	______Hours per day/ OR ____ minutes per day<br>ଦିନରେ .... ଘଣ୍ଟା କିମ୍ବା ଦିନରେ .... ମିନିଟ୍
8	How often do you listen to the radio?<br>ଆପଣ କେତେଥର ରେଡିଓ ଶୁଣନ୍ତି ?	Not at all/କେବେ ନାହିଁ...............................0<br>Less than once a week/ ସପ୍ତାହରେ ଥରକରୁ କମ୍...1<br>At least once a week/ ଅତିକମ୍ ରେ ସପ୍ତାହକୁ ଥରେ ....2<br>Almost Every Day/ ପ୍ରାୟ ସବୁଦିନ.................3
8a	On an average day, for how long do you listen to the radio?<br>ଗୋଟିଏ ଦିନରେ ପାଖାପାଖ୍ କେତେ ସମୟ ରେଡିଓ ଶୁଣନ୍ତି?	______Hours per day OR ____ minutes per day<br>ଦିନରେ .... ଘଣ୍ଟା କିମ୍ବା ଦିନରେ .... ମିନିଟ
9.0	How often do you talk on a mobile phone?<br>ଆପଣ କେତେଥର ମୋବାଇଲରେ କଥା ହୁଅନ୍ତି?	Not at all/କେବେ ନାହିଁ...............................0<br>Less than once a week/ ସପ୍ତାହରେ ଥରକରୁ କମ୍...1<br>At least once a week/ ଅତିକମ୍ ରେ ସପ୍ତାହକୁ ଥରେ ....2<br>Almost Every Day/ପ୍ରାୟ ସବୁଦିନ.................3
9	Do you use a smart phone?<br>ଆପଣ ସ୍ମାର୍ଟ ଫୋନ୍ ବ୍ୟବହାର କରନ୍ତି କି ?	Yes/ହଁ..1<br>No/ନା..0
9a	On an average day, for how long do you talk on a mobile phone?<br>ଗୋଟିଏ ଦିନରେ, ପାଖାପାଖ୍ କେତେ ସମୟ ମୋବାଇଲରେ କଥା ହୁଅନ୍ତି?	______Hours per day OR ____ minutes per day<br>ଦିନରେ .... ଘଣ୍ଟା କିମ୍ବା ଦିନରେ .... ମିନିଟ
10	How often do you read the newspaper?<br>ଆପଣ କେତେଥର ଖବରକାଗଜ ପଢନ୍ତି?	Not at all/କେବେ ନାହିଁ...............................0<br>Less than once a week/ ସପ୍ତାହରେ ଥରକରୁ କମ୍...1<br>At least once a week/ ଅତିକମ୍ ରେ ସପ୍ତାହକୁ ଥରେ ....2<br>Almost Every Day/ପ୍ରାୟ ସବୁଦିନ.................3

For peer review only

For peer review only - http://bmjopen.bmj.com/site/about/guidelines.xhtml

10a	On an average day, for how long do you read the newspaper?<br>ଗୋଟିଏ ଦିନରେ, ପାଖାପାଖି କେତେ ସମୟ ଖବରକାଗଜ ପଢନ୍ତି?	______Hours per day OR ____ minutes per day<br>ଦିନରେ .... ଘଣ୍ଟା କିମ୍ବା ଦିନରେ .... ମିନିଟ
11	Have you ever attended a self-help group meeting?<br>ଆପଣ କେବେ ସ୍ୱୟଂ ସହାୟକ ଦଳର ବୈଠକରେ ଯୋଗ ଦେଇଛନ୍ତି କି ?	Yes/ହଁ...1<br>No/ନା...0
11a	Are you currently a part of a self-help group/ଆପଣ ବର୍ତ୍ତମାନ ସ୍ୱୟଂ ସହାୟକ ଦଳର ଜଣେ /ସଦସ୍ୟ କି?	Yes/ହଁ...1<br>No/ନା...0
12	Have you ever taken iron & folic acid supplements (IFA)/ଆପଣ କେବେ ବି ଆଇରନ ଏବଂ ଫଲିକ ଏସିଡର ପରିପୂରକ ଖାଇଛନ୍ତି କି ?	Yes/ହଁ...1<br>No/ନା...0
12a	Are you currently taking iron & folic acid supplements (IFA)/ଆପଣ ବର୍ତ୍ତମାନ ଆଇରନ ଏବଂ ଫଲିକ ଏସିଡର ପରିପୂରକ ଖାଉଛନ୍ତି କି ?	Yes/ହଁ...1<br>No/ନା...0
13	Has a doctor or healthcare provider ever told you that you have anemia/ଆପଣଙ୍କର ରକ୍ତହୀନତା ଅଛି ବୋଲି ଆପଣଙ୍କୁ ଡାକ୍ତର କିମ୍ବା କୌଣସି ସ୍ୱାସ୍ଥ୍ୟ ପ୍ରଦାନକାରୀ କେବେ କହିଛନ୍ତି କି ?	Yes/ହଁ...1<br>No/ନା...0
13a	Do you currently have anemia/ବର୍ତ୍ତମାନ ଆପଣଙ୍କର ରକ୍ତହୀନତା ରହିଛି କି ?	Yes/ହଁ...1<br>No/ନା...0

For peer review only - http://bmjopen.bmj.com/site/about/guidelines.xhtml

Section/ବିଭାଗ 2

I am now going to show you two cards at a time. One card has the picture of one object and the second card has the picture of a second object. Please tell me how similar the second card is to the first. To make this a bit easier, I will show you the second card and first ask you whether it is similar or different. If you say “similar,” then I will ask you whether it is somewhat similar or very similar; if you say “different,” then I will ask you whether it is somewhat different or very different.

ଏବେ ମୁଁ ଆପଣଙ୍କୁ ଏକ ସମୟରେ ଦୁଇଟି କାର୍ଡ ଦେଖାଇବାକୁ ଯାଉଛି | ଗୋଟିଏ କାର୍ଡରେ ଗୋଟିଏ ବିଷୟରେ ଛବି ଏବଂ ଦ୍ଵିତୀୟ କାର୍ଡରେ ଦ୍ଵିତୀୟ ବିଷୟରେ ଛବି ରହିଛି । ଦୟାକରି ମୋତେ କୁହନ୍ତୁ ଦ୍ଵିତୀୟ କାର୍ଡଟି ପ୍ରଥମ କାର୍ଡ ସହିତ କେତେ ସମାନ ଅଛି । ଆମେ ଏହାକୁ ଆଉ ଟିକେ ସହଜ କରିବା ପାଇଁ ମୁଁ ଆପଣଙ୍କୁ ଦ୍ଵିତୀୟ କାର୍ଡଟି ଦେଖାଇବି ଏବଂ ପ୍ରଥମେ ପଚାରିବି ଏହା ସମାନ କିମ୍ବା ଅଲଗା । ଯଦି ଆପଣ ସମାନ କହନ୍ତି ତେବେ କିଛି ସମାନ ନା ସମ୍ପୂର୍ଣ ସମାନ ବୋଲି ମୁଁ ପଚାରିବି; ଯଦି ଆପଣ ଅଲଗା କହନ୍ତି ତେବେ କିଛି ଅଲଗା ନା ସମ୍ପୂର୍ଣ ଅଲଗା ବୋଲି ମୁଁ ପଚାରିବି ।

[Show respondent each card pair: 1 versus 2, 3, 4,..... 16; 2 versus 3, 4, 5, ... 16; etc.]

[ଉତ୍ତରଦାତାଙ୍କୁ ପ୍ରତ୍ୟେକ କାର୍ଡର ଯୋଡି ଦେଖାନ୍ତୁ: 1 ସହିତ 2, 3, 4 ... 16; 2 ସହିତ 3, 4, 5, ... 16 ଇତ୍ୟାଦି]

1. IFA Pill/ଆଇରନ ବଟିକା
2. Clinic/କ୍ଲିନିକ
3. Kiosk
4. Self-help group/ସ୍ୱୟଂ ସହାୟକ ଦଳ
5. Traditional healer/ ପାରମ୍ପରିକ / ଚିକିତ୍ସକ / ବୈଦ୍ୟ
6. Physician/ ଚିକିତ୍ସକ / ଡାକ୍ତର
7. Fatigue/କ୍ଳାନ୍ତି
8. Prenatal care/ ଗର୍ଭାବସ୍ଥା ସମୟରେ ଯତ୍ନ
9. Green vegetables/ସବୁଜ ପନିପରିବା
10. Meat/ମାଂସ
11. Pregnancy/ଗର୍ଭାବସ୍ଥା
12. Stomach ache/ପେଟ କାଟିବା

[For each pair, first ask: Is XXXX similar or different from YYYY/ପ୍ରତ୍ୟେକ ଯୋଡି ପାଇଁ ପ୍ରଥମେ ପଚାରନ୍ତୁ XXXX ସମାନ କିମ୍ବା YYYY ଠାରୁ ଅଲଗା ।

For peer review only - http://bmjopen.bmj.com/site/about/guidelines.xhtml

If Respondent says, “similar,” then ask: Would you say just a bit similar or very similar?

ଯଦି ଉତ୍ତରଦାତା ସମାନ କହନ୍ତି ତେବେ ପଚାରନ୍ତୁ ଆପଣ କିଛି ସମାନ କହୁଛନ୍ତି କିମ୍ବା ସମ୍ପୂର୍ଣ ସମାନ କହୁଛନ୍ତି?

If Respondent says, “different,” then ask: Would you say just a bit different or very different?

ଯଦି ଉତ୍ତରଦାତା ଅଲଗା କହନ୍ତି ତେବେ ପଚାରନ୍ତୁ ଆପଣ କିଛି ଅଲଗା କହୁଛନ୍ତି କିମ୍ବା ସମ୍ପୂର୍ଣ ଅଲଗା କହୁଛନ୍ତି?]

For peer review only

For peer review only - http://bmjopen.bmj.com/site/about/guidelines.xhtml

Card 1	Card 2	1 = very different/ସମ୍ପୂର୍ଣ ଅଲଗା<br>2 = somewhat differentକିଛି ଅଲଗା<br>3 = somewhat similarକିଛି ସମାନ<br>4 = very similarସମ୍ପୂର୍ଣ ସମାନ
1. IFA pill/ଆଇରନ ବଟିକା	2	
	3	
	4	
	5	
	6	
	7	
	8	
	9	
	10	
	11	
	12	
2. Clinic କ୍ଲିନିକ	3	
	4	
	5	
	6	
	7	
	8	
	9	
	10	
	11	
	12	

Card 1	Card 2	1 = very different/ ସମ୍ପୂର୍ଣ ଅଲଗା<br>2 = somewhat different କିଛି ଅଲଗା<br>3 = somewhat similar କିଛି ସମାନ<br>4 = very similar ସମ୍ପୂର୍ଣ ସମାନ
3. Kiosk	4	
	5	
	6	
	7	
	8	
	9	
	10	
	11	
	12	
4. SHG ସ୍ୱୟଂ ସହାୟକ ଦଳ	5	
	6	
	7	
	8	
	9	
	10	
	11	
	12	

Card 1	Card 2	1 = very different/ସମ୍ପୂର୍ଣ ଅଲଗା<br>2 = somewhat differentକିଛି ଅଲଗା<br>3 = somewhat similar କିଛି ସମାନ<br>4 = very similar ସମ୍ପୂର୍ଣ ସମାନ
5. Traditional healer ପାରମ୍ପାରିକ ଚିକିତ୍ସକ / ବୈଦ୍ୟ	6	
	7	
	8	
	9	
	10	
	11	
	12	
6. Physician ଡାକ୍ତର	7	
	8	
	9	
	10	
	11	
	12	
7. Fatigue କ୍ଲାନ୍ତି	8	
	9	
	10	
	11	
	12	

For peer review only - http://bmjopen.bmj.com/site/about/guidelines.xhtml

Card 1	Card 2	1 = very different/ ସମ୍ପୂର୍ଣ୍ଣ ଅଲଗା<br>2 = somewhat different କିଛି ଅଲଗା<br>3 = somewhat similar କିଛି ସମାନ<br>4 = very similar ସମ୍ପୂର୍ଣ୍ଣ ସମାନ
8. Prenatal care ଗର୍ଭାବସ୍ଥା ସମୟରେ ଯତ୍ନ	9	
	10	
	11	
	12	
9. Green vegetables ସବୁଜ ପନିପରିବା	10	
	11	
	12	
10. Meat ମାଂସ	11	
	12	
11. Pregnancy/ଗର୍ଭାବସ୍ଥା	12	

For peer review only

For peer review only - http://bmjopen.bmj.com/site/about/guidelines.xhtml

Section/ବିଭାଗ 3

Importance for Health and Well-Being/ସ୍ୱାସ୍ଥ୍ୟ ଏବଂ ଭଲ ରହିବା ପାଇଁ ମହତ୍ୱ

I will now show you the same 12 cards. Please put these cards in three different piles. In the first pile, put all the cards that you think are very important for your health and well-being. In the second pile, put all the cards that you think are just a little bit important for your health and well-being. In the third pile, put all the cards that you think are not important at all for your health and well-being. [Do the exercise and write response in the first table. Then shuffle the cards.]
ଏବେ ମୁଁ ଆପଣଙ୍କୁ ୧୨ଟି କାର୍ଡ ଦେଖାଇବି । ଏହି କାର୍ଡଗୁଡିକ ତିନୋଟି ଅଲଗା ଗଦାରେ ରଖନ୍ତୁ | ଆପଣ ଯେଉଁ ସବୁକାର୍ଡ ଗୁଡିକ ଭାବୁଛନ୍ତି ଆପଣଙ୍କର ସ୍ୱାସ୍ଥ୍ୟ ଏବଂ ଭଲରହିବା ପାଇଁ ଜରୁରୀ ସେଗୁଡିକ ପ୍ରଥମ ଗଦାରେ ରଖନ୍ତୁ | ଯେଉଁସବୁ କାର୍ଡଗୁଡିକ ଆପଣଙ୍କ ସ୍ୱାସ୍ଥ୍ୟ ଏବଂ ଭଲ ରହିବା ପାଇଁ କମ ଜରୁରୀ ସେଗୁଡିକ ଦ୍ୱିତୀୟ ଗଦାରେ ରଖନ୍ତୁ | ସେହିପରି, ଯେଉଁସବୁ କାର୍ଡଗୁଡିକ ଆପଣଙ୍କ ସ୍ୱାସ୍ଥ୍ୟ ଏବଂ ଭଲରହିବା ପାଇଁ ଜମା ଜରୁରୀ ନୁହେଁ, ସେଗୁଡିକ ତୃତୀୟ ଗଦାରେ ଅଛି ? (ଏହି ଖେଳଟିକୁ କରାନ୍ତୁ ଏବଂ ଉତ୍ତରକୁ ପ୍ରଥମ ଟେବୁଲରେ ଲେଖନ୍ତୁ | ତାହାପରେ କାର୍ଡଗୁଡିକ ମିଶାଇଦିଅନ୍ତୁ |)

Easy/ସହଜ

This time please think about how easy it is to get (or to use or to do) each item shown in the card. If you think the item is very easy to get (or to use or to do), put it in the first pile. If you think the item is sometimes easy and sometimes difficult to get (or to use or to do), put it in the second pile. If you think it is very hard to get (or to use or to do), put it in the third pile. [Write response in the second table. Then shuffle.]
ଦୟାକରି ଏଥର ଚିନ୍ତା କରନ୍ତୁ କାର୍ଡରେ ଦେଖା ଯାଇଥିବା ପ୍ରତ୍ୟେକ ଜିନିଷକୁ କେତେ ସହଜରେ ପାଇ ପାରିବେ । ଯଦି ଭାବୁଛନ୍ତି ଏହି ଜିନିଷଗୁଡ଼ିକ ବହୁତ ସହଜରେ ପାଇପାରିବେ (ବା ବ୍ୟବହାରରେ ଲଗାଇପାରିବେ ବା କାମରେ ଲଗାଇପାରିବେ) ସେଗୁଡିକ ପ୍ରଥମ ଗଦାରେ ରଖନ୍ତୁ | ଯଦି ଭାବୁଛନ୍ତି ଏହି ଜିନିଷଗୁଡ଼ିକ ପାଇବାକୁ ବେଳେବେଳେ ସହଜ ଏବଂ ବେଳେବେଳେ କଷ୍ଟ (ବା ବ୍ୟବହାରରେ ଲଗାଇପାରିବେ ବା କାମରେ ଲଗାଇପାରିବେ), ତାହାକୁ ତୃତୀୟ ଗଦାରେ ରଖନ୍ତୁ | ଯଦି ଭାବୁଛନ୍ତି, ଏହା ପାଇବାକୁ (ବା ବ୍ୟବହାରରେ ଲଗାଇପାରିବେ ବା କାମରେ ଲଗାଇପାରିବେ) ବହୁତ କଷ୍ଟସାଧ ତାହାକୁ ତୃତୀୟ ଗଦାରେ ରଖନ୍ତୁ | (ଉତ୍ତରଗୁଡିକ ଦ୍ୱିତୀୟ ଟେବୁଲ ରେ ଲେଖନ୍ତୁ ଏବଂ ସେଗୁଡିକ ପରେ ମିଶାଇଦିଅନ୍ତୁ |)

Like/ପସନ୍ଦ

Finally, we will now sort these into three piles according to how much you like each card. If you like it a lot, put it in the first pile. If you like it just a bit or dislike it just a bit, put it in the second pile. And, if you dislike it a lot, put it in the third pile. [Record in the third table] ଶେଷରେ, ଆପଣ ପ୍ରତ୍ୟକ କାର୍ଡକୁ କେତେ ପସନ୍ଦ କଲେ, ସେହି ଅନୁସାରେ ଆଗେ ଏହାକୁ ତିନୋଟି ଗଦାରେ ସଜାଇ ରଖିବା | ଯଦି ଆପଣ ଏହାକୁ ବହୁତ ପସନ୍ଦ କରନ୍ତି, ତାହେଲେ

For peer review only - http://bmjopen.bmj.com/site/about/guidelines.xhtml

For peer review only

ତାକୁ ପ୍ରଥମ ଗଦାରେ ରଖନ୍ତୁ | ଯଦି ଆପଣ ଏହାକୁ କମ ପସନ୍ଦ କରନ୍ତି ବା କମ ପସନ୍ଦ କରନ୍ତି ନାହିଁ, ତାହାକୁ ଦ୍ଵିତୀୟ ଗଦାରେ ରଖନ୍ତୁ | ଯଦି ଆପଣ ସମ୍ପୂର୍ଣ୍ଣ ପସନ୍ଦ କରନ୍ତି ନାହିଁ, ତାହାକୁ ତୃତୀୟ ଗଦାରେ ରଖନ୍ତୁ) (ତୃତୀୟ ଟେବୁଲରେ ଲେଖନ୍ତୁ)

Write the card number in each column.

PILE 1: Very important ବହୁତ ଜରୁରୀ	PILE2: Somewhat important କିଛିମାତ୍ରାରେ ଜରୁରୀ	PILE 3: Unimportant ଜରୁରୀ ନୁହେଁ

PILE 1: Very easy to get ବହୁତ ସହଜରେ ପାଇ ହେବ	PILE 2: Somewhat easy କମ୍ ସହଜରେ ପାଇ ହେବ	PILE 3: Very hard to get ପାଇବାକୁ ବହୁତ କଷ୍ଟସାଧ

PILE 1: Like a lot ବହୁତ ପସନ୍ଦ କରନ୍ତି	PILE 2: Like somewhat କିଛି ପସନ୍ଦ କରନ୍ତି	PILE 3: Dislike ପସନ୍ଦ କରନ୍ତି ନାହିଁ

For peer review only

For peer review only - http://bmjopen.bmj.com/site/about/guidelines.xhtml

Supplement 2 – Focus Group Guide

Before Beginning the Interview:

- Welcome participant and introduce yourself

Hello, and thank you for speaking with me today. My name is _______ and I am a field researcher at DCOR. I am conducting a research study to understand the attitudes and beliefs of people in this community. Thank you so much for agreeing to participate and taking time out of your day.

Do you have any questions about the research and your participation before we start?

Warm-up Questions

I'm new to this community, [name District], can you tell me a little bit about it? For example, what kind of food do people eat here? And what do people do to earn money?

Women's Role in the Community

What are some of the typical things that women in this community do throughout the day? (probe: things like cooking, cleaning, taking care of kids, working outside the home, spending time with her husband).

Do women in this community have enough energy or time to these things each day?

- Look after her kids?
- Work outside the home?
- Cook?
- Spend time with her husband?
- Have more intimate time with her husband?

How are men and women treated differently in this community?
For women's life in general, are things getting better or worse?
- in her role as a mother?
- in her role as a wife?
- in her role as a daughter in law?

What are some of the concerns that women in this community have? (probe: concerns about health, money, their family, themselves, their future)

- Some people think that (woman's name) should first take care of her husband and kids before she worries about her own health. Other people think that she should first take care of her own health.

  How do you think most women in this community feel about that? What about most men?

  In some families in Odisha, women eat after their husband, children and mother in law eat. In others, women eat at the same time. What do you think about that? Is this changing?

For peer review only - http://bmjopen.bmj.com/site/about/guidelines.xhtml

<u>Anemia and Iron Folic Acid (IFA) Knowledge</u>

(Show an IFA tablet and liquid IFA)

Can someone tell me what this is? (probe: What does it do?)

Have you ever heard of anemia?

Many people in this community may not use the word "anemia" but they may have other words or phrases to talk about anemia. Can you tell me what some of those phrases are?

Tell me a little bit about what happens when someone has <u>anemia</u> (use the word or phrase identified above instead of "anemia"). Tell me how this person feels or how this person acts when they have <u>anemia</u>.

What do you think causes <u>anemia</u>? (probe: Not eating enough iron rich foods?)

What do you think makes anemia go away? (Probe: Can IFA tablets help? How about changing your diet? What kind of foods might help it go away?)

<u>Anemia Related Behavior</u>

Ok, now we're going to make up a character. She is a female aged 23 years old. What should we call her? (Ask for suggestions and decide on a name together). She just got married and is 3 months pregnant with her first child. Her doctor just told her she has anemia.

The doctor told her to take iron tablets.

- If (same woman's name) wanted to get IFA tablets, where could she get them? How difficult is it to *get* IFA tablets?
  - Can you tell me what she likes about taking the tablets and what she doesn't like? (PROBE by asking more likes and dislikes)
  - Apparently, some women stop taking those tablets. Can you tell me why they stop?
  - Can you tell me what good things may happen if someone takes the tablets?
  - What do you think would help her take the tablets? Do you think (woman's name)'s husband will support her to take the tablets? Not support her to take them? Or not say anything? (Ask for examples)
  - What about (woman's name)'s mother in law? Will she support her in taking the tablets? Why or why not?

<u>IFA Norms</u>

- Is Anemia is a problem in the community? If yes How much of a problem?
- In general, who is typically expected to take IFA tablets? (Probe: pregnant women, adolescent girls, non-pregnant women?)
- Please think about most women like (name) who live in this community. How many of them take IFA tablets? Some? Few? Most?
- Is there anyone in (woman's name) family (or her husband's family) that she can talk to about taking IFA tablets? How much do you think she'll listen to what they have to say about IFA tablets?

<u>Information Sources</u>

For peer review only - http://bmjopen.bmj.com/site/about/guidelines.xhtml

Imagine another woman who is not pregnant but interested in learning more about her health. What should we call her? (Ask for suggestions and decide on a name together).

Where does she go for information about health related matters?

Where can she get information about pregnancy, anemia, and IFA tablets from?

- How easy is it for her to get information about pregnancy, anemia, and/or IFA tablets? What difficulties would come up when trying to get this information?
- When a woman gets IFA tablet, what kind of information does she get about them?
- Probe: Dose, when to take or why to take it?
- For those of you that participate in self-help groups, can you tell me what kind of things you discuss?

Closing

That is the end of the questions I have for you, but do you have anything else you'd like to add to the discussion? Any little stories about anemia or IFA use in this community?

As a reminder, please do not share anything we spoke about today with anyone outside of this group.

Any questions?

Thank you for your time.

For peer review only

Is fatigue a cue to take iron supplements in Odisha India? A mixed methods investigation

**SRQR Topic number**	**SRQR Topic**	**Page numbers (and line numbers if relevant)**
	Title and Abstract	
S1.	Title	p. 1; line 1
S2.	Abstract	p. 2
	Introduction	
S3.	Problem formulation	p. 5 - 8
S4.	Purpose or research question	p. 9; lines 1-2 & 5-6
	Methods	
S5.	Qualitative approach and research paradigm	p. 9 - 14
S6.	Research characteristics and reflexivity	p. 9 - 10
S7.	Context	p. 9 - 10
S8.	Sampling strategy	p. 10 – 11
S9.	Ethical issues pertaining to human subjects	p. 11; lines 13 - 23
S10.	Data collection methods	p. 12 - 13
S11.	Data collection instruments and technologies	bottom of page 12 to top of page 13
S12.	Units of study	p.14
S13.	Data processing	p.14
S14.	Data analysis	p.14
S15.	Techniques to enhance trustworthiness	p.9-10
	Results/Findings	
S16.	Synthesis and interpretation	p. 15 - 18
S17.	Links to empirical data	p. 15 - 18
	Discussion	
S18.	Integration with prior work, implications, transferability, and contributions to the field	p. 21 - 22
S19.	Limitations	p. 22
	Other	
S20.	Conflicts of interest	p. 24
S21.	funding	p. 24

For peer review only

BMJ Open

Is fatigue a cue to obtain iron supplements in Odisha India? A mixed methods investigation

Journal:	*BMJ Open*
Manuscript ID	bmjopen-2020-037471.R2
Article Type:	Original research
Date Submitted by the Author:	10-Aug-2020
Complete List of Authors:	Yilma, Hagere; Prevention and Community Health<br>Sedlander, Erica; George Washington University Milken Institute of Public Health, Prevention and Community Health<br>Rimal, Rajiv; Johns Hopkins Bloomberg School of Public Health Center for Teaching and Learning, Health, Behavior and Society<br>Pattnaik, Soumik; D-COR Consulting
**Primary Subject Heading**:	Global health
Secondary Subject Heading:	Public health, Global health
Keywords:	PUBLIC HEALTH, QUALITATIVE RESEARCH, Nutrition < TROPICAL MEDICINE, Anaemia < HAEMATOLOGY

SCHOLARONE™
Manuscripts

I, the Submitting Author has the right to grant and does grant on behalf of all authors of the Work (as defined in the below author licence), an exclusive licence and/or a non-exclusive licence for contributions from authors who are: i) UK Crown employees; ii) where BMJ has agreed a CC-BY licence shall apply, and/or iii) in accordance with the terms applicable for US Federal Government officers or employees acting as part of their official duties; on a worldwide, perpetual, irrevocable, royalty-free basis to BMJ Publishing Group Ltd ("BMJ") its licensees and where the relevant Journal is co-owned by BMJ to the co-owners of the Journal, to publish the Work in this journal and any other BMJ products and to exploit all rights, as set out in our licence.

The Submitting Author accepts and understands that any supply made under these terms is made by BMJ to the Submitting Author unless you are acting as an employee on behalf of your employer or a postgraduate student of an affiliated institution which is paying any applicable article publishing charge ("APC") for Open Access articles. Where the Submitting Author wishes to make the Work available on an Open Access basis (and intends to pay the relevant APC), the terms of reuse of such Open Access shall be governed by a Creative Commons licence – details of these licences and which Creative Commons licence will apply to this Work are set out in our licence referred to above.

Other than as permitted in any relevant BMJ Author's Self Archiving Policies, I confirm this Work has not been accepted for publication elsewhere, is not being considered for publication elsewhere and does not duplicate material already published. I confirm all authors consent to publication of this Work and authorise the granting of this licence.

For peer review only

Title: Is fatigue a cue to obtain iron supplements in Odisha India? A mixed methods investigation

Authors: Hagere Yilma, MPH[1], Erica Sedlander, DrPH, MPH[1], Rajiv N. Rimal, PhD[2], Soumik Pattnaik, MS[3]

[1] George Washington University, Milken Institute School of Public Health, Department of Prevention and Community Health
[2] Johns Hopkins University Bloomberg School of Public Health, Health, Behavior, and Society
[3] DCOR Consulting

Hagere Yilma, MPH (corresponding author)
950 New Hampshire, Department of Prevention and Community Health
202.994.1486
https://orcid.org/0000-0002-4042-3484
hyilma@gwu.edu

Erica Sedlander, DrPH, MPH
950 New Hampshire, Department of Prevention and Community Health
202.994.0849
esedlander@gwu.edu

Rajiv N. Rimal, PhD
624 N. Broadway, Department of Health, Behavior, and Society
rimal@jhu.edu

Soumik Pattnaik, MS
H.B Colony, Punjabi Colony, Satya Nagar, Bhubaneswar, Odisha, India
+91 94384 63984
soumik.dcor@gmail.com

For peer review only

Abstract

Objectives: This study aims to understand if fatigue, the main symptom of anemia, is a health concern that cues women and their referent groups to obtain iron folic acid supplements.

Design: This is a mixed methods study that consisted of a cross-sectional perceptual mapping and card sorting activity along with 16 focus group discussions with women of reproductive age, mothers-in-law, and men. Participants of the perceptual mapping and card sorting activity were asked to compare images of anemia related items and concepts. Participants in the focus group discussions were asked about their daily life, aspirations and concerns among women, and perceptions of anemia and iron supplementation in the community. The quantitative data were analyzed through multidimensional scaling and ANOVAs in SPSS. The qualitative data were analyzed through applied thematic analysis using NVIVO.

Setting: Bhubaneswar, Odisha, India

Participants: Women of reproductive age (*n* =30), mothers-in-law (*n* = 30), and married men (*n* = 30) were randomly selected to participate in the perceptual mapping and card sorting exercise. A separate sample of each group was randomly selected for the focus group discussions (*n* = 148).

Primary and secondary outcome measures: We collected perceptions of dissimilarity between anemia-related items, including fatigue and medical items; the extent to which these items were perceived as important to health or likable; and qualitative information about gender norms.

Results: Cognitive maps and card sorting revealed that fatigue was conceptualized distinctly from items related to medical treatment and that perceptions around fatigue's

importance to health were low. Women from the focus groups reported that fatigue is a regular part of their daily life.

Conclusion: Our results indicate that fatigue is currently not an adequate cue to seek treatment, perhaps due to the normalization of fatigue as a part of women's daily life.

Keywords: anemia; gender norms; iron folic acid; perceptual mapping; women's health

For peer review only

For peer review only - http://bmjopen.bmj.com/site/about/guidelines.xhtml

Strengths and Limitations of the study

- We adapted a marketing methodology known as perceptual mapping, coupled with a card sorting exercise and focus group discussions to understand how women of reproductive age, and their relevant referent groups (i.e., mothers-in-law and men) perceive fatigue.
- Qualitative focus groups were used to minimize the ambiguity of the perceptual mapping plots.
- The size of each subsample was small (n=30 each), reducing our ability to find significant differences from the card sorting activity.

For peer review only

Background

Anemia is a condition characterized by a low red blood cell count that affects a quarter of all people worldwide.[1] If left untreated, anemia can lead to several negative health outcomes, including poor mental health,[2-3] reduced work capacity,[4] pregnancy complications,[5-6] and sometimes death.[3] Women of reproductive age are the most vulnerable to anemia,[1] as blood loss during menstruation and childbirth raises their risk.[7-10] Recent research suggests that inequitable social structures may exacerbate anemia prevalence in women, particularly as it relates to gender norms.[11-14, 1] Gender norms are a subset of social norms, which refer to expectations around how people of a specific gender are expected to behave.[15]

The World Health Organization (WHO) has made anemia one of six global nutritional targets.[16] Iron and folic acid supplementation is an effective strategy for the prevention and treatment of anemia in low- and middle- income countries.[17-19] Dosage recommendations vary by age, gender, and pregnancy status, but WHO recommends regular iron supplementation for non-pregnant women of reproductive age in countries where prevalence exceeds 40%.[17]

In India, where over half of women of reproductive age are anemic, [20] the promotion of iron supplements to children and women of reproductive age has been a part of the nutritional agenda since 1970.[21-22] However the expansion of promotion to non-pregnant, out-of-school women only began in 2013 through the National Iron Plus Initiative.[23] Despite India's recent life-course approach to promote iron supplements to all women, consumption remains low and anemia prevalence is still high.[20, 24] This may be due to gaps in current iron supplement distribution practices in the Indian health

For peer review only - http://bmjopen.bmj.com/site/about/guidelines.xhtml

system. Distribution outside of clinical settings target pregnant women or in-school adolescents; frontline workers provide tablets to pregnant women, and adolescent girls can get iron supplements weekly in school. Out-of-school, non-pregnant women do not have this access and must first go to a health facility to get tested before receiving treatment, which is typically iron supplementation for mild and moderate forms of anemia.[25, 26] This process requires an ability to detect anemia symptoms within oneself and seek medical care.

However, the primary manifestation of anemia, fatigue, may be ignored as a sign of illness.[27-28] In a review of the evolving conceptualizations of fatigue across time, Loriol (2017) notes that, historically, fatigue is considered a necessary outcome for physical energy expenditure and, consequently, commonly attributed to a hard day's work. The result is a prevailing social expectation that the more the one expends physical energy, the more fatigued one is. This is seen in the inverse association between fatigue and socioeconomic status. [29] Therefore, women who exert a lot of physical energy in a day may not feel their fatigue is cause for alarm. Rather, they may feel that the fatigue they experience is a normal outcome of their hard work.

It is no surprise then that more than 80% of women in Odisha, India do not recognize tiredness or weakness as symptom of anemia. [26] Studies in similar cultures have also shown that women may not readily associate fatigue with illness.[30] In fact, anemic women who reported fatigue during pregnancy did not seek medical attention because they believed their anemia-related fatigue was just a normal part of pregnancy.[31-32]

For peer review only - http://bmjopen.bmj.com/site/about/guidelines.xhtml

FATIGUE AS A CUE TO SEEK TREATMENT

While the anemia literature among non-pregnant women in India is sparse, research in other parts of the world has shown that fatigue represents a conceptually different dimension of symptoms than somatic symptoms, even when both forms share an underlying cause.[33-34] Further, Ball et al. (2017) demonstrate that women's perceptions of fatigue symptoms are influenced by familial factors, whereas men hold perceptions of fatigue that are influenced by genetic factors.[33] A study in an area with high anemia prevalence showed that culture and gender norms can shape perceptions of anemia, which can in turn influence treatment-seeking behaviors among non-pregnant women.[13] Therefore, a similar normalization of fatigue may exist among non-pregnant women in rural India, as expectations for labor may lead to exhaustion that is difficult to disentangle from anemia-related fatigue. In fact, fatigue is commonly associated with activities like caretaking and employment in women but not men. [35]

In this paper, we explore the extent to which women perceive fatigue as a cue to seek medical care. While the literature around the conceptualization of fatigue as a symptom of illness is limited, recent research in India has shown that inequitable gender norms may indirectly affect a woman's ability or desire to access iron supplements.[11-14, 36] Other studies also show that women in Odisha tend to have less power within the household than their husbands, and carry the majority of the domestic workload burden.[37] As daily life is characterized by large amounts of physical labor (for both men and women), fatigue may be considered a normal part of everyday life, and thus, not a cue to obtain iron supplements. Clearly, knowledge about the prevalence and risks of anemia is critical as risk perception may be connected to gender norms. If women believe

For peer review only - http://bmjopen.bmj.com/site/about/guidelines.xhtml

that fatigue is just a necessary outcome of their role as women, rather than a symptom of illness, increasing risk perception may be an important first step.

Additionally, studies in India have highlighted the influence of shared-decision making within households as a key factor in anemia prevalence among women.[38-39] To our knowledge, how this relates to different conceptualizations of fatigue by various household members – husbands, mothers-in-law, and others – has not yet been explored. Yet, this is an important consideration: If women themselves do not equate fatigue with a need to seek medical care, do influential others in her home view fatigue in the same manner? Hence, our research questions are:

RQ1. Are perceptions of fatigue a cue to obtain iron supplements among women of reproductive age in Odisha, India?

RQ2. What are the differences in perceptions of fatigue across women's referent groups (their mothers-in-law and husbands)?

Methods

We used multiple methods to understand how conceptualizations of fatigue may relate to treatment-seeking behaviors among women of reproductive age in Odisha, India. We report findings from two methods, one quantitative and the other qualitative, used in this paper. First, we used perceptual mapping techniques and a card sorting activity to understand how our participants conceptualized fatigue.

Perceptual mapping is often used in commercial marketing [40-42] but it has recently gained momentum in health behavior communication. [43-47] In the current

For peer review only - http://bmjopen.bmj.com/site/about/guidelines.xhtml

study, we expand on the public health application of perceptual mapping by using this technique to understand how women understand fatigue in relation to items associated with the acquisition of iron tablets. We follow the perceptual mapping activity with a card sorting exercise to understand if cognitive (i.e. perceived importance to health) and affective (i.e. dislike) perceptions could inform the results of the perceptual maps.

While perceptual maps can highlight conceptual associations (or disassociations) between concepts, it is difficult to pinpoint the dimensions of perception around which these similarities or differences exist. We included a card sorting activity to probe the cognitive and affective dimensions of perception. However, a qualitative component is also needed to delineate why these associations or disassociations may exist. Therefore, we followed the quantitative methods with qualitative focus group discussions to triangulate our findings and identify the social and cultural underpinnings of how fatigue is conceptualized.

All components of this study come from formative research conducted for the Reduction in Anemia through Normative Innovations (RANI) Project. The RANI Project is implementing and evaluating a social norms-based anemia reduction intervention. The overall goals of the formative research components were larger than the research questions presented here; all of the goals of the formative research beyond this study are outlined the RANI project formative research protocol. [48]

Sampling

The perceptual mapping and card sorting activity collected data from a different sample than the focus group discussion to accommodate the larger formative research goals of the methods and limit participant burden. However, all samples came from the

For peer review only - http://bmjopen.bmj.com/site/about/guidelines.xhtml

Angul District of Odisha, India and shared the same random sampling procedure. To do so, researchers first tabulated all the homes in each of the four villages where we worked and obtained the total number of eligible participants. Based on the number of participants needed for the focus groups, against a sampling frame that consisted of the entire village, we used a proportional skip pattern that began with a randomly selected initial participant to identify households from which to select every succeeding participant for each sub-group. From the remaining households, a similar selection process was applied to select the participants for the perceptual mapping and card sorting activity.

We conducted 16 focus groups with women of reproductive age, mothers-in-law, adolescent girls, and husbands (n = 124). The sample description is published elsewhere.[11] The final sample for the perceptual mapping and card sorting exercise comprised women of reproductive age (n=30), mothers-in-law (n=30), and men (n=30). All participants were required to live in the Angul District, speak Odiya, and give written consent before participating. Parental permission was obtained for all participants younger than 18 years old. A small gift of a bowl and spoon was given as incentive for participating.

Patient and Public Involvement

This study did not involve patients or the public in its development or implementation. Upon completion of the study, results were shared with various stakeholders at a two-day convening in Bhubaneswar, Odisha, India.

Procedure

For peer review only - http://bmjopen.bmj.com/site/about/guidelines.xhtml

The XXX University Institutional Review Board (IRB) and a local ethics board, D-COR Consulting Institutional Ethics Committee (IEC) reviewed and approved this study before recruitment began.

Perceptual Mapping. During the perceptual mapping exercise, we showed participants two cards at a time, each with a picture of a different anemia-related item, and we asked them to compare how similar the two were to each other. We recorded their rating on a scale from 1 to 4, where 4 indicated the most similarity. We had 12 cards in all, and all 66 permutations (1 versus 2, 1 versus 3, … 11 versus 12) were shown to each participant. A more detailed explanation of the perceptual mapping procedure can be found in the Formative Research Protocol. [48]

Card Sorting. We also asked participants to do a card sorting exercise upon completing the perceptual mapping exercise. In this portion of the study, we asked participants to place the same cards into three piles based on the items level of importance for their health and well-being. Upon completing this task, participants were then asked to sort the cards into another set of three piles based on the likability of the item. Data collectors recorded the compositions of each pile.

Focus Group Discussions. Trained researchers (native speakers from Odisha India) conducted all focus groups, and we matched interviewer and interviewee on gender. For each focus group, one member of the research staff observed the group while taking notes and researchers wrote field notes after each interview. Audio recordings were transcribed in Odiya and subsequently translated into English for data analysis. A more detailed description of the focus group discussion procedures can be found in a separate study. [11]

For peer review only - http://bmjopen.bmj.com/site/about/guidelines.xhtml

Instruments

All instruments were developed based on discussions with local researchers and a review of the literature to ensure cultural and theoretical relevance.[25, 49-50] For the perceptual mapping and card sorting activity, we used large cards with the images of interest printed on them. The 12 cards included an image of one of the following: iron tablets, a clinic, pharmaceutical kiosks, women empowerment groups known as self-help groups, traditional healer, physician, fatigue, prenatal care, green vegetable, meat/fish, pregnancy, or stomach ache. Labels of each item were also included on each card. Our colleagues in Odisha, India assessed the cards for face validity. Interview guides for the focus group discussions covered general questions about what women do on a typical day, their concerns and aspirations, and roles in the family and community. The questionnaire for the perceptual mapping and card sorting activity (Supplement 1), along with the focus group discussion guides (Supplement 2) are included as supplementary material.

Analysis

Perceptual Maps. Participant ratings were recoded to indicate dissimilarity and then organized into matrixes. We used multidimensional scaling (MDS) to graphically plot the averaged dissimilarities of the overall sample as well as each subgroup recorded (i.e., women of reproductive age, mothers-in-law, and men; Figures 1-3). MDS is a common method to analyze perceptual mapping data. [51] Theoretically, the resulting maps can display items on multiple axes to represent underlying dimensions of perception. For simplicity and ease of interpretation, they are often distilled down to two (x and y) dimensions. The maps display dissimilarity so that the further two points are

For peer review only - http://bmjopen.bmj.com/site/about/guidelines.xhtml

from each other, the more the participants perceived dissimilarity between the two items. Conversely, the closer the two points are on the map, the more the participants perceived similarity.

Card Sorting. We converted the responses from the card sorting exercise into ranks from 0-2, where a score of 0 was given to cards that were placed in the "not very important to health" pile or the "dislike" pile, and a score of 2 was given to the cards that were in the "very important to health" pile or "like a lot" pile, for their respective parameters. Scores for liking were reverse coded so that higher values indicated a dislike. We analyzed the card sorting data using an ANOVA across the three participant groups. We conducted post-hoc analyses to determine homogenous subsets when omnibus differences were detected. There we no missing responses. All analysis were done in SPSS v.25.

Focus Group Discussions. We uploaded transcripts and the codebook into NVivo v.12 for analysis (Nvivo, 2015). We developed a draft codebook then applied thematic analysis to characterize and subsequently modify emergent themes through an iterative approach that combined data collection and analysis concurrently. [52] This allowed us to determine when saturation occurred, when no new themes emerged from the data.

Research Team and Reflexivity

Of the four authors, one is from Angul, India, one is from Nepal, and two are from the United States. Their backgrounds vary from overseeing data collection efforts to social norms/gender norms, and global behavioral health. Each researcher holds a master's and/or doctoral degree and their familiarity with anemia in India range from

For peer review only - http://bmjopen.bmj.com/site/about/guidelines.xhtml

academic research to personal experience. Three of the authors, SP, ES, and RR, were present for the data collection training and piloting. One of the authors, SP, was present for all data collection. None of the authors have any personal relationship to the participants or any direct benefit from examining the relationship between anemia and fatigue.

For peer review only

Results

Sample

The demographic distribution of each subgroup can be found in Table 1. The average age of men in the sample was 35 years (*SD* = 6.00), and that of women was 28 years (*SD* = 4.76). The average age of mothers-in-law was 56 years (*SD* = 7.58). The majority of women of reproductive age had completed at least secondary school, while the majority of men had not completed secondary school. The majority of mothers-in-law had not received any education. Additionally, the majority of participants had at least two children. All but one participant was also married; the singular participant who was unmarried was widowed. All participants were Hindu.

While the majority of women of reproductive age had taken iron folic acid in the past (86.7%), very few were taking it at the time of the survey (10%). Only about a quarter of mothers-in-law had ever taken iron folic acid, but 9.1% were taking it at the time of the survey. None of the men surveyed reported currently taking iron folic acid, and only 13.3% had ever taken it. About half of women of reproductive age had ever received an anemia diagnosis, and 14.3% reported currently being anemic at the time of

For peer review only - http://bmjopen.bmj.com/site/about/guidelines.xhtml

the survey. The demographic distribution of participants who took part in the focus group discussions was similar to the participants who took part in the perceptual mapping activity. [11]

Perceptions of Fatigue

Perceptual maps. The perceptual maps for women of reproductive age show a conceptual separation, as evidenced by their spatial distance, between fatigue and the practical iron folic acid distribution channels for non-pregnant women; one quadrant of the map contains fatigue, while a separate one contains the items related to the iron supplement distribution channels for non-pregnant women, including physicians, clinics, and pharmaceutical kiosks (Figure 1). These three items are clustered together in one quadrant, indicating that they are perceived as similar to each other. Iron folic acid is also plotted in the same quadrant as this cluster, indicating that women of reproductive age may conceptualize physicians, clinics, and pharmaceutical kiosks as links to iron supplements. The separation of fatigue from these iron supplement distribution channels indicates that fatigue may not act as a cue to obtain iron supplements.

Card Sorting Exercise. Confirming these findings, the card sorting exercise showed that women of reproductive age were less likely to report fatigue as important to health compared to items related to the iron supplement distribution channels for non-pregnant women (Table 2). Fatigue was considered to be only somewhat important to health by women (M = 0.77, SD = 0.73), while the iron supplement channels, including physicians (M = 1.80, SD = 0.55), clinics (M = 1.80, SD = 0.48), and pharmaceutical kiosks (M = 1.77, SD = 0.50), were considered very important to health.

For peer review only - http://bmjopen.bmj.com/site/about/guidelines.xhtml

Differences in the conceptualization of fatigue and the items related to iron supplement distribution channels for non-pregnant women may also be explained by differences in affective ratings (Table 2). Fatigue was reported to be disliked by women (*M* = 1.47, *SD* = 0.68), while the other iron folic acid channel-related items were hardly disliked at all (physician: *M* = 0.20, *SD* = 0.41; clinics: *M* = 0.23, *SD* = 0.50; pharmaceutical kiosks: *M* = 0.27, *SD* = 0.58).

Focus Group Discussions. Women reported that fatigue, and oftentimes, extreme fatigue, was a normal part of daily life. They accepted fatigue as part of their role as wives and mothers; they take care of all of the household responsibilities including all childcare responsibilities, serve their husband and in-laws, and often work outside of the home to earn extra money for the family. One woman said, *"We have to work all the time, go to the mountains and fields, have children to take care of. We get no time to rest. We work in unhappiness and eat in pain"* (woman of reproductive age). Women did not think of fatigue as something that they could change and they did not associate their own fatigue with the possibility of having anemia. Although most women knew that fatigue is a symptom of anemia, they underestimated the prevalence of anemia in their village despite endemic rates. A frontline health worker said, *"In our village, in a year, 2-3-4 women have it [anemia]...mainly pregnant women"* (frontline health worker). This is, of course, a far cry from what the actual numbers would be, based on data from the National Family Health Survey, [20] which shows that over half of women of reproductive age are anemic.

Perceptions of Fatigue across Referent Groups

For peer review only - http://bmjopen.bmj.com/site/about/guidelines.xhtml

Perceptual maps. Men (Figure 2) and mothers-in-law (Figure 3) also perceived fatigue as different from the other items related to the iron supplement distribution channels. The perceptual map generated according to men's responses displays physicians, pregnancy, and prenatal care as plotted closely together in the same quadrant. This cluster also contains iron supplements, indicating that men understand these items as existing in the same cognitive space. Similarly, the perceptual map generated for mothers-in-law displays physicians, clinics, and pharmaceutical kiosks as a cluster plotted near the plot-point for iron supplements. Fatigue, however, is plotted in a separate quadrant from these items related to the iron supplement distribution channels for non-pregnant women. The separation of fatigue from these channels indicates that fatigue may not act as a cue to obtain iron supplements among husbands and mothers-in-law.

Card Sorting Exercise. We also explored differences in perception across participant groups through the card sorting analysis. Perceptions of importance to health didn't differ across participant groups for any of the anemia-related items shown, with the exception of fatigue ($F = 9.66$, $p < 0.001$) and stomachache ($F = 5.76$, $p < 0.01$), which were significantly different between women and their referent groups. Affective perceptions (i.e. dislike) related to fatigue were also statistically different across the three groups as well ($F = 8.29$, $p < 0.001$).

Post-hoc Least Significant Differences (LSD) tests revealed that women and men had similar perceptions of fatigue across these cognitive and affective dimensions (i.e., importance to health and dislike), but mothers-in-law had significantly different perceptions. Fatigue was perceived with the same level of importance for health by both men ($M = 0.80$, $SD = 0.61$) and women ($M = 0.80$, $SD = 0.61$). However, mothers-in-law

For peer review only - http://bmjopen.bmj.com/site/about/guidelines.xhtml

had significantly lower perceptions about the importance of fatigue ($M = 0.17$, $SD = 0.53$) than men ($p < 0.001$) and women ($p < 0.001$). Additionally, men reported a similar dislike of fatigue as women ($M = 1.23$, $SD = 0.73$, n.s.) while mothers-in-law disliked fatigue to a much greater extent ($M = 1.87$, $SD = 0.35$, $p < 0.05$).

Focus Group Discussions. Men acknowledged that women bear the brunt of the work at home and often face extreme fatigue but did not make the connection that anemia could be at play. A husband from a focus group said, *"They [women] don't have time to do all these works. To do the outside works after completing the household works, they don't get enough time"* (husband). Mothers-in-law felt that their daughters-in-law had it "easier" today than when they were young and showed less empathy than men for their daughters-in-law heavy workload. They also mentioned that younger women today seek medical care more often than their generation. A mother-in-law said, "*We never had those tablets [iron supplements]. We had no idea that we are pregnant until the 5th month but women of today know everything from the 1st month and run to the hospital*" (mother-in-law).

Discussion

This study aimed to understand if the conceptualization of fatigue allowed it to act as a cue for illness in women as well as referent others in their network (i.e., mothers-in-law and husbands). We found that fatigue may not act as a sufficient cue for women to seek treatment for anemia. Nor is fatigue conceptualized in a similar manner across the referent groups. These findings were notable in the perceptual maps and card sorting activity, and further manifest through the focus group discussions.

For peer review only - http://bmjopen.bmj.com/site/about/guidelines.xhtml

FATIGUE AS A CUE TO SEEK TREATMENT

The perceptual mapping activity showed a distinction between fatigue and the cluster of items related to the iron supplement distribution channels, which included physicians, clinics, and pharmaceutical kiosks. These clinical items represent necessary steps in the iron folic acid acquisition process for non-pregnant women; the disassociation between fatigue and these clinical items indicates the conceptual distinction between fatigue and a need for medical care. Additionally, the card sorting exercise showed cognitive and affective gaps in perceptions between fatigue, on the one hand, and the aforementioned clinical items, on the other. In particular, women did not perceive fatigue as an important consideration for their health, although they reported dislike for it. These findings are in line with previous literature that discusses the normalization of fatigue as an outcome of hard work rather than an illness. [29]

The qualitative results support the lack of association between fatigue and medical care in the perceptual mapping and card sorting activity. The focus group discussions clarify these findings through the identification of gender norms that may set the conditions for what mental and physical phenomenon are perceived as illness.[11-14, 36] Women in the focus group discussions highlight a prevailing gender norm that women are expected to maintain a large amount of the household duties through the exertion of physical labor, which in turn, can lead to the expectation of fatigue.[29] Women in the focus groups also indicated that they prioritize the wellbeing of their family over their own. Thus, they accept the fatigue that they feel as a normal outcome of their household role. These unequal gender norms that place the majority of household duties on the woman may normalize fatigue in women and negatively affect their risk

For peer review only - http://bmjopen.bmj.com/site/about/guidelines.xhtml

perception. Thus, fatigue is not an adequate cue that illness is present, and consequently, does not act as a cue to seek treatment.

This study also aimed to investigate conceptualizations of fatigue in mothers-in-law and men. The perceptual maps created for mothers-in-law showed a slightly larger dissociation between fatigue and the clinical items related to ion supplementation than was observed in women of reproductive age. While perceptual mapping methods do not allow for the determination of statistically significant differences, the results of the focus group discussion show that the older generation of women believe that younger women have more sensitive perceptions of health. The mothers-in-law in this study harbored attitudes that were even more dismissive of fatigue than men or younger women; they did not consider fatigue important to health. These results indicate that the normalization of symptoms, like fatigue, may be stronger among older generations of women.

We found a different conceptualization of fatigue in men. The card sorting activity revealed a smaller gap in cognitive and affective perceptions of fatigue; while men may consider fatigue a low health concern, they also do not mind it very much. The results of the focus group discussions offer the explanation that men may not mind fatigue as much as the older generations of women because they are subject to different expectations of labor. The men in the focus group discussions show empathy for women who complete the lion’s share of household work and often work outside the home. The results of the qualitative investigations coupled with the quantitative findings indicates that fatigue is normalized to a lesser extent in men than it is in women.

The conceptualization of fatigue as illness is critical for non-pregnant, out-of-school women who are currently not given iron supplements as part of regular care by

For peer review only - http://bmjopen.bmj.com/site/about/guidelines.xhtml

front line health workers. If women do not perceive illness when fatigue is present, they may not seek treatment for it (including asking for iron supplements) unless they are pregnant. This may exacerbate an existing gap in iron supplement distribution to non-pregnant, out-of-school women, who make up the largest proportion of women with anemia. [20] Future anemia reduction interventions should take into account the social and cultural underpinnings related to perceptions of illness among women.

Studies show that mothers-in-law are intimately involved in the decision-making processes among anemic women in India. [39, 53-54] If mothers-in-law continue to believe and perpetuate the idea that fatigue is not a symptom of illness, women of reproductive age may also follow suit. Even in situations where a woman does not agree with her mother-in-law, it may be difficult for her to obtain iron supplements against her mother in laws requests.[55] Therefore, in line with the recommendations of other studies, we highlight the importance of shifting mothers-in-law's perceptions of fatigue and anemia, so that perceptions can shift in women as well.[39]

To our knowledge, this is the first study that explores the gaps in iron folic acid distribution for non-pregnant women through an examination of the social context of women living in Odisha, India. The current available health services require that non-pregnant, out-of-school, women seek treatment for their anemia, rather than receive iron supplements unprompted. However, the most recognizable symptom of anemia, fatigue, is not a cue for illness, and thus, does not act as a cue to seek treatment. This study shows that fatigue is not an adequate cue to seek treatment among non-pregnant women resulting in a failure to treat their anemia. We recommend improving the available services to non-pregnant women so that they can obtain iron supplements through

For peer review only - http://bmjopen.bmj.com/site/about/guidelines.xhtml

frontline workers. However, within the current health practice landscape of Odisha, India, we recommend increasing knowledge around the connection between fatigue and anemia among mothers-in-laws, husbands, and women to improve iron supplementation use.

Limitations. Findings from this study provide important contributions to the anemia-reduction literature. However, there are several limitations in this study that must be acknowledged. First, the quantitative methods used in this analysis leave gaps in interpretation. Perceptual maps are a useful tool for depicting the relative conceptualization of items and concepts, but it gives little explanation for the dimension of perception that are depicted. We included a card sorting analysis to attempt to understand the cognitive and affective dimensions. However, there is no method to triangulate these two quantitative methods to understand if the maps are created on these two dimensions. For this reason, we have included a qualitative component, which we have triangulated with the results observed in the quantitative component. We have also used the qualitative component to provide context to the results of the quantitative assessment, despite the fact that they come from different samples. The use of different samples to triangulate may pose as a limitation, but it is worthwhile to note both components share a common sampling procedure and sampling frame.

Second, we note that there are limitations in the way that fatigue can be depicted through our quantitative methods. Participants of the perceptual mapping and card sorting activity were shown a picture of a woman experiencing fatigue, followed by a depiction of the word in the local language. It is possible that the term was too technical to be understood properly. However, the face validity of the cards were evaluated by co-investigators in the field minimize the chances of misinterpretation. Additionally, the

For peer review only - http://bmjopen.bmj.com/site/about/guidelines.xhtml

majority of men and women in Odisha are literate. [56] We also note that the broader goal of the qualitative component was to understand key barriers and facilitators of iron supplementation. As a result, participants were not explicitly asked about their conceptualizations of fatigue. Hence, it is possible that their responses may shift if asked about it directly. Despite this, respondents still revealed a normalization of hard labor among women that corresponds to normalizations of fatigue in the literature. [29] Additionally, it is likely that not all fatigue that women are reporting is related to anemia. Therefore, it may be useful to measure perceptions of changes in fatigue as a cue to seek medical attention, as women may have a baseline level of fatigue that is not related to anemia at all.

For peer review only - http://bmjopen.bmj.com/site/about/guidelines.xhtml

Funding: This study was funded by the Bill and Melinda Gates Foundation under Grant OPP1182519. The funders had no role in study design, data collection and analysis, decision to publish, or preparation of the manuscript.

Author Contributions: HY, ES, and RR made substantial contributions to the conception or design of the work, the analysis, and the interpretation of the work. SP also contributed to the design of the study, and led the data acquisition of data with help from ES and RR. All authors were involved in drafting the work or revising it critically and the final approval of the version to be published. All authors are accountable for all aspects of the work.

Declaration Statement: The authors of this paper have no conflicts of interest to report.

Ethics statement: This study was reviewed and approved by the George Washington University Institutional Review Board (IRB) as well as D-COR Consulting Institutional Ethics Committee (IEC). Data collectors obtained informed consent from all participants in the study.

Data Availability: The data associated with this study can be made available upon request. Anyone who wishes to have access to the de-identified data from the perceptual mapping activity, card sorting activity, or focus group discussions can email the study data manager, Hagere Yilma (corresponding author), at hyilma@gwu.edu. All data is de-identified. Investigators are permitted to access the data for any research questions that have yet to be explored by the study team.

For peer review only

References

1. Benoist, B. d., McLean, E., Egll, I., & Cogswell, M. (2008). Worldwide prevalence of anaemia 1993-2005: WHO global database on anaemia. *Worldwide Prevalence of Anaemia 1993-2005: WHO Global Database on Anaemia.*
2. Strauss, W. E., & Auerbach, M. (2018). Health-related quality of life in patients with iron deficiency anemia: Impact of treatment with intravenous iron. *Patient Related Outcome Measures, 9*, 285.
3. Wouters, H. J., van der Klauw, Melanie M, de Witte, T., Stauder, R., Swinkels, D. W., Wolffenbuttel, B. H., et al. (2019). Association of anemia with health-related quality of life and survival: A large population-based cohort study.*Haematologica, 104*(3), 468-476.
4. Gardner, G. W., Edgerton, V. R., Senewiratne, B., Barnard, R. J., & Ohira, Y. (1977). Physical work capacity and metabolic stress in subjects with iron deficiency anemia. *The American Journal of Clinical Nutrition, 30*(6), 910-917.
5. Allen, L. H. (2000). Anemia and iron deficiency: Effects on pregnancy outcome. *The American Journal of Clinical Nutrition, 71*(5), 1284S.
6. Brabin, B. J., Hakimi, M., & Pelletier, D. (2001). An analysis of anemia and pregnancy-related maternal mortality. *The Journal of Nutrition, 131*(2), 615S.
7. Beard, J. L. (2000). Iron requirements in adolescent females. *The Journal of Nutrition, 130*(2), 442S.
8. Cooke, A. G., McCavit, T. L., Buchanan, G. R., & Powers, J. M. (2017). Iron deficiency anemia in adolescents who present with heavy menstrual bleeding. *Journal of Pediatric and Adolescent Gynecology, 30*(2), 247-250.

For peer review only - http://bmjopen.bmj.com/site/about/guidelines.xhtml

9. Hallberg, L., Högdahl, A., Nilsson, L., & Rybo, G. (1966). Menstrual blood loss and iron deficiency. *Acta Medica Scandinavica, 180*(5), 639-650.

10. Milman, N. (2011). Postpartum anemia I: Definition, prevalence, causes, and consequences. *Annals of Hematology, 90*(11), 1247.

11. XXX (2020). Moving beyond individual barriers and identifying multi-level strategies to reduce anemia in Odisha India. *BMC Public Health, 20*, 1-16.

12. Aurino, E. (2017). Do boys eat better than girls in India? Longitudinal evidence on dietary diversity and food consumption disparities among children and adolescents. *Economics & Human Biology, 25*, 99-111.

13. Bedwell, R. M., Spielvogel, H., Bellido, D., & Vitzthum, V. J. (2017). Factors influencing the use of biomedical health care by rural bolivian anemic women: Structural barriers, reproductive status, gender roles, and concepts of anemia. PloS One, 12(1), e0170475.

14. Jose, S. (2011). Adult undernutrition in India: Is there a huge gender gap? *Economic and Political Weekly*, 95-102.

15. ALIGN. Advancing Learning and Innovation on Gender Norms. (2019). https://www.alignplatform.org/FAQ. Accessed May 26th, 2020.

16. World Health Organization. (2014). *Global nutrition targets 2025: Anaemia policy brief*. Geneva, Switzerland: World Health Organization.

17. Ahmed, F., Khan, M. R., Akhtaruzzaman, M., Karim, R., Williams, G., Torlesse, H., et al. (2010). Long-term intermittent multiple micronutrient supplementation enhances hemoglobin and micronutrient status more than iron folic acid

For peer review only - http://bmjopen.bmj.com/site/about/guidelines.xhtml

supplementation in bangladeshi rural adolescent girls with nutritional anemia. *The Journal of Nutrition, 140*(10), 1879-1886.

18. Vir, S. C., Singh, N., Nigam, A. K., & Jain, R. (2008). Weekly iron and folic acid supplementation with counseling reduces anemia in adolescent girls: A large-scale effectiveness study in uttar pradesh, india. *Food and Nutrition Bulletin, 29*(3), 186-194.

19. World Health Organization. (2016). *Guideline: Daily iron supplementation in adult women and adolescent girls*. Geneva, Switzerland: World Health Organization.

20. International Institute for Population Sciences. (2017). *National family health survey (NFHS-4), 2015-16*. India. Mumbai: IIPS.

21. Bellad, A., Kapil, R., & Gupta, A. (2018). National guidelines for prevention and control of iron deficiency anemia in india. *Indian Journal of Community Health, 30*(01), 89-94.

22. Kumar, A. (1999). National nutritional anaemia control programme in India. *Indian Journal of Public Health, 43*(1), 3-5.

23. Kapil, U., & Bhadoria, A. S. (2014). National iron-plus initiative guidelines for control of iron deficiency anaemia in India, 2013. *Natl Med J India, 27*(1), 27-29.

24. Nguyen, P. H., Scott, S., Avula, R., Tran, L. M., & Menon, P. (2018). Trends and drivers of change in the prevalence of anaemia among 1 million women and children in India, 2006 to 2016. *BMJ Global Health, 3*(5), e001010.

25. Rai, R. K., Fawzi, W. W., Barik, A., & Chowdhury, A. (2018). The burden of iron-deficiency anaemia among women in India: How have iron and folic acid interventions fared? *WHO South-East Asia Journal of Public Health, 7*(1), 18.

For peer review only - http://bmjopen.bmj.com/site/about/guidelines.xhtml

26. UNICEF, & AIIMS. (2018). *Process documentation of national iron plus initiative (NIPI) – towards an anaemia free Odisha, 2016-17*. Bhubaneswar, Odisha: UNICEF.

27. Bager, P. (2014). Fatigue and acute/chronic anaemia. *Dan Med J*, *61*(4), B4824.

28. Sobrero, A., Puglisi, F., Guglielmi, A., Belvedere, O., Aprile, G., Ramello, M., et al. (2001). Fatigue: A main component of anemia symptomatology. Paper presented at the *Seminars in Oncology, 28.* pp. 15-18.

29. Loriol, M. (2017). A sociological stance on fatigue and tiredness: Social inequalities, norms and representations. *Neurophysiologie Clinique/Clinical Neurophysiology, 47*(2), 87-94.

30. Maaita, M., Irtaimeh, T., Altaleb, N., & Ibrahim, R. (2018). Perception of iron deficiency anemia (IDA) and compliance of iron supplements among pregnant women admitted to the labor ward at King Hussein Medical Center, Amman, Jordan. *JRMS, 25*(2), 12-18.

31. Shankar, M., & Reddy, B. (2012). Anaemia in pregnancy still a major cause of morbidity and mortality: insights from Koppal district, Karnataka, India. *Reproductive health matters, 20*(40).

32. Vincent, A., Keerthana, K., Damotharan, K., Newtonraj, A., Bazroy, J., & Manikandan, M. (2017). Health care seeking behaviour of women during pregnancy in rural south India: A qualitative study. *International Journal of Community Medicine and Public Health, 4*(10), 3636-3639.

33. Ball, H. A., Siribaddana, S. H., Sumathipala, A., Kovas, Y., Glozier, N., Rijsdijk, F., ... & Hotopf, M. (2011). Genetic and environmental contributions to the overlap

For peer review only - http://bmjopen.bmj.com/site/about/guidelines.xhtml

between psychological, fatigue and somatic symptoms: a twin study in Sri Lanka. *Twin Research and Human Genetics, 14*(1), 53-63.

34. Ray, C., Weir, W. R. C., Cullen, S., & Phillips, S. (1992). Illness perception and symptom components in chronic fatigue syndrome. *Journal of Psychosomatic Research, 36*(3), 243-256.

35. Bensing, J. M., Hulsman, R. L., & Schreurs, K. M. (1999). Gender differences in fatigue: biopsychosocial factors relating to fatigue in men and women*. Medical care, 37(*10), 1078-1083.

36. Diamond-Smith, N. G., Gupta, M., Kaur, M., & Kumar, R. (2016). Determinants of persistent anemia in poor, urban pregnant women of Chandigarh city, North India: a mixed method approach. Food and nutrition bulletin, 37(2), 132-143.

37. Seymour, S. C. (2010). Environmental change, family adaptations, and child development: Longitudinal research in India. Journal of cross-cultural psychology, 41(4), 578-591.

38. Krupp, K., Placek, C. D., Wilcox, M., Ravi, K., Srinivas, V., Arun, A., & Madhivanan, P. (2018). Financial decision making power is associated with moderate to severe anemia: A prospective cohort study among pregnant women in rural South India. *Midwifery*, *61*, 15-21.

39. Varghese, R., & Roy, M. (2019). Coresidence with mother-in-law and maternal anemia in rural India. *Social Science & Medicine, 226*, 37-46.

40. Adaval, R., Coupey, E., & Narayanan, S. (2015). Direct mapping of consumer perceptions. Paper presented at the *Proceedings of the 1995 Academy of Marketing Science (AMS) Annual Conference,* pp. 255-263.

For peer review only - http://bmjopen.bmj.com/site/about/guidelines.xhtml

41. Hastings, G., Angus, K., & Bryant, C. (Eds.). (2011). *The Sage handbook of social marketing*. Sage.

42. Iacobucci, D., Grisaffe, D., & DeSarbo, W. (2017). Statistical perceptual maps: Using confidence region ellipses to enhance the interpretations of brand positions in multidimensional scaling. *Journal of Marketing Analytics, 5*(3-4), 81-98.

43. Bass, S. B., Jessop, A., Maurer, L., Gashat, M., Al Hajji, M., & Gutierrez, M. (2018). Mapping the barriers and facilitators of HCV treatment initiation in methadone maintenance therapy patients: Implications for intervention development. *Journal of Health Communication, 23*(1), 117-127.

44. Bass, S. B., Wolak, C., Greener, J., Tedaldi, E., Nanavati, A., Ruppert, K., et al. (2016). Using perceptual mapping methods to understand gender differences in perceived barriers and benefits of clinical research participation in urban minority HIV patients. *AIDS Care, 28*(4), 528-536.

45. Pivonka, E., Seymour, J., McKenna, J., Baxter, S. D., & Williams, S. (2011). Development of the behaviorally focused Fruits & Veggies—More Matters public health initiative. Journal of the American Dietetic Association, 111(10), 1570-1577.

46. Ruggieri, D. G., Bass, S. B., Alhajji, M., & Gordon, T. F. (2018). Understanding parents' perceptions of school-based BMI screening and BMI report cards using perceptual mapping: Implications for school nurses. *The Journal of School Nursing*, 1059840518789243.

47. Van Voorhees, B. W., Watson, N., Bridges, J. F., Fogel, J., Galas, J., Kramer, C., ... & Landsback, J. (2010). Development and pilot study of a marketing strategy for primary care/Internet–based depression prevention intervention for adolescents (The

For peer review only - http://bmjopen.bmj.com/site/about/guidelines.xhtml

CATCH-IT Intervention). Primary care companion to the Journal of clinical psychiatry, 12(3).

48. XXX (2018). The RANI Project: A socio-normative intervention to reduce anemia in Odisha, India: A formative research protocol. *Gates open research*, 2.

49. Nguyen, P. H., Sanghvi, T., Kim, S. S., Tran, L. M., Afsana, K., Mahmud, Z., et al. (2017). Factors influencing maternal nutrition practices in a large scale maternal, newborn and child health program in Bangladesh. *PLoS One, 12*(7), e0179873.

50. Tinago, C. B., Annang Ingram, L., Blake, C. E., & Frongillo, E. A. (2017). Individual and structural environmental influences on utilization of iron and folic acid supplementation among pregnant women in Harare, Zimbabwe. *Maternal & child nutrition*, 13(3), e12350.

51. Bijmolt, T. H., & Wedel, M. (1999). A comparison of multidimensional scaling methods for perceptual mapping. *Journal of Marketing Research, 36*(2), 277-285.

52. Guest, G., MacQueen, K. M., & Namey, E. E. (2011). *Applied thematic analysis*. Sage Publications.

53. Das, A., & Sarkar, M. (2014). Pregnancy-related health information-seeking behaviors among rural pregnant women in india: Validating the wilson model in the indian context. *The Yale Journal of Biology and Medicine, 87*(3), 251.

54. Ramachandran, D., Canny, J., Das, P. D., & Cutrell, E. (2010). Mobile-izing health workers in rural india. Paper presented at the *Proceedings of the SIGCHI Conference on Human Factors in Computing Systems,* pp. 1889-1898.

55. Allendorf, K. (2013). Going nuclear? family structure and young women's health in india, 1992–2006. *Demography, 50*(3), 853-880.

Only

For peer review only - http://bmjopen.bmj.com/site/about/guidelines.xhtml

56. Government of India. Census of India: 2011. New Delhi: Office of the Registrar General & Census Commissioner; 2012.

For peer review only

Table 1. Demographic Information- Perceptual Mapping and Card Sorting

	**Men** (n=30)	**WRA** (n=30)	**MILs** (n=30)
	M (SD)	***M (SD)***	***M (SD)***
**Age**	34.67 (6.00)	27.90 (4.76)	55.87 (7.58)
	**n (%)**	**n (%)**	**n (%)**
**School**			
None	3 (10)	4 (13.3)	21 (70.0)
Up to Primary	12 (40)	8 (26.7)	6 (20.0)
Up to Secondary	6 (20)	7 (23.3)	2 (6.7)
Up to High Secondary	5 (16.6)	10 (33.4)	1 (3.3)
Up to Tertiary	4 (13.3)	1 (3.3)	0 (0)
**Married**	30 (100)	30 (100)	29 (96.7)[a]
**Religion-Hindu**	100 (100)	100 (100)	100 (100)
**Caste**			
Scheduled Caste/ Tribe	18 (60.0)	16 (53.3)	17 (56.6)
Other Backwards Caste	11 (36.7)	12 (40.0)	13 (43.3)
Other Caste	1 (3.3)	2 (6.7)	0 (0)
**Children**			
None	0 (0)	3 (10)	0 (0)
1	4 (13.3)	7 (23.3)	3 (10.0)
2	12 (40.0)	10 (33.3)	3 (10.0)

For peer review only - http://bmjopen.bmj.com/site/about/guidelines.xhtml

	Men	WRA	MILs
3	10 (33.3)	7 (23.3)	11 (36.7)
4 or more	4 (13.3)	3 (10.0)	13 (43.3)
**Ever taken IFA**	4 (13.3)	26 (86.7)	7 (23.3)
**Currently taking IFA**	0 (0)	3 (10.0)	2 (9.1)
**Diagnoses of anemia ever**	1 (3.3)	16 (53.3)	4 (13.3)
**Currently anemic?**	5 (21.7)	4 (14.3)	3 (13.0)

Note: Under “School”, “I” indicates incomplete and “C” indicates complete; [a] The one mother-in-law who wasn’t married was a widow. WRA = women of reproductive age; MILs = Mothers-in-law; IFA = iron folic acid.

*p < 0.05, **p < 0.01, ***p < 0.001

For peer review only

Table 2. Importance to Health and Dislike Rating Across Participant Category

		WRA	Men	MILs	
Item	Rating Parameter	*M* (SD)	*M* (SD)	*M* (SD)	*F*
IFA pill	Importance	1.67 (0.68)	1.87 (0.35)	1.73 (0.52)	2.39
	Dislike	0.53 (0.73)	0.20 (0.48)	0.47 (0.73)	2.15
Clinic	Importance	1.80 (0.48)	1.93 (0.25)	1.83 (0.38)	0.98
	Dislike	0.23 (0.50)	0.20 (0.41)	0.23 (0.50)	0.05
Kiosk	Importance	1.77 (0.50)	1.93 (0.25)	1.87 (0.35)	1.44
	Dislike	0.27 (0.58)	0.17 (0.38)	0.13 (0.43)	0.64
Self-Help Groups	Importance	1.37 (0.72)	1.27 (0.74)	1.57 (0.68)	1.38
	Dislike	0.30 (0.47)[a]	0.73 (0.74)[b]	0.50 (0.68)[a,b]	3.44*
Traditional healer	Importance	0.80 (0.76)	1.23 (0.82)	1.03 (0.67)	2.50
	Dislike	1.07 (0.78)	0.97 (0.85)	1.07 (0.69)	0.17
Physician	Importance	1.80 (0.55)	2.00 (0)	1.90 (0.31)	2.27
	Dislike	0.20 (0.41)	0.10 (0.31)	0.20 (0.48)	0.61
Fatigue	Importance	0.77 (0.73)[a]	0.80 (0.61)[a]	0.17 (0.53)[b]	9.66***
	Dislike	1.47 (0.68)[a]	1.23 (0.73)[a]	1.87 (0.35)[b]	8.29***
Prenatal care	Importance	1.43 (0.73)	1.40 (0.67)	1.67 (0.61)	1.40
	Dislike	0.70 (0.89)	0.57 (0.50)	0.33 (0.66)	2.12
Green veggies	Importance	1.63 (0.62)	1.63 (0.49)	1.80 (0.41)	1.06
	Dislike	0.37 (0.61)	0.30 (0.60)	0.13 (0.35)	1.52
Meat	Importance	1.23 (0.68)	0.97 (0.76)	1.37 (0.61)	2.62
	Dislike	0.77 (0.73)	0.90 (0.76)	0.60 (0.62)	1.36

For peer review only - http://bmjopen.bmj.com/site/about/guidelines.xhtml

Pregnancy	Importance	1.13 (0.82)	1.23 (0.63)	1.57 (0.68)	3.04
	Dislike	0.80 (0.85)[a]	0.80 (0.48)[a]	0.30 (0.60)[b]	5.74**
Stomach-ache	Importance	0.63 (0.67)[a]	0.87 (0.68)[a]	0.30 (0.60)[b]	5.76**
	Dislike	1.47 (0.68)[a]	1.17 (0.65)[b]	1.83 (0.46)[c]	9.15***

Note: The letters a and b denote homogenous subsets yielded from the post-hoc analysis when omnibus significance was detected. WRA = women of reproductive age; MILs = Mothers-in-law; IFA = iron folic acid.

*p < 0.05, **p < 0.01, ***p < 0.001

For peer review only

Figure Captions.

Figure 1. Perceptual Map derived for Women of Reproductive Age. Dissimilarity plot across two-dimensions (corresponding to the x and y axis) as displayed by SPSS v. 25; plot point labels have been edited for reader clarity.

Figure 2. Perceptual Map derived for Men. Dissimilarity plot across two-dimensions (corresponding to the x and y axis) as displayed by SPSS v. 25; plot point labels have been edited for reader clarity.

Figure 3. Perceptual Map derived for Mothers-in-Law. Dissimilarity plot across two-dimensions (corresponding to the x and y axis) as displayed by SPSS v. 25; plot point labels have been edited for reader clarity.

For peer review only

For peer review only - http://bmjopen.bmj.com/site/about/guidelines.xhtml

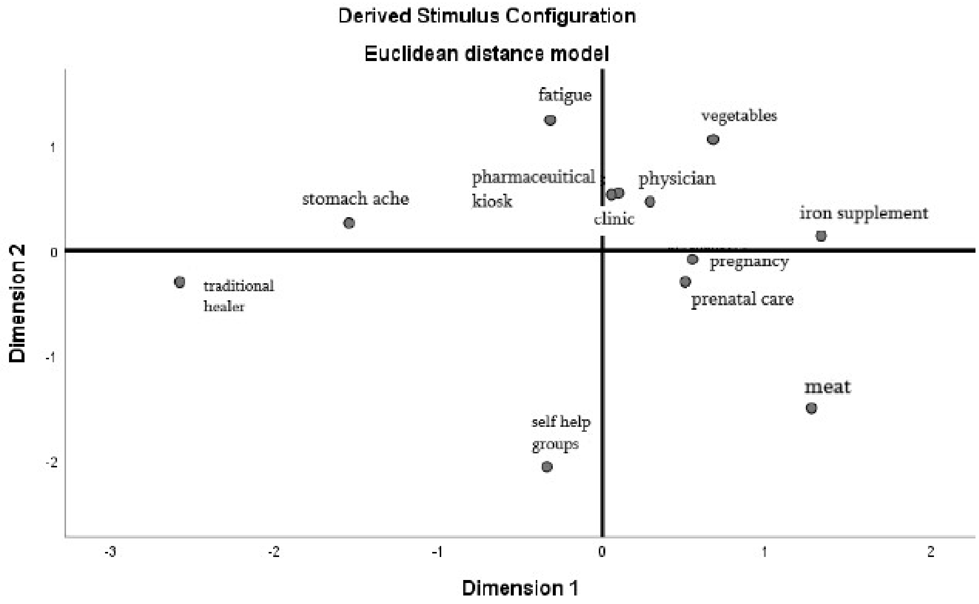

Figure 1. Perceptual Map derived for Women of Reproductive Age. Dissimilarity plot across two-dimensions (corresponding to the x and y axis) as displayed by SPSS v. 25; plot point labels have been edited for reader clarity.

171x107mm (96 x 96 DPI)

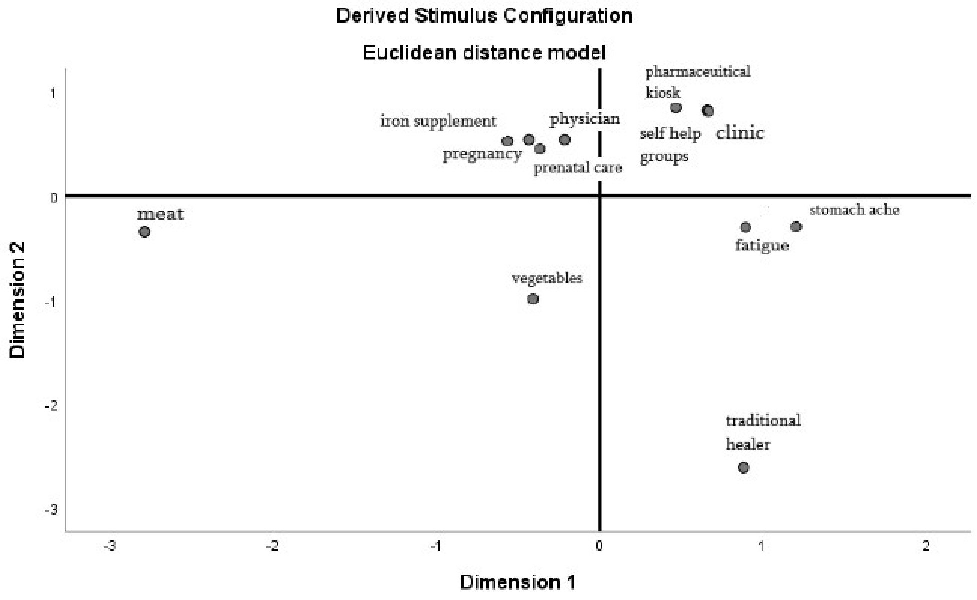

Figure 2. Perceptual Map derived for Males. Dissimilarity plot across two-dimensions (corresponding to the x and y axis) as displayed by SPSS v. 25; plot point labels have been edited for reader clarity.

175x109mm (96 x 96 DPI)

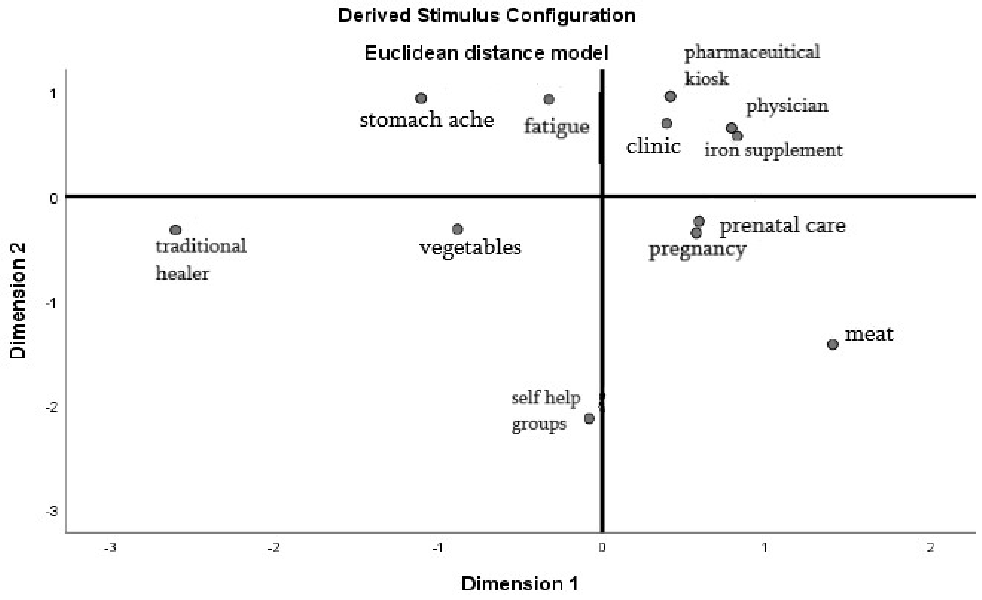

Figure 2. Perceptual Map derived for Males. Dissimilarity plot across two-dimensions (corresponding to the x and y axis) as displayed by SPSS v. 25; plot point labels have been edited for reader clarity.
Figure 3. Perceptual Map derived for Mothers-in-Law. Dissimilarity plot across two-dimensions (corresponding to the x and y axis) as displayed by SPSS v. 25; plot point labels have been edited for reader clarity.

177x111mm (96 x 96 DPI)

Supplement 1

PERCEPTUAL MAPPING AND CARD SORTING QUESTIONNAIRE

FOR RESEARCH TEAM MEMBER USE ONLY/ କେବଳ ଅନୁସନ୍ଧାନକାରୀ ଦଳର ସଦସ୍ୟଙ୍କ ବ୍ୟବହାର ନିମନ୍ତେ:		
BLOCK	Which Block is the respondent from/ଉତ୍ତରଦାତା କେଉଁ ବ୍ଲକରୁ?	....
VILLAGE	Which village is the respondent from/ଉତ୍ତରଦାତା କେଉଁ ଗ୍ରାମରୁ?	
PARTICIPATION	The respondent is participating in a...<br>ଉତ୍ତରଦାତାଜଣକ କେଉଁଥିରେ ଭାଗ ନେଉଛନ୍ତି	Focus group discussion........................1<br>ଦଳଗତ ଆଲୋଚନା..1<br>Key informant interview.........................2<br>ପ୍ରମୁଖ ସୂଚନାଦାତାଙ୍କ ସହିତ ସାକ୍ଷାତକାର ....2<br>Perceptual Mapping -------------------3<br>ଧାରଣାତ୍ମକ ମାନଚିତ୍ରଣ --------------------------3
CODE_PM	What is the code number of the Perceptual Mapping?<br>ଏହି ଧାରଣାତ୍ମକ ମାନଚିତ୍ରଣର କୋଡ କଣ?	Code number/ କୋଡ ନମ୍ବର୍ __________<br>PM1<br>PM2<br>PM3<br>PM4<br>PM5<br>PM6<br>PM7<br>PM8<br>PM9

For peer review only - http://bmjopen.bmj.com/site/about/guidelines.xhtml

		PM10<br>PM11<br>PM12
PARTICIPANT_PM	Participant category of Perceptual Mapping ଧାରଣାତ୍ମକ ମାନଚିତ୍ରଣରେ ଅଂଶଗ୍ରହଣ କରିଥିବା ଉତ୍ତରଦାତାଙ୍କ ପ୍ରକାର	Males 18 – 42 years old --------------------1<br>ପୁରୁଷ (୧୮-୪୨) (ବିବାହିତ) ------------------------1<br>WRA 15 – 35 years old ---------------------2<br>ପ୍ରଜନନ ବୟସ (୧୫-୩୫) ମଧ୍ୟରେ ଥିବା ମହିଳା ------2<br>Mothers in law – any age -----------------3<br>ଶାଶୁ (ସ୍ୱାମୀଙ୍କ ମା) ---------------------------------3
CODE_NUMBER_PM	Participant code number of the Perceptual Mapping ଧାରଣାତ୍ମକ ମାନଚିତ୍ରଣରେ ଭାଗ ନେଇଥିବା ସଦସ୍ୟଙ୍କ କୋଡ଼	
PATRICIPATION_DATE	On what date did the respondent participate/କେଉଁ ତାରିଖରେ ଉତ୍ତରଦାତା ଅଂଶଗ୍ରହଣ କରିଥିଲେ?	DD/MM: ______________________<br>ତାରିଖ / ମାସ ____________________

Thank you for participating in this study. I want to remind you that there are no right or wrong answers to these questions. We just want to know what your opinions are. Before we ask for your opinions, please tell us a few things about yourself/ଆପଣ ଏହି ଅଧ୍ୟୟନରେ ଅଂଶଗ୍ରହଣ କରିଥିବାରୁ ଧନ୍ୟବାଦ । ମୁଁ ଆପଣଙ୍କୁ ମନେ ପକେଇଦେବାକୁ ଚାହେଁ ଯେ ଏହି ସବୁ ପ୍ରଶ୍ନର ଠିକ କିମ୍ବା ଭୁଲ ଉତ୍ତର ନାହିଁ । ଆମେ କେବଳ ଆପଣଙ୍କ ମତାମତ ଗୁଡିକ ଜାଣିବାକୁ ଚାହୁଁଛୁ, ଦୟାକରି ଆପଣଙ୍କ ବିଷୟରେ ଆମକୁ କିଛି କୁହନ୍ତୁ ।

Section/ବିଭାଗ 1

Demographic Questionnaire/ଜନସଂଖ୍ୟା ସମ୍ବନ୍ଧୀୟ ପ୍ରଶ୍ନାବଳୀ

1	How old are you?<br>ଆପଣଙ୍କର ବୟସ କେତେ?	Age: ________________ (Unknown = -99)<br>ବୟସ: ------------------------ (ଜାଣି ନାହାନ୍ତି = -୯୯)
2	What is your sex?	Female/ମହିଳା 1

For peer review only - http://bmjopen.bmj.com/site/about/guidelines.xhtml

	ଆପଣଙ୍କର ଲିଙ୍ଗ କଣ?	Male/ପୁରୁଷ....................................2<br>No response/କୌଣସି ଉତ୍ତର ନାହିଁ..._......3
3	What is the highest level of school you have attended?<br>ଆପଣଙ୍କର ସର୍ବୋଚ୍ଚ ଶିକ୍ଷାଗତ ଯୋଗ୍ୟତା କଣ?	None/କିଛିନାହିଁ - 0<br>Primary school (incomplete)/ପ୍ରାଥମିକ ବିଦ୍ୟାଳୟ (ଅସମ୍ପୂର୍ଣ) - 1<br>Primary school (complete) /ପ୍ରାଥମିକ ବିଦ୍ୟାଳୟ (ସମ୍ପୂର୍ଣ) - 2<br>Secondary school (incomplete) /ମାଧ୍ୟମିକ ବିଦ୍ୟାଳୟ (ଅସମ୍ପୂର୍ଣ) - 3<br>Secondary school (complete) /ମାଧ୍ୟମିକ ବିଦ୍ୟାଳୟ (ସମ୍ପୂର୍ଣ) - 4<br>High Secondary/ Senior Secondary school (incomplete) /ଉଚ୍ଚ ମାଧ୍ୟମିକ ବିଦ୍ୟାଳୟ (ଅସମ୍ପୂର୍ଣ) - 5<br>High Secondary/ Senior school (complete) /ଉଚ୍ଚ ମାଧ୍ୟମିକ ବିଦ୍ୟାଳୟ (ସମ୍ପୂର୍ଣ) - 6<br>Tertiary/College/University (incomplete)/ମହାବିଦ୍ୟାଳୟ/ବିଶ୍ୱବିଦ୍ୟାଳୟ (ଅସମ୍ପୂର୍ଣ) - 7<br>Tertiary/College/University (complete)/ମହାବିଦ୍ୟାଳୟ/ବିଶ୍ୱବିଦ୍ୟାଳୟ (ସମ୍ପୂର୍ଣ) - 8<br>Other (Specify)/ଅନ୍ୟାନ୍ୟ (ଦର୍ଶାନ୍ତୁ):- 88<br>No response/କୌଣସି ଉତ୍ତରନାହିଁ...........-99
4	Are you currently married or living together with a partner as if married?<br>ଆପଣ ବିବାହିତ କି ବା ଜଣେ ସାଥିଙ୍କ ସହିତ ବିବାହିତ ଭଳି ମିଶିକରି ରୁହନ୍ତି କି ?	Yes, currently married/ହଁ, ବିବାହିତ...........1<br>Yes, living with a partner/ହଁ, ଜଣେ ସାଥୀଙ୍କ ସହିତ ରହୁଛି ....2<br>No/ନାହିଁ...3<br>No response/ କୌଣସି ଉତ୍ତର ନାହିଁ...........-99
4a	IF NO: Are you divorced, separated, widowed, or never married or in a union?<br><br>(Skip if married or living with a partner.)	Never married or in a union/କେବେ ବିବାହ କରିନାହାନ୍ତି କିମ୍ବା କୌଣସି ସାଥୀଙ୍କ ସହିତ ରଖୁନାହାଁନ୍ତ...1<br>Divorced/separated/ଛାଡପତ୍ର/ଅଲଗା ରହୁଛନ୍ତି...2<br>Widow/widower / ବିଧବା / ବିପତି.............3

For peer review only - http://bmjopen.bmj.com/site/about/guidelines.xhtml

	ଯଦି ନା : ଆପଣ କଣ ଛାଡପତ୍ର ନେଇଛନ୍ତି, ଅଲଗା ରହୁଛନ୍ତି, ବିଧବା ବା କେବେ ବିବାହ କରିନାହାନ୍ତି କିମ୍ବା କୌଣସି ସାଥୀଙ୍କ ସହିତ ନାହାନ୍ତି ?<br>(ପଚାରନ୍ତୁ ନାହିଁ, ଯଦି ବିବାହିତ କିମ୍ବା ସାଥି ସହିତ ରହୁଛନ୍ତି)	No response/ କୌଣସି ଉତ୍ତର ନାହିଁ.............-99
5	What is your religion?<br>ଆପଣଙ୍କର ଧର୍ମ କଣ ?	Hindu/ହିନ୍ଦୁ ....1<br>Muslim/ମୁସଲିମ ....2<br>Christian/ଖ୍ରୀଷ୍ଟିୟାନ..............................3<br>Sikh/ଶିଖ.......................................4<br>Buddhist/ବୁଦ୍ଧ...................................5<br>Jain/ଜୈନ..6<br>Other/ଅନ୍ୟାନ୍ୟ..................................7<br>No response/ କୌଣସି ଉତ୍ତର ନାହିଁ.........-99
CASTE	What is your caste ?<br>ଆପଣଙ୍କର ଜାତି କଣ ?	Scheduled Caste ଅନୁସୂଚିତ ଜାତି - 1<br>Scheduled Tribe ଅନୁସୂଚିତ ଜନଜାତି - 2<br>OBC ଅନ୍ୟାନ୍ୟ ପଛୁଆ ବର୍ଗର ଜାତି - 3<br>Other Caste ଅନ୍ୟାନ୍ୟ ଜାତି - 4<br>No response/ କୌଣସି ଉତ୍ତର ନାହିଁ.........-99
6	How many children do you have?<br>ଆପଣଙ୍କର କେତୋଟି ସନ୍ତାନ ଅଛନ୍ତି ?	None/କିଛିନାହିଁ....................................0<br>One/ଗୋଟିଏ....................................1<br>Two/ଦୁଇଟି...2<br>Three/ତିନୋଟି....................................3<br>Four/ଚାରିଟି.......................................4<br>Five or more/ପାଞ୍ଚ କିମ୍ବା ଅଧିକ................5<br>No response// କୌଣସି ଉତ୍ତର ନାହିଁ..........-99
7	How often do you watch television?<br>ଆପଣ କେତେ ବ୍ୟବଧାନରେ ଟିଭି ଦେଖନ୍ତି ?	Not at all/କେବେ ନାହିଁ...............................0<br>Less than once a week/ସପ୍ତାହରେ ଥରକରୁ କମ୍...1<br>At least once a week/ ଅତିକମ୍ ରେ ସପ୍ତାହକୁ ଥରେ 2

For peer review only - http://bmjopen.bmj.com/site/about/guidelines.xhtml

		Almost Every Day/ପ୍ରାୟ ପ୍ରତ୍ୟକ ଦିନ............3
7a	On an average day, for how long do you watch television?<br>ଗୋଟିଏ ଦିନରେ, ପାଖାପାଖି କେତେ ସମୟ ଟିଭି ଦେଖନ୍ତି ?	______Hours per day/ OR ____ minutes per day<br>ଦିନରେ .... ଘଣ୍ଟା କିମ୍ବା ଦିନରେ .... ମିନିଟ୍
8	How often do you listen to the radio?<br>ଆପଣ କେତେଥର ରେଡିଓ ଶୁଣନ୍ତି ?	Not at all/କେବେ ନାହିଁ...............................0<br>Less than once a week/ ସପ୍ତାହରେ ଥରକରୁ କମ୍...1<br>At least once a week/ ଅତିକମ୍ ରେ ସପ୍ତାହକୁ ଥରେ ....2<br>Almost Every Day/ ପ୍ରାୟ ସବୁଦିନ.................3
8a	On an average day, for how long do you listen to the radio?<br>ଗୋଟିଏ ଦିନରେ ପାଖାପାଖି କେତେ ସମୟ ରେଡିଓ ଶୁଣନ୍ତି?	______Hours per day OR ____ minutes per day<br>ଦିନରେ .... ଘଣ୍ଟା କିମ୍ବା ଦିନରେ .... ମିନିଟ
9.0	How often do you talk on a mobile phone?<br>ଆପଣ କେତେଥର ମୋବାଇଲରେ କଥା ହୁଅନ୍ତି?	Not at all/କେବେ ନାହିଁ...............................0<br>Less than once a week/ ସପ୍ତାହରେ ଥରକରୁ କମ୍...1<br>At least once a week/ ଅତିକମ୍ ରେ ସପ୍ତାହକୁ ଥରେ ....2<br>Almost Every Day/ପ୍ରାୟ ସବୁଦିନ.................3
9	Do you use a smart phone?<br>ଆପଣ ସ୍ମାର୍ଟ ଫୋନ୍ ବ୍ୟବହାର କରନ୍ତି କି ?	Yes/ହଁ...1<br>No/ନା...0
9a	On an average day, for how long do you talk on a mobile phone?<br>ଗୋଟିଏ ଦିନରେ, ପାଖାପାଖି କେତେ ସମୟ ମୋବାଇଲରେ କଥା ହୁଅନ୍ତି?	______Hours per day OR ____ minutes per day<br>ଦିନରେ .... ଘଣ୍ଟା କିମ୍ବା ଦିନରେ .... ମିନିଟ
10	How often do you read the newspaper?<br>ଆପଣ କେତେଥର ଖବରକାଗଜ ପଢନ୍ତି?	Not at all/କେବେ ନାହିଁ...............................0<br>Less than once a week/ ସପ୍ତାହରେ ଥରକରୁ କମ୍...1<br>At least once a week/ ଅତିକମ୍ ରେ ସପ୍ତାହକୁ ଥରେ ....2<br>Almost Every Day/ପ୍ରାୟ ସବୁଦିନ.................3

For peer review only - http://bmjopen.bmj.com/site/about/guidelines.xhtml

10a	On an average day, for how long do you read the newspaper?<br>ଗୋଟିଏ ଦିନରେ, ପାଖାପାଖି କେତେ ସମୟ ଖବରକାଗଜ ପଢନ୍ତି?	______Hours per day OR ____ minutes per day<br>ଦିନରେ .... ଘଣ୍ଟା କିମ୍ବା ଦିନରେ .... ମିନିଟ
11	Have you ever attended a self-help group meeting?<br>ଆପଣ କେବେ ସ୍ୱୟଂ ସହାୟକ ଦଳର ବୈଠକରେ ଯୋଗ ଦେଇଛନ୍ତି କି ?	Yes/ହଁ...1<br>No/ନା...0
11a	Are you currently a part of a self-help group/ଆପଣ ବର୍ତ୍ତମାନ ସ୍ୱୟଂ ସହାୟକ ଦଳର ଜଣେ /ସଦସ୍ୟ କି?	Yes/ହଁ...1<br>No/ନା...0
12	Have you ever taken iron & folic acid supplements (IFA)/ଆପଣ କେବେ ବି ଆଇରନ ଏବଂ ଫଲିକ ଏସିଡର ପରିପୂରକ ଖାଇଛନ୍ତି କି ?	Yes/ହଁ...1<br>No/ନା...0
12a	Are you currently taking iron & folic acid supplements (IFA)/ଆପଣ ବର୍ତ୍ତମାନ ଆଇରନ ଏବଂ ଫଲିକ ଏସିଡର ପରିପୂରକ ଖାଉଛନ୍ତି କି ?	Yes/ହଁ...1<br>No/ନା...0
13	Has a doctor or healthcare provider ever told you that you have anemia/ଆପଣଙ୍କର ରକ୍ତହୀନତା ଅଛି ବୋଲି ଆପଣଙ୍କୁ ଡାକ୍ତର କିମ୍ବା କୌଣସି ସ୍ୱାସ୍ଥ୍ୟ ପ୍ରଦାନକାରୀ କେବେ କହିଛନ୍ତି କି ?	Yes/ହଁ...1<br>No/ନା...0
13a	Do you currently have anemia/ବର୍ତ୍ତମାନ ଆପଣଙ୍କର ରକ୍ତହୀନତା ରହିଛି କି ?	Yes/ହଁ...1<br>No/ନା...0

For peer review only - http://bmjopen.bmj.com/site/about/guidelines.xhtml

Section/ବିଭାଗ 2

I am now going to show you two cards at a time. One card has the picture of one object and the second card has the picture of a second object. Please tell me how similar the second card is to the first. To make this a bit easier, I will show you the second card and first ask you whether it is similar or different. If you say “similar,” then I will ask you whether it is somewhat similar or very similar; if you say “different,” then I will ask you whether it is somewhat different or very different.

ଏବେ ମୁଁ ଆପଣଙ୍କୁ ଏକ ସମୟରେ ଦୁଇଟି କାର୍ଡ ଦେଖାଇବାକୁ ଯାଉଛି | ଗୋଟିଏ କାର୍ଡରେ ଗୋଟିଏ ବିଷୟରେ ଛବି ଏବଂ ଦ୍ଵିତୀୟ କାର୍ଡରେ ଦ୍ଵିତୀୟ ବିଷୟରେ ଛବି ରହିଛି । ଦୟାକରି ମୋତେ କୁହନ୍ତୁ ଦ୍ଵିତୀୟ କାର୍ଡଟି ପ୍ରଥମ କାର୍ଡ ସହିତ କେତେ ସମାନ ଅଛି । ଆମେ ଏହାକୁ ଆଉ ଟିକେ ସହଜ କରିବା ପାଇଁ ମୁଁ ଆପଣଙ୍କୁ ଦ୍ଵିତୀୟ କାର୍ଡଟି ଦେଖାଇବି ଏବଂ ପ୍ରଥମେ ପଚାରିବି ଏହା ସମାନ କିମ୍ବା ଅଲଗା । ଯଦି ଆପଣ ସମାନ କହନ୍ତି ତେବେ କିଛି ସମାନ ନା ସମ୍ପୂର୍ଣ ସମାନ ବୋଲି ମୁଁ ପଚାରିବି; ଯଦି ଆପଣ ଅଲଗା କହନ୍ତି ତେବେ କିଛି ଅଲଗା ନା ସମ୍ପୂର୍ଣ ଅଲଗା ବୋଲି ମୁଁ ପଚାରିବି ।

[Show respondent each card pair: 1 versus 2, 3, 4,..... 16; 2 versus 3, 4, 5, ... 16; etc.]

[ଉତ୍ତରଦାତାଙ୍କୁ ପ୍ରତ୍ୟେକ କାର୍ଡର ଯୋଡି ଦେଖାନ୍ତୁ: 1 ସହିତ 2, 3, 4 ... 16; 2 ସହିତ 3, 4, 5, ... 16 ଇତ୍ୟାଦି]

1. IFA Pill/ଆଇରନ ବଟିକା
2. Clinic/କ୍ଲିନିକ
3. Kiosk
4. Self-help group/ସ୍ଵୟଂ ସହାୟକ ଦଳ
5. Traditional healer/ ପାରମ୍ପରିକ / ଚିକିତ୍ସକ / ବୈଦ୍ୟ
6. Physician/ ଚିକିତ୍ସକ / ଡାକ୍ତର
7. Fatigue/କ୍ଳାନ୍ତି
8. Prenatal care/ ଗର୍ଭାବସ୍ଥା ସମୟରେ ଯତ୍ନ
9. Green vegetables/ସବୁଜ ପନିପରିବା
10. Meat/ମାଂସ
11. Pregnancy/ଗର୍ଭାବସ୍ଥା
12. Stomach ache/ପେଟ କାଟିବା

[For each pair, first ask: Is XXXX similar or different from YYYY/ପ୍ରତ୍ୟେକ ଯୋଡି ପାଇଁ ପ୍ରଥମେ ପଚାରନ୍ତୁ XXXX ସମାନ କିମ୍ବା YYYY ଠାରୁ ଅଲଗା ।

For peer review only - http://bmjopen.bmj.com/site/about/guidelines.xhtml

If Respondent says, “similar,” then ask: Would you say just a bit similar or very similar?

ଯଦି ଉତ୍ତରଦାତା ସମାନ କହନ୍ତି ତେବେ ପଚାରନ୍ତୁ ଆପଣ କିଛି ସମାନ କହୁଛନ୍ତି କିମ୍ବା ସମ୍ପୂର୍ଣ୍ଣ ସମାନ କହୁଛନ୍ତି?

If Respondent says, “different,” then ask: Would you say just a bit different or very different?

ଯଦି ଉତ୍ତରଦାତା ଅଲଗା କହନ୍ତି ତେବେ ପଚାରନ୍ତୁ ଆପଣ କିଛି ଅଲଗା କହୁଛନ୍ତି କିମ୍ବା ସମ୍ପୂର୍ଣ୍ଣ ଅଲଗା କହୁଛନ୍ତି?]

For peer review only

For peer review only - http://bmjopen.bmj.com/site/about/guidelines.xhtml

Card 1	Card 2	1 = very different/ସମ୍ପୂର୍ଣ୍ଣ ଅଲଗା<br>2 = somewhat differentକିଛି ଅଲଗା<br>3 = somewhat similarକିଛି ସମାନ<br>4 = very similarସମ୍ପୂର୍ଣ୍ଣ ସମାନ
1. IFA pill/ଆଇରନ ବଟିକା	2	
	3	
	4	
	5	
	6	
	7	
	8	
	9	
	10	
	11	
	12	
2. Clinic କ୍ଲିନିକ	3	
	4	
	5	
	6	
	7	
	8	
	9	
	10	
	11	
	12	

Card 1	Card 2	1 = very different/ ସମ୍ପୂର୍ଣ୍ଣ ଅଲଗା<br>2 = somewhat different କିଛି ଅଲଗା<br>3 = somewhat similar କିଛି ସମାନ<br>4 = very similar ସମ୍ପୂର୍ଣ୍ଣ ସମାନ
3. Kiosk	4	
	5	
	6	
	7	
	8	
	9	
	10	
	11	
	12	
4. SHG ସ୍ୱୟଂ ସହାୟକ ଦଳ	5	
	6	
	7	
	8	
	9	
	10	
	11	
	12	

Card 1	Card 2	1 = very different/ସମ୍ପୂର୍ଣ୍ଣ ଅଲଗା<br>2 = somewhat differentକିଛି ଅଲଗା<br>3 = somewhat similar କିଛି ସମାନ<br>4 = very similar ସମ୍ପୂର୍ଣ୍ଣ ସମାନ
5. Traditional healer ପାରମ୍ପାରିକ ଚିକିତ୍ସକ / ବୈଦ୍ୟ	6	
	7	
	8	
	9	
	10	
	11	
	12	
6. Physician ଡାକ୍ତର	7	
	8	
	9	
	10	
	11	
	12	
7. Fatigue କ୍ଲାନ୍ତି	8	
	9	
	10	
	11	
	12	

For peer review only

For peer review only - http://bmjopen.bmj.com/site/about/guidelines.xhtml

Card 1	Card 2	1 = very different/ ସମ୍ପୂର୍ଣ୍ଣ ଅଲଗା<br>2 = somewhat different କିଛି ଅଲଗା<br>3 = somewhat similar କିଛି ସମାନ<br>4 = very similar ସମ୍ପୂର୍ଣ୍ଣ ସମାନ
8. Prenatal care ଗର୍ଭାବସ୍ଥା ସମୟରେ ଯତ୍ନ	9	
	10	
	11	
	12	
9. Green vegetables ସବୁଜ ପନିପରିବା	10	
	11	
	12	
10. Meat ମାଂସ	11	
	12	
11. Pregnancy/ଗର୍ଭାବସ୍ଥା	12	

For peer review only

For peer review only - http://bmjopen.bmj.com/site/about/guidelines.xhtml

Section/ବିଭାଗ 3

Importance for Health and Well-Being/ସ୍ୱାସ୍ଥ୍ୟ ଏବଂ ଭଲ ରହିବା ପାଇଁ ମହତ୍ତ୍ୱ

I will now show you the same 12 cards. Please put these cards in three different piles. In the first pile, put all the cards that you think are very important for your health and well-being. In the second pile, put all the cards that you think are just a little bit important for your health and well-being. In the third pile, put all the cards that you think are not important at all for your health and well-being. [Do the exercise and write response in the first table. Then shuffle the cards.]

ଏବେ ମୁଁ ଆପଣଙ୍କୁ ୧୨ଟି କାର୍ଡ ଦେଖାଇବି । ଏହି କାର୍ଡଗୁଡିକ ତିନୋଟି ଅଲଗା ଗଦାରେ ରଖନ୍ତୁ | ଆପଣ ଯେଉଁ ସବୁକାର୍ଡ ଗୁଡିକ ଭାବୁଛନ୍ତି ଆପଣଙ୍କର ସ୍ୱାସ୍ଥ୍ୟ ଏବଂ ଭଲରହିବା ପାଇଁ ଜରୁରୀ ସେଗୁଡିକ ପ୍ରଥମ ଗଦାରେ ରଖନ୍ତୁ | ଯେଉଁସବୁ କାର୍ଡଗୁଡିକ ଆପଣଙ୍କ ସ୍ୱାସ୍ଥ୍ୟ ଏବଂ ଭଲ ରହିବା ପାଇଁ କମ ଜରୁରୀ ସେଗୁଡିକ ଦ୍ୱିତୀୟ ଗଦାରେ ରଖନ୍ତୁ | ସେହିପରି, ଯେଉଁସବୁ କାର୍ଡଗୁଡିକ ଆପଣଙ୍କ ସ୍ୱାସ୍ଥ୍ୟ ଏବଂ ଭଲରହିବା ପାଇଁ ଜମା ଜରୁରୀ ନୁହେଁ, ସେଗୁଡିକ ତୃତୀୟ ଗଦାରେ ଅଛି ? (ଏହି ଖେଳଟିକୁ କରାନ୍ତୁ ଏବଂ ଉତ୍ତରକୁ ପ୍ରଥମ ଟେବୁଲରେ ଲେଖନ୍ତୁ | ତାହାପରେ କାର୍ଡଗୁଡିକ ମିଶାଇଦିଅନ୍ତୁ |)

Easy/ସହଜ

This time please think about how easy it is to get (or to use or to do) each item shown in the card. If you think the item is very easy to get (or to use or to do), put it in the first pile. If you think the item is sometimes easy and sometimes difficult to get (or to use or to do), put it in the second pile. If you think it is very hard to get (or to use or to do), put it in the third pile. [Write response in the second table. Then shuffle.]

ଦୟାକରି ଏଥର ଚିନ୍ତା କରନ୍ତୁ କାର୍ଡରେ ଦେଖା ଯାଇଥିବା ପ୍ରତ୍ୟେକ ଜିନିଷକୁ କେତେ ସହଜରେ ପାଇ ପାରିବେ । ଯଦି ଭାବୁଛନ୍ତି ଏହି ଜିନିଷଗୁଡ଼ିକ ବହୁତ ସହଜରେ ପାଇପାରିବେ (ବା ବ୍ୟବହାରରେ ଲଗାଇପାରିବେ ବା କାମରେ ଲଗାଇପାରିବେ) ସେଗୁଡିକ ପ୍ରଥମ ଗଦାରେ ରଖନ୍ତୁ | ଯଦି ଭାବୁଛନ୍ତି ଏହି ଜିନିଷଗୁଡ଼ିକ ପାଇବାକୁ ବେଳେବେଳେ ସହଜ ଏବଂ ବେଳେବେଳେ କଷ୍ଟ (ବା ବ୍ୟବହାରରେ ଲଗାଇପାରିବେ ବା କାମରେ ଲଗାଇପାରିବେ), ତାହାକୁ ତୃତୀୟ ଗଦାରେ ରଖନ୍ତୁ | ଯଦି ଭାବୁଛନ୍ତି, ଏହା ପାଇବାକୁ (ବା ବ୍ୟବହାରରେ ଲଗାଇପାରିବେ ବା କାମରେ ଲଗାଇପାରିବେ) ବହୁତ କଷ୍ଟସାଧ ତାହାକୁ ତୃତୀୟ ଗଦାରେ ରଖନ୍ତୁ | (ଉତ୍ତରଗୁଡିକ ଦ୍ୱିତୀୟ ଟେବୁଲ ରେ ଲେଖନ୍ତୁ ଏବଂ ସେଗୁଡିକ ପରେ ମିଶାଇଦିଅନ୍ତୁ |)

Like/ପସନ୍ଦ

Finally, we will now sort these into three piles according to how much you like each card. If you like it a lot, put it in the first pile. If you like it just a bit or dislike it just a bit, put it in the second pile. And, if you dislike it a lot, put it in the third pile. [Record in the third table] ଶେଷରେ, ଆପଣ ପ୍ରତ୍ୟକ କାର୍ଡକୁ କେତେ ପସନ୍ଦ କଲେ, ସେହି ଅନୁସାରେ ଆଗେ ଏହାକୁ ତିନୋଟି ଗଦାରେ ସଜାଇ ରଖିବା | ଯଦି ଆପଣ ଏହାକୁ ବହୁତ ପସନ୍ଦ କରନ୍ତି, ତାହେଲେ

For peer review only - http://bmjopen.bmj.com/site/about/guidelines.xhtml

ତାକୁ ପ୍ରଥମ ଗଦାରେ ରଖନ୍ତୁ | ଯଦି ଆପଣ ଏହାକୁ କମ ପସନ୍ଦ କରନ୍ତି ବା କମ ପସନ୍ଦ କରନ୍ତି ନାହିଁ, ତାହାକୁ ଦ୍ୱିତୀୟ ଗଦାରେ ରଖନ୍ତୁ | ଯଦି ଆପଣ ସମ୍ପୃକ୍ତ ପସନ୍ଦ କରନ୍ତି ନାହିଁ, ତାହାକୁ ତୃତୀୟ ଗଦାରେ ରଖନ୍ତୁ) (ତୃତୀୟ ଟେବୁଲରେ ଲେଖନ୍ତୁ)

Write the card number in each column.

PILE 1: Very important ବହୁତ ଜରୁରୀ	PILE2: Somewhat important କିଛିମାତ୍ରାରେ ଜରୁରୀ	PILE 3: Unimportant ଜରୁରୀ ନୁହେଁ

PILE 1: Very easy to get ବହୁତ ସହଜରେ ପାଇ ହେବ	PILE 2: Somewhat easy କମ୍ ସହଜରେ ପାଇ ହେବ	PILE 3: Very hard to get ପାଇବାକୁ ବହୁତ କଷ୍ଟସାଧ୍ୟ

PILE 1: Like a lot ବହୁତ ପସନ୍ଦ କରନ୍ତି	PILE 2: Like somewhat କିଛି ପସନ୍ଦ କରନ୍ତି	PILE 3: Dislike ପସନ୍ଦ କରନ୍ତି ନାହିଁ

For peer review only

For peer review only - http://bmjopen.bmj.com/site/about/guidelines.xhtml

Supplement 2 – Focus Group Guide

Before Beginning the Interview:

- Welcome participant and introduce yourself

Hello, and thank you for speaking with me today. My name is _______ and I am a field researcher at DCOR. I am conducting a research study to understand the attitudes and beliefs of people in this community. Thank you so much for agreeing to participate and taking time out of your day.

Do you have any questions about the research and your participation before we start?

Warm-up Questions
I'm new to this community, [name District], can you tell me a little bit about it? For example, what kind of food do people eat here? And what do people do to earn money?

Women's Role in the Community

What are some of the typical things that women in this community do throughout the day? (probe: things like cooking, cleaning, taking care of kids, working outside the home, spending time with her husband).

Do women in this community have enough energy or time to these things each day?
- Look after her kids?
- Work outside the home?
- Cook?
- Spend time with her husband?
- Have more intimate time with her husband?

How are men and women treated differently in this community?
For women's life in general, are things getting better or worse?
- in her role as a mother?
- in her role as a wife?
- in her role as a daughter in law?

What are some of the concerns that women in this community have? (probe: concerns about health, money, their family, themselves, their future)

- Some people think that (woman's name) should first take care of her husband and kids before she worries about her own health. Other people think that she should first take care of her own health.

  How do you think most women in this community feel about that? What about most men?

  In some families in Odisha, women eat after their husband, children and mother in law eat. In others, women eat at the same time. What do you think about that? Is this changing?

For peer review only - http://bmjopen.bmj.com/site/about/guidelines.xhtml

Anemia and Iron Folic Acid (IFA) Knowledge

(Show an IFA tablet and liquid IFA)
Can someone tell me what this is? (probe: What does it do?)
Have you ever heard of anemia?
Many people in this community may not use the word “anemia” but they may have other words or phrases to talk about anemia. Can you tell me what some of those phrases are?
Tell me a little bit about what happens when someone has anemia (use the word or phrase identified above instead of “anemia”). Tell me how this person feels or how this person acts when they have anemia.
What do you think causes anemia? (probe: Not eating enough iron rich foods?)
What do you think makes anemia go away? (Probe: Can IFA tablets help? How about changing your diet? What kind of foods might help it go away?)

Anemia Related Behavior

Ok, now we’re going to make up a character. She is a female aged 23 years old. What should we call her? (Ask for suggestions and decide on a name together). She just got married and is 3 months pregnant with her first child. Her doctor just told her she has anemia.

The doctor told her to take iron tablets.

- If (same woman’s name) wanted to get IFA tablets, where could she get them? How difficult is it to *get* IFA tablets?
  - Can you tell me what she likes about taking the tablets and what she doesn’t like? (PROBE by asking more likes and dislikes)
  - Apparently, some women stop taking those tablets. Can you tell me why they stop?
  - Can you tell me what good things may happen if someone takes the tablets?
  - What do you think would help her take the tablets? Do you think (woman’s name)’s husband will support her to take the tablets? Not support her to take them? Or not say anything? (Ask for examples)
  - What about (woman’s name)’s mother in law? Will she support her in taking the tablets? Why or why not?

IFA Norms

- Is Anemia is a problem in the community? If yes How much of a problem?
- In general, who is typically expected to take IFA tablets? (Probe: pregnant women, adolescent girls, non-pregnant women?)
- Please think about most women like (name) who live in this community. How many of them take IFA tablets? Some? Few? Most?
- Is there anyone in (woman’s name) family (or her husband’s family) that she can talk to about taking IFA tablets? How much do you think she’ll listen to what they have to say about IFA tablets?

Information Sources

For peer review only - http://bmjopen.bmj.com/site/about/guidelines.xhtml

Imagine another woman who is not pregnant but interested in learning more about her health. What should we call her? (Ask for suggestions and decide on a name together).

Where does she go for information about health related matters?

Where can she get information about pregnancy, anemia, and IFA tablets from?

- How easy is it for her to get information about pregnancy, anemia, and/or IFA tablets? What difficulties would come up when trying to get this information?
- When a woman gets IFA tablet, what kind of information does she get about them?
- Probe: Dose, when to take or why to take it?
- For those of you that participate in self-help groups, can you tell me what kind of things you discuss?

Closing

That is the end of the questions I have for you, but do you have anything else you'd like to add to the discussion? Any little stories about anemia or IFA use in this community?

As a reminder, please do not share anything we spoke about today with anyone outside of this group.

Any questions?

Thank you for your time.

For peer review only

For peer review only - http://bmjopen.bmj.com/site/about/guidelines.xhtml

Is fatigue a cue to take iron supplements in Odisha India? A mixed methods investigation

SRQR Topic number	SRQR Topic	Page numbers (and line numbers if relevant)
	Title and Abstract	
S1.	Title	p. 1; line 1
S2.	Abstract	p. 2
	Introduction	
S3.	Problem formulation	p. 5 - 8
S4.	Purpose or research question	p. 9; lines 1-2 & 5-6
	Methods	
S5.	Qualitative approach and research paradigm	p. 9 - 14
S6.	Research characteristics and reflexivity	p. 9 - 10
S7.	Context	p. 9 - 10
S8.	Sampling strategy	p. 10 – 11
S9.	Ethical issues pertaining to human subjects	p. 11; lines 13 - 23
S10.	Data collection methods	p. 12 - 13
S11.	Data collection instruments and technologies	bottom of page 12 to top of page 13
S12.	Units of study	p.14
S13.	Data processing	p.14
S14.	Data analysis	p.14
S15.	Techniques to enhance trustworthiness	p.9-10
	Results/Findings	
S16.	Synthesis and interpretation	p. 15 - 18
S17.	Links to empirical data	p. 15 - 18
	Discussion	
S18.	Integration with prior work, implications, transferability, and contributions to the field	p. 21 - 22
S19.	Limitations	p. 22
	Other	
S20.	Conflicts of interest	p. 24
S21.	funding	p. 24

For peer review only

Is fatigue a cue to obtain iron supplements in Odisha India? A mixed methods investigation

Journal:	*BMJ Open*
Manuscript ID	bmjopen-2020-037471.R3
Article Type:	Original research
Date Submitted by the Author:	23-Sep-2020

Complete List of Authors:	Yilma, Hagere; The George Washington University Milken Institute of Public Health, Prevention and Community Health<br>Sedlander, Erica; George Washington University Milken Institute of Public Health, Prevention and Community Health<br>Rimal, Rajiv; Johns Hopkins Bloomberg School of Public Health Center for Teaching and Learning, Health, Behavior and Society<br>Pattnaik, Soumik; D-COR Consulting

**Primary Subject Heading**:	Global health
Secondary Subject Heading:	Public health, Global health
Keywords:	PUBLIC HEALTH, QUALITATIVE RESEARCH, Nutrition < TROPICAL MEDICINE, Anaemia < HAEMATOLOGY

SCHOLARONE™ Manuscripts

I, the Submitting Author has the right to grant and does grant on behalf of all authors of the Work (as defined in the below author licence), an exclusive licence and/or a non-exclusive licence for contributions from authors who are: i) UK Crown employees; ii) where BMJ has agreed a CC-BY licence shall apply, and/or iii) in accordance with the terms applicable for US Federal Government officers or employees acting as part of their official duties; on a worldwide, perpetual, irrevocable, royalty-free basis to BMJ Publishing Group Ltd ("BMJ") its licensees and where the relevant Journal is co-owned by BMJ to the co-owners of the Journal, to publish the Work in this journal and any other BMJ products and to exploit all rights, as set out in our licence.

The Submitting Author accepts and understands that any supply made under these terms is made by BMJ to the Submitting Author unless you are acting as an employee on behalf of your employer or a postgraduate student of an affiliated institution which is paying any applicable article publishing charge ("APC") for Open Access articles. Where the Submitting Author wishes to make the Work available on an Open Access basis (and intends to pay the relevant APC), the terms of reuse of such Open Access shall be governed by a Creative Commons licence – details of these licences and which Creative Commons licence will apply to this Work are set out in our licence referred to above.

Other than as permitted in any relevant BMJ Author's Self Archiving Policies, I confirm this Work has not been accepted for publication elsewhere, is not being considered for publication elsewhere and does not duplicate material already published. I confirm all authors consent to publication of this Work and authorise the granting of this licence.

For peer review only

Title: Is fatigue a cue to obtain iron supplements in Odisha India? A mixed methods investigation

Authors: Hagere Yilma, MPH[1], Erica Sedlander, DrPH, MPH[1], Rajiv N. Rimal, PhD[2], Soumik Pattnaik, MS[3]

[1] George Washington University, Milken Institute School of Public Health, Department of Prevention and Community Health
[2] Johns Hopkins University Bloomberg School of Public Health, Health, Behavior, and Society
[3] DCOR Consulting

Hagere Yilma, MPH (corresponding author)
950 New Hampshire, Department of Prevention and Community Health
202.994.1486
https://orcid.org/0000-0002-4042-3484
hyilma@gwu.edu

Erica Sedlander, DrPH, MPH
950 New Hampshire, Department of Prevention and Community Health
202.994.0849
esedlander@gwu.edu

Rajiv N. Rimal, PhD
624 N. Broadway, Department of Health, Behavior, and Society
rimal@jhu.edu

Soumik Pattnaik, MS
H.B Colony, Punjabi Colony, Satya Nagar, Bhubaneswar, Odisha, India
+91 94384 63984
soumik.dcor@gmail.com

For peer review only

Abstract

Objectives: This study aims to understand if fatigue, the main symptom of anemia, is a health concern that cues women and their referent groups to obtain iron folic acid supplements.

Design: This is a mixed methods study that consisted of a cross-sectional perceptual mapping and card sorting activity along with 16 focus group discussions with women of reproductive age, mothers-in-law, and men. Participants of the perceptual mapping and card sorting activity were asked to compare images of anemia-related items and concepts. Participants in the focus group discussions were asked about their daily life, aspirations and concerns among women, and perceptions of anemia and iron supplementation in the community. The quantitative data were analyzed through multidimensional scaling and ANOVAs in SPSS. The qualitative data were analyzed through applied thematic analysis using NVIVO.

Setting: Bhubaneswar, Odisha, India

Participants: Women of reproductive age (n =30), mothers-in-law (n = 30), and married men (n = 30) were randomly selected to participate in the perceptual mapping and card sorting exercise. A separate sample of each group was randomly selected for the focus group discussions (n = 148).

Primary and secondary outcome measures: We collected perceptions of dissimilarity between anemia-related items, including fatigue and medical items; the extent to which these items were perceived as important to health or likable; and qualitative information about gender norms.

Results: Cognitive maps and card sorting revealed that fatigue was conceptualized distinctly from items related to medical treatment and that perceptions around fatigue's

importance to health were low. Women from the focus groups reported that fatigue is a regular part of their daily life.

Conclusion: Our results indicate that fatigue is currently not an adequate cue to seek treatment, perhaps due to the normalization of fatigue as a part of women's daily life.

Keywords: anemia; gender norms; iron folic acid; perceptual mapping; women's health

For peer review only

Strengths and Limitations of the study

- We adapted a marketing methodology known as perceptual mapping, coupled with a card sorting exercise and focus group discussions to understand how women of reproductive age, and their relevant referent groups (i.e., mothers-in-law and men) perceive fatigue.
- Qualitative focus groups were used to minimize the ambiguity of the perceptual mapping plots.
- The size of each subsample was small (n=30 each), reducing our ability to find significant differences from the card sorting activity.

For peer review only

Background

Anemia is a condition characterized by a low red blood cell count that affects a quarter of all people worldwide.[1] If left untreated, anemia can lead to several negative health outcomes, including poor mental health,[2-3] reduced work capacity,[4] pregnancy complications,[5-6] and sometimes death.[3] Women of reproductive age are the most vulnerable to anemia,[1] as blood loss during menstruation and childbirth raises their risk.[7-10] Recent research suggests that inequitable social structures may exacerbate anemia prevalence in women, particularly as it relates to gender norms.[11-14, 1] Gender norms are a subset of social norms, which refer to expectations around how people of a specific gender are expected to behave.[15]

The World Health Organization (WHO) has made anemia one of six global nutritional targets.[16] Iron and folic acid supplementation is an effective strategy for the prevention and treatment of anemia in low- and middle- income countries.[17-19] Dosage recommendations vary by age, gender, and pregnancy status, but WHO recommends regular iron supplementation for non-pregnant women of reproductive age in countries where prevalence exceeds 40%.[17]

In India, where over half of women of reproductive age are anemic, [20] the promotion of iron supplements to children and women of reproductive age has been a part of the nutritional agenda since 1970.[21-22] However the expansion of promotion to non-pregnant, out-of-school women only began in 2013 through the National Iron Plus Initiative.[23] Despite India's recent life-course approach to promote iron supplements to all women, consumption remains low and anemia prevalence is still high.[20, 24] This may be due to gaps in current iron supplement distribution practices in the Indian health

For peer review only - http://bmjopen.bmj.com/site/about/guidelines.xhtml

system. Distribution outside of clinical settings target pregnant women or in-school adolescents; frontline workers provide tablets to pregnant women, and adolescent girls can get iron supplements weekly in school. Out-of-school, non-pregnant women do not have this access and must first go to a health facility to get tested before receiving treatment, which is typically iron supplementation for mild and moderate forms of anemia.[25, 26] This process requires an ability to detect anemia symptoms within oneself and seek medical care.

However, the primary manifestation of anemia, fatigue, may be ignored as a sign of illness.[27-28] In a review of the evolving conceptualizations of fatigue across time, Loriol (2017) notes that, historically, fatigue is considered a necessary outcome for physical energy expenditure and, consequently, commonly attributed to a hard day's work. The result is a prevailing social expectation that the more the one expends physical energy, the more fatigued one is. This is seen in the inverse association between fatigue and socioeconomic status. [29] Therefore, women who exert a lot of physical energy in a day may not feel their fatigue is cause for alarm. Rather, they may feel that the fatigue they experience is a normal outcome of their hard work.

It is no surprise then that more than 80% of women in Odisha, India do not recognize tiredness or weakness as symptom of anemia. [26] Studies in similar cultures have also shown that women may not readily associate fatigue with illness.[30] In fact, anemic women who reported fatigue during pregnancy did not seek medical attention because they believed their anemia-related fatigue was just a normal part of pregnancy.[31-32]

For peer review only - http://bmjopen.bmj.com/site/about/guidelines.xhtml

FATIGUE AS A CUE TO SEEK TREATMENT

While the anemia literature among non-pregnant women in India is sparse, research in other parts of the world has shown that fatigue represents a conceptually different dimension of symptoms than somatic symptoms, even when both forms share an underlying cause.[33-34] Further, Ball et al. (2017) demonstrate that women's perceptions of fatigue symptoms are influenced by familial factors, whereas men hold perceptions of fatigue that are influenced by genetic factors.[33] A study in an area with high anemia prevalence showed that culture and gender norms can shape perceptions of anemia, which can in turn influence treatment-seeking behaviors among non-pregnant women.[13] Therefore, a similar normalization of fatigue may exist among non-pregnant women in rural India, as expectations for labor may lead to exhaustion that is difficult to disentangle from anemia-related fatigue. In fact, fatigue is commonly associated with activities like caretaking and employment in women but not men. [35]

In this paper, we explore the extent to which women perceive fatigue as a cue to seek medical care. While the literature around the conceptualization of fatigue as a symptom of illness is limited, recent research in India has shown that inequitable gender norms may indirectly affect a woman's ability or desire to access iron supplements.[11-14, 36] Other studies also show that women in Odisha tend to have less power within the household than their husbands, and carry the majority of the domestic workload burden.[37] As daily life is characterized by large amounts of physical labor (for both men and women), fatigue may be considered a normal part of everyday life, and thus, not a cue to obtain iron supplements. Clearly, knowledge about the prevalence and risks of anemia is critical as risk perception may be connected to gender norms. If women believe

For peer review only - http://bmjopen.bmj.com/site/about/guidelines.xhtml

that fatigue is just a necessary outcome of their role as women, rather than a symptom of illness, increasing risk perception may be an important first step.

Additionally, studies in India have highlighted the influence of shared-decision making within households as a key factor in anemia prevalence among women.[38-39] To our knowledge, how this relates to different conceptualizations of fatigue by various household members – husbands, mothers-in-law, and others – has not yet been explored. Yet, this is an important consideration: If women themselves do not equate fatigue with a need to seek medical care, do influential others in her home view fatigue in the same manner? Hence, our research questions are:

RQ1. Are perceptions of fatigue a cue to obtain iron supplements among women of reproductive age in Odisha, India?

RQ2. What are the differences in perceptions of fatigue across women's referent groups (their mothers-in-law and husbands)?

Methods

We used multiple methods to understand how conceptualizations of fatigue may relate to treatment-seeking behaviors among women of reproductive age in Odisha, India. We report findings from two methods, one quantitative and the other qualitative, used in this paper. First, we used perceptual mapping techniques and a card sorting activity to understand how our participants conceptualized fatigue.

Perceptual mapping is often used in commercial marketing [40-42] but it has recently gained momentum in health behavior communication. [43-47] In the current

For peer review only - http://bmjopen.bmj.com/site/about/guidelines.xhtml

study, we expand on the public health application of perceptual mapping by using this technique to understand how women understand fatigue in relation to items associated with the acquisition of iron tablets. We follow the perceptual mapping activity with a card sorting exercise to understand if cognitive (i.e. perceived importance to health) and affective (i.e. dislike) perceptions could inform the results of the perceptual maps.

While perceptual maps can highlight conceptual associations (or disassociations) between concepts, it is difficult to pinpoint the dimensions of perception around which these similarities or differences exist. We included a card sorting activity to probe the cognitive and affective dimensions of perception. However, a qualitative component is also needed to delineate why these associations or disassociations may exist. Therefore, we followed the quantitative methods with qualitative focus group discussions to triangulate our findings and identify the social and cultural underpinnings of how fatigue is conceptualized.

All components of this study come from formative research conducted for the Reduction in Anemia through Normative Innovations (RANI) Project. The RANI Project is implementing and evaluating a social norms-based anemia reduction intervention. The overall goals of the formative research components were larger than the research questions presented here; all of the goals of the formative research beyond this study are outlined the RANI project formative research protocol. [48]

Sampling

The perceptual mapping and card sorting activity collected data from a different sample than the focus group discussion to accommodate the larger formative research goals of the methods and limit participant burden. However, all samples came from the

For peer review only - http://bmjopen.bmj.com/site/about/guidelines.xhtml

Angul District of Odisha, India and shared the same random sampling procedure. To do so, researchers first tabulated all the homes in each of the four villages where we worked and obtained the total number of eligible participants. Based on the number of participants needed for the focus groups, against a sampling frame that consisted of the entire village, we used a proportional skip pattern that began with a randomly selected initial participant to identify households from which to select every succeeding participant for each sub-group. From the remaining households, a similar selection process was applied to select the participants for the perceptual mapping and card sorting activity.

We conducted 16 focus groups with women of reproductive age, mothers-in-law, adolescent girls, and husbands (n = 124). The sample description is published elsewhere.[11] The final sample for the perceptual mapping and card sorting exercise comprised women of reproductive age (n=30), mothers-in-law (n=30), and men (n=30). All participants were required to live in the Angul District, speak Odiya, and give written consent before participating. Parental permission was obtained for all participants younger than 18 years old. A small gift of a bowl and spoon was given as incentive for participating.

Patient and Public Involvement

This study did not involve patients or the public in its development or implementation. Upon completion of the study, results were shared with various stakeholders at a two-day convening in Bhubaneswar, Odisha, India.

Procedure

For peer review only - http://bmjopen.bmj.com/site/about/guidelines.xhtml

The George Washington University Institutional Review Board (IRB) and a local ethics board, D-COR Consulting Institutional Ethics Committee (IEC) reviewed and approved this study before recruitment began.

Perceptual Mapping. During the perceptual mapping exercise, we showed participants two cards at a time, each with a picture of a different anemia-related item, and we asked them to compare how similar the two were to each other. We recorded their rating on a scale from 1 to 4, where 4 indicated the most similarity. We had 12 cards in all, and all 66 permutations (1 versus 2, 1 versus 3, … 11 versus 12) were shown to each participant. A more detailed explanation of the perceptual mapping procedure can be found in the Formative Research Protocol. [48]

Card Sorting. We also asked participants to do a card sorting exercise upon completing the perceptual mapping exercise. In this portion of the study, we asked participants to place the same cards into three piles based on the items level of importance for their health and well-being. Upon completing this task, participants were then asked to sort the cards into another set of three piles based on the likability of the item. Data collectors recorded the compositions of each pile.

Focus Group Discussions. Trained researchers (native speakers from Odisha India) conducted all focus groups, and we matched interviewer and interviewee on gender. For each focus group, one member of the research staff observed the group while taking notes and researchers wrote field notes after each interview. Audio recordings were transcribed in Odiya and subsequently translated into English for data analysis. A more detailed description of the focus group discussion procedures can be found in a separate study. [11]

For peer review only - http://bmjopen.bmj.com/site/about/guidelines.xhtml

Instruments

All instruments were developed based on discussions with local researchers and a review of the literature to ensure cultural and theoretical relevance.[25, 49-50] For the perceptual mapping and card sorting activity, we used large cards with the images of interest printed on them. The 12 cards included an image of one of the following: iron tablets, a clinic, pharmaceutical kiosks, women empowerment groups known as self-help groups, traditional healer, physician, fatigue, prenatal care, green vegetable, meat/fish, pregnancy, or stomach ache. Labels of each item were also included on each card. Our colleagues in Odisha, India assessed the cards for face validity. Interview guides for the focus group discussions covered general questions about what women do on a typical day, their concerns and aspirations, and roles in the family and community. The questionnaire for the perceptual mapping and card sorting activity (Supplement 1), along with the focus group discussion guides (Supplement 2) are included as supplementary material.

Analysis

Perceptual Maps. Participant ratings were recoded to indicate dissimilarity and then organized into matrixes. We used multidimensional scaling (MDS) to graphically plot the averaged dissimilarities of the overall sample as well as each subgroup recorded (i.e., women of reproductive age, mothers-in-law, and men; Figures 1-3). MDS is a common method to analyze perceptual mapping data. [51] Theoretically, the resulting maps can display items on multiple axes to represent underlying dimensions of perception. For simplicity and ease of interpretation, they are often distilled down to two (*x* and *y*) dimensions. The maps display dissimilarity so that the further two points are

For peer review only - http://bmjopen.bmj.com/site/about/guidelines.xhtml

from each other, the more the participants perceived dissimilarity between the two items. Conversely, the closer the two points are on the map, the more the participants perceived similarity.

Card Sorting. We converted the responses from the card sorting exercise into ranks from 0-2, where a score of 0 was given to cards that were placed in the "not very important to health" pile or the "dislike" pile, and a score of 2 was given to the cards that were in the "very important to health" pile or "like a lot" pile, for their respective parameters. Scores for liking were reverse coded so that higher values indicated a dislike. We analyzed the card sorting data using an ANOVA across the three participant groups. We conducted post-hoc analyses to determine homogenous subsets when omnibus differences were detected. There we no missing responses. All analysis were done in SPSS v.25.

Focus Group Discussions. We uploaded transcripts and the codebook into NVivo v.12 for analysis (Nvivo, 2015). We developed a draft codebook then applied thematic analysis to characterize and subsequently modify emergent themes through an iterative approach that combined data collection and analysis concurrently. [52] This allowed us to determine when saturation occurred, when no new themes emerged from the data.

Research Team and Reflexivity

Of the four authors, one is from Angul, India, one is from Nepal, and two are from the United States. Their backgrounds vary from overseeing data collection efforts to social norms/gender norms, and global behavioral health. Each researcher holds a master's and/or doctoral degree and their familiarity with anemia in India range from

For peer review only - http://bmjopen.bmj.com/site/about/guidelines.xhtml

academic research to personal experience. Three of the authors, SP, ES, and RR, were present for the data collection training and piloting. One of the authors, SP, was present for all data collection. None of the authors have any personal relationship to the participants or any direct benefit from examining the relationship between anemia and fatigue.

For peer review only

Results

Sample

The demographic distribution of each subgroup can be found in Table 1. The average age of men in the sample was 35 years (*SD* = 6.00), and that of women was 28 years (*SD* = 4.76). The average age of mothers-in-law was 56 years (*SD* = 7.58). The majority of women of reproductive age had completed at least secondary school, while the majority of men had not completed secondary school. The majority of mothers-in-law had not received any education. Additionally, the majority of participants had at least two children. All but one participant was also married; the singular participant who was unmarried was widowed. All participants were Hindu.

While the majority of women of reproductive age had taken iron folic acid in the past (86.7%), very few were taking it at the time of the survey (10%). Only about a quarter of mothers-in-law had ever taken iron folic acid, but 9.1% were taking it at the time of the survey. None of the men surveyed reported currently taking iron folic acid, and only 13.3% had ever taken it. About half of women of reproductive age had ever received an anemia diagnosis, and 14.3% reported currently being anemic at the time of

For peer review only - http://bmjopen.bmj.com/site/about/guidelines.xhtml

the survey. The demographic distribution of participants who took part in the focus group discussions was similar to the participants who took part in the perceptual mapping activity. [11]

Perceptions of Fatigue

Perceptual maps. The perceptual maps for women of reproductive age show a conceptual separation, as evidenced by their spatial distance, between fatigue and the practical iron folic acid distribution channels for non-pregnant women; one quadrant of the map contains fatigue, while a separate one contains the items related to the iron supplement distribution channels for non-pregnant women, including physicians, clinics, and pharmaceutical kiosks (Figure 1). These three items are clustered together in one quadrant, indicating that they are perceived as similar to each other. Iron folic acid is also plotted in the same quadrant as this cluster, indicating that women of reproductive age may conceptualize physicians, clinics, and pharmaceutical kiosks as links to iron supplements. The separation of fatigue from these iron supplement distribution channels indicates that fatigue may not act as a cue to obtain iron supplements.

Card Sorting Exercise. Confirming these findings, the card sorting exercise showed that women of reproductive age were less likely to report fatigue as important to health compared to items related to the iron supplement distribution channels for non-pregnant women (Table 2). Fatigue was considered to be only somewhat important to health by women (M = 0.77, SD = 0.73), while the iron supplement channels, including physicians (M = 1.80, SD = 0.55), clinics (M = 1.80, SD = 0.48), and pharmaceutical kiosks (M = 1.77, SD = 0.50), were considered very important to health.

For peer review only - http://bmjopen.bmj.com/site/about/guidelines.xhtml

Differences in the conceptualization of fatigue and the items related to iron supplement distribution channels for non-pregnant women may also be explained by differences in affective ratings (Table 2). Fatigue was reported to be disliked by women ($M = 1.47$, $SD = 0.68$), while the other iron folic acid channel-related items were hardly disliked at all (physician: $M = 0.20$, $SD = 0.41$; clinics: $M = 0.23$, $SD = 0.50$; pharmaceutical kiosks: $M = 0.27$, $SD = 0.58$).

Focus Group Discussions. Women reported that fatigue, and oftentimes, extreme fatigue, was a normal part of daily life. They accepted fatigue as part of their role as wives and mothers; they take care of all of the household responsibilities including all childcare responsibilities, serve their husband and in-laws, and often work outside of the home to earn extra money for the family. One woman said, *"We have to work all the time, go to the mountains and fields, have children to take care of. We get no time to rest. We work in unhappiness and eat in pain"* (woman of reproductive age). Women did not think of fatigue as something that they could change and they did not associate their own fatigue with the possibility of having anemia. Although most women knew that fatigue is a symptom of anemia, they underestimated the prevalence of anemia in their village despite endemic rates. A frontline health worker said, *"In our village, in a year, 2-3-4 women have it [anemia]...mainly pregnant women"* (frontline health worker). This is, of course, a far cry from what the actual numbers would be, based on data from the National Family Health Survey, [20] which shows that over half of women of reproductive age are anemic.

Perceptions of Fatigue across Referent Groups

For peer review only - http://bmjopen.bmj.com/site/about/guidelines.xhtml

Perceptual maps. Men (Figure 2) and mothers-in-law (Figure 3) also perceived fatigue as different from the other items related to the iron supplement distribution channels. The perceptual map generated according to men's responses displays physicians, pregnancy, and prenatal care as plotted closely together in the same quadrant. This cluster also contains iron supplements, indicating that men understand these items as existing in the same cognitive space. Similarly, the perceptual map generated for mothers-in-law displays physicians, clinics, and pharmaceutical kiosks as a cluster plotted near the plot-point for iron supplements. Fatigue, however, is plotted in a separate quadrant from these items related to the iron supplement distribution channels for non-pregnant women. The separation of fatigue from these channels indicates that fatigue may not act as a cue to obtain iron supplements among husbands and mothers-in-law.

Card Sorting Exercise. We also explored differences in perception across participant groups through the card sorting analysis. Perceptions of importance to health didn't differ across participant groups for any of the anemia-related items shown, with the exception of fatigue ($F = 9.66$, $p < 0.001$) and stomachache ($F = 5.76$, $p < 0.01$), which were significantly different between women and their referent groups. Affective perceptions (i.e. dislike) related to fatigue were also statistically different across the three groups as well ($F = 8.29$, $p < 0.001$).

Post-hoc Least Significant Differences (LSD) tests revealed that women and men had similar perceptions of fatigue across these cognitive and affective dimensions (i.e., importance to health and dislike), but mothers-in-law had significantly different perceptions. Fatigue was perceived with the same level of importance for health by both men ($M = 0.80$, $SD = 0.61$) and women ($M = 0.80$, $SD = 0.61$). However, mothers-in-law

For peer review only - http://bmjopen.bmj.com/site/about/guidelines.xhtml

had significantly lower perceptions about the importance of fatigue ($M = 0.17$, $SD = 0.53$) than men ($p < 0.001$) and women ($p < 0.001$). Additionally, men reported a similar dislike of fatigue as women ($M = 1.23$, $SD = 0.73$, n.s.) while mothers-in-law disliked fatigue to a much greater extent ($M = 1.87$, $SD = 0.35$, $p < 0.05$).

Focus Group Discussions. Men acknowledged that women bear the brunt of the work at home and often face extreme fatigue but did not make the connection that anemia could be at play. A husband from a focus group said, *“They [women] don’t have time to do all these works. To do the outside works after completing the household works, they don’t get enough time”* (husband). Mothers-in-law felt that their daughters-in-law had it “easier” today than when they were young and showed less empathy than men for their daughters-in-law heavy workload. They also mentioned that younger women today seek medical care more often than their generation. A mother-in-law said, “*We never had those tablets [iron supplements]. We had no idea that we are pregnant until the 5th month but women of today know everything from the 1st month and run to the hospital*” (mother-in-law).

Discussion

This study aimed to understand if the conceptualization of fatigue allowed it to act as a cue for illness in women as well as referent others in their network (i.e., mothers-in-law and husbands). We found that fatigue may not act as a sufficient cue for women to seek treatment for anemia. Nor is fatigue conceptualized in a similar manner across the referent groups. These findings were notable in the perceptual maps and card sorting activity, and further manifest through the focus group discussions.

For peer review only - http://bmjopen.bmj.com/site/about/guidelines.xhtml

FATIGUE AS A CUE TO SEEK TREATMENT

The perceptual mapping activity showed a distinction between fatigue and the cluster of items related to the iron supplement distribution channels, which included physicians, clinics, and pharmaceutical kiosks. These clinical items represent necessary steps in the iron folic acid acquisition process for non-pregnant women; the disassociation between fatigue and these clinical items indicates the conceptual distinction between fatigue and a need for medical care. Additionally, the card sorting exercise showed cognitive and affective gaps in perceptions between fatigue, on the one hand, and the aforementioned clinical items, on the other. In particular, women did not perceive fatigue as an important consideration for their health, although they reported dislike for it. These findings are in line with previous literature that discusses the normalization of fatigue as an outcome of hard work rather than an illness. [29]

The qualitative results support the lack of association between fatigue and medical care in the perceptual mapping and card sorting activity. The focus group discussions clarify these findings through the identification of gender norms that may set the conditions for what mental and physical phenomenon are perceived as illness.[11-14, 36] Women in the focus group discussions highlight a prevailing gender norm that women are expected to maintain a large amount of the household duties through the exertion of physical labor, which in turn, can lead to the expectation of fatigue.[29] Women in the focus groups also indicated that they prioritize the wellbeing of their family over their own. Thus, they accept the fatigue that they feel as a normal outcome of their household role. These unequal gender norms that place the majority of household duties on the woman may normalize fatigue in women and negatively affect their risk

For peer review only - http://bmjopen.bmj.com/site/about/guidelines.xhtml

perception. Thus, fatigue is not an adequate cue that illness is present, and consequently, does not act as a cue to seek treatment.

This study also aimed to investigate conceptualizations of fatigue in mothers-in-law and men. The perceptual maps created for mothers-in-law showed a slightly larger dissociation between fatigue and the clinical items related to ion supplementation than was observed in women of reproductive age. While perceptual mapping methods do not allow for the determination of statistically significant differences, the results of the focus group discussion show that the older generation of women believe that younger women have more sensitive perceptions of health. The mothers-in-law in this study harbored attitudes that were even more dismissive of fatigue than men or younger women; they did not consider fatigue important to health. These results indicate that the normalization of symptoms, like fatigue, may be stronger among older generations of women.

We found a different conceptualization of fatigue in men. The card sorting activity revealed a smaller gap in cognitive and affective perceptions of fatigue; while men may consider fatigue a low health concern, they also do not mind it very much. The results of the focus group discussions offer the explanation that men may not mind fatigue as much as the older generations of women because they are subject to different expectations of labor. The men in the focus group discussions show empathy for women who complete the lion’s share of household work and often work outside the home. The results of the qualitative investigations coupled with the quantitative findings indicates that fatigue is normalized to a lesser extent in men than it is in women.

The conceptualization of fatigue as illness is critical for non-pregnant, out-of-school women who are currently not given iron supplements as part of regular care by

For peer review only - http://bmjopen.bmj.com/site/about/guidelines.xhtml

front line health workers. If women do not perceive illness when fatigue is present, they may not seek treatment for it (including asking for iron supplements) unless they are pregnant. This may exacerbate an existing gap in iron supplement distribution to non-pregnant, out-of-school women, who make up the largest proportion of women with anemia. [20] Future anemia reduction interventions should take into account the social and cultural underpinnings related to perceptions of illness among women.

Studies show that mothers-in-law are intimately involved in the decision-making processes among anemic women in India. [39, 53-54] If mothers-in-law continue to believe and perpetuate the idea that fatigue is not a symptom of illness, women of reproductive age may also follow suit. Even in situations where a woman does not agree with her mother-in-law, it may be difficult for her to obtain iron supplements against her mother in laws requests.[55] Therefore, in line with the recommendations of other studies, we highlight the importance of shifting mothers-in-law's perceptions of fatigue and anemia, so that perceptions can shift in women as well.[39]

To our knowledge, this is the first study that explores the gaps in iron folic acid distribution for non-pregnant women through an examination of the social context of women living in Odisha, India. The current available health services require that non-pregnant, out-of-school, women seek treatment for their anemia, rather than receive iron supplements unprompted. However, the most recognizable symptom of anemia, fatigue, is not a cue for illness, and thus, does not act as a cue to seek treatment. This study shows that fatigue is not an adequate cue to seek treatment among non-pregnant women resulting in a failure to treat their anemia. We recommend improving the available services to non-pregnant women so that they can obtain iron supplements through

For peer review only - http://bmjopen.bmj.com/site/about/guidelines.xhtml

frontline workers. However, within the current health practice landscape of Odisha, India, we recommend increasing knowledge around the connection between fatigue and anemia among mothers-in-laws, husbands, and women to improve iron supplementation use.

Limitations. Findings from this study provide important contributions to the anemia-reduction literature. However, there are several limitations in this study that must be acknowledged. First, the quantitative methods used in this analysis leave gaps in interpretation. Perceptual maps are a useful tool for depicting the relative conceptualization of items and concepts, but it gives little explanation for the dimension of perception that are depicted. We included a card sorting analysis to attempt to understand the cognitive and affective dimensions. However, there is no method to triangulate these two quantitative methods to understand if the maps are created on these two dimensions. For this reason, we have included a qualitative component, which we have triangulated with the results observed in the quantitative component. We have also used the qualitative component to provide context to the results of the quantitative assessment, despite the fact that they come from different samples. The use of different samples to triangulate may pose as a limitation, but it is worthwhile to note both components share a common sampling procedure and sampling frame.

Second, we note that there are limitations in the way that fatigue can be depicted through our quantitative methods. Participants of the perceptual mapping and card sorting activity were shown a picture of a woman experiencing fatigue, followed by a depiction of the word in the local language. It is possible that the term was too technical to be understood properly. However, the face validity of the cards were evaluated by co-investigators in the field minimize the chances of misinterpretation. Additionally, the

For peer review only - http://bmjopen.bmj.com/site/about/guidelines.xhtml

majority of men and women in Odisha are literate. [56] We also note that the broader goal of the qualitative component was to understand key barriers and facilitators of iron supplementation. As a result, participants were not explicitly asked about their conceptualizations of fatigue. Hence, it is possible that their responses may shift if asked about it directly. Despite this, respondents still revealed a normalization of hard labor among women that corresponds to normalizations of fatigue in the literature. [29] Additionally, it is likely that not all fatigue that women are reporting is related to anemia. Therefore, it may be useful to measure perceptions of changes in fatigue as a cue to seek medical attention, as women may have a baseline level of fatigue that is not related to anemia at all.

For peer review only

For peer review only - http://bmjopen.bmj.com/site/about/guidelines.xhtml

Funding: This study was funded by the Bill and Melinda Gates Foundation under Grant OPP1182519. The funders had no role in study design, data collection and analysis, decision to publish, or preparation of the manuscript.

Author Contributions: HY, ES, and RR made substantial contributions to the conception or design of the work, the analysis, and the interpretation of the work. SP also contributed to the design of the study, and led the data acquisition of data with help from ES and RR. All authors were involved in drafting the work or revising it critically and the final approval of the version to be published. All authors are accountable for all aspects of the work.

Declaration Statement: The authors of this paper have no conflicts of interest to report.

Ethics statement: This study was reviewed and approved by the George Washington University Institutional Review Board (IRB) as well as D-COR Consulting Institutional Ethics Committee (IEC). Data collectors obtained informed consent from all participants in the study.

Data Availability: The data associated with this study can be made available upon request. Anyone who wishes to have access to the de-identified data from the perceptual mapping activity, card sorting activity, or focus group discussions can email the study data manager, Hagere Yilma (corresponding author), at hyilma@gwu.edu. All data is de-identified. Investigators are permitted to access the data for any research questions that have yet to be explored by the study team.

For peer review only

References

1. Benoist, B. d., McLean, E., Egll, I., & Cogswell, M. (2008). Worldwide prevalence of anaemia 1993-2005: WHO global database on anaemia. *Worldwide Prevalence of Anaemia 1993-2005: WHO Global Database on Anaemia.*
2. Strauss, W. E., & Auerbach, M. (2018). Health-related quality of life in patients with iron deficiency anemia: Impact of treatment with intravenous iron. *Patient Related Outcome Measures, 9*, 285.
3. Wouters, H. J., van der Klauw, Melanie M, de Witte, T., Stauder, R., Swinkels, D. W., Wolffenbuttel, B. H., et al. (2019). Association of anemia with health-related quality of life and survival: A large population-based cohort study.*Haematologica, 104*(3), 468-476.
4. Gardner, G. W., Edgerton, V. R., Senewiratne, B., Barnard, R. J., & Ohira, Y. (1977). Physical work capacity and metabolic stress in subjects with iron deficiency anemia. *The American Journal of Clinical Nutrition, 30*(6), 910-917.
5. Allen, L. H. (2000). Anemia and iron deficiency: Effects on pregnancy outcome. *The American Journal of Clinical Nutrition, 71*(5), 1284S.
6. Brabin, B. J., Hakimi, M., & Pelletier, D. (2001). An analysis of anemia and pregnancy-related maternal mortality. *The Journal of Nutrition, 131*(2), 615S.
7. Beard, J. L. (2000). Iron requirements in adolescent females. *The Journal of Nutrition, 130*(2), 442S.
8. Cooke, A. G., McCavit, T. L., Buchanan, G. R., & Powers, J. M. (2017). Iron deficiency anemia in adolescents who present with heavy menstrual bleeding. *Journal of Pediatric and Adolescent Gynecology, 30*(2), 247-250.

For peer review only - http://bmjopen.bmj.com/site/about/guidelines.xhtml

9. Hallberg, L., Högdahl, A., Nilsson, L., & Rybo, G. (1966). Menstrual blood loss and iron deficiency. *Acta Medica Scandinavica, 180*(5), 639-650.

10. Milman, N. (2011). Postpartum anemia I: Definition, prevalence, causes, and consequences. *Annals of Hematology, 90*(11), 1247.

11. Sedlander, E., Long, M. W., Mohanty, S., Munjral, A., Bingenheimer, J. B., Yilma, H., & Rimal, R. N. (2020). Moving beyond individual barriers and identifying multi-level strategies to reduce anemia in Odisha India. *BMC Public Health, 20*, 1-16.

12. Aurino, E. (2017). Do boys eat better than girls in India? Longitudinal evidence on dietary diversity and food consumption disparities among children and adolescents. *Economics & Human Biology, 25*, 99-111.

13. Bedwell, R. M., Spielvogel, H., Bellido, D., & Vitzthum, V. J. (2017). Factors influencing the use of biomedical health care by rural bolivian anemic women: Structural barriers, reproductive status, gender roles, and concepts of anemia. PloS One, 12(1), e0170475.

14. Jose, S. (2011). Adult undernutrition in India: Is there a huge gender gap? *Economic and Political Weekly*, 95-102.

15. ALIGN. Advancing Learning and Innovation on Gender Norms. (2019). https://www.alignplatform.org/FAQ. Accessed May 26th, 2020.

16. World Health Organization. (2014). *Global nutrition targets 2025: Anaemia policy brief*. Geneva, Switzerland: World Health Organization.

17. Ahmed, F., Khan, M. R., Akhtaruzzaman, M., Karim, R., Williams, G., Torlesse, H., et al. (2010). Long-term intermittent multiple micronutrient supplementation enhances hemoglobin and micronutrient status more than iron folic acid

For peer review only - http://bmjopen.bmj.com/site/about/guidelines.xhtml

supplementation in bangladeshi rural adolescent girls with nutritional anemia. *The Journal of Nutrition, 140*(10), 1879-1886.

18. Vir, S. C., Singh, N., Nigam, A. K., & Jain, R. (2008). Weekly iron and folic acid supplementation with counseling reduces anemia in adolescent girls: A large-scale effectiveness study in uttar pradesh, india. *Food and Nutrition Bulletin, 29*(3), 186-194.

19. World Health Organization. (2016). *Guideline: Daily iron supplementation in adult women and adolescent girls*. Geneva, Switzerland: World Health Organization.

20. International Institute for Population Sciences. (2017). *National family health survey (NFHS-4), 2015-16*. India. Mumbai: IIPS.

21. Bellad, A., Kapil, R., & Gupta, A. (2018). National guidelines for prevention and control of iron deficiency anemia in india. *Indian Journal of Community Health, 30*(01), 89-94.

22. Kumar, A. (1999). National nutritional anaemia control programme in India. *Indian Journal of Public Health, 43*(1), 3-5.

23. Kapil, U., & Bhadoria, A. S. (2014). National iron-plus initiative guidelines for control of iron deficiency anaemia in India, 2013. *Natl Med J India, 27*(1), 27-29.

24. Nguyen, P. H., Scott, S., Avula, R., Tran, L. M., & Menon, P. (2018). Trends and drivers of change in the prevalence of anaemia among 1 million women and children in India, 2006 to 2016. *BMJ Global Health, 3*(5), e001010.

25. Rai, R. K., Fawzi, W. W., Barik, A., & Chowdhury, A. (2018). The burden of iron-deficiency anaemia among women in India: How have iron and folic acid interventions fared? *WHO South-East Asia Journal of Public Health, 7*(1), 18.

For peer review only - http://bmjopen.bmj.com/site/about/guidelines.xhtml

26. UNICEF, & AIIMS. (2018). *Process documentation of national iron plus initiative (NIPI) – towards an anaemia free Odisha, 2016-17*. Bhubaneswar, Odisha: UNICEF.

27. Bager, P. (2014). Fatigue and acute/chronic anaemia. *Dan Med J*, *61*(4), B4824.

28. Sobrero, A., Puglisi, F., Guglielmi, A., Belvedere, O., Aprile, G., Ramello, M., et al. (2001). Fatigue: A main component of anemia symptomatology. Paper presented at the *Seminars in Oncology, 28.* pp. 15-18.

29. Loriol, M. (2017). A sociological stance on fatigue and tiredness: Social inequalities, norms and representations. *Neurophysiologie Clinique/Clinical Neurophysiology, 47*(2), 87-94.

30. Maaita, M., Irtaimeh, T., Altaleb, N., & Ibrahim, R. (2018). Perception of iron deficiency anemia (IDA) and compliance of iron supplements among pregnant women admitted to the labor ward at King Hussein Medical Center, Amman, Jordan. *JRMS, 25*(2), 12-18.

31. Shankar, M., & Reddy, B. (2012). Anaemia in pregnancy still a major cause of morbidity and mortality: insights from Koppal district, Karnataka, India. *Reproductive health matters, 20*(40).

32. Vincent, A., Keerthana, K., Damotharan, K., Newtonraj, A., Bazroy, J., & Manikandan, M. (2017). Health care seeking behaviour of women during pregnancy in rural south India: A qualitative study. *International Journal of Community Medicine and Public Health, 4*(10), 3636-3639.

33. Ball, H. A., Siribaddana, S. H., Sumathipala, A., Kovas, Y., Glozier, N., Rijsdijk, F., ... & Hotopf, M. (2011). Genetic and environmental contributions to the overlap

For peer review only - http://bmjopen.bmj.com/site/about/guidelines.xhtml

between psychological, fatigue and somatic symptoms: a twin study in Sri Lanka. *Twin Research and Human Genetics, 14*(1), 53-63.

34. Ray, C., Weir, W. R. C., Cullen, S., & Phillips, S. (1992). Illness perception and symptom components in chronic fatigue syndrome. *Journal of Psychosomatic Research, 36*(3), 243-256.

35. Bensing, J. M., Hulsman, R. L., & Schreurs, K. M. (1999). Gender differences in fatigue: biopsychosocial factors relating to fatigue in men and women*. Medical care, 37(*10), 1078-1083.

36. Diamond-Smith, N. G., Gupta, M., Kaur, M., & Kumar, R. (2016). Determinants of persistent anemia in poor, urban pregnant women of Chandigarh city, North India: a mixed method approach. Food and nutrition bulletin, 37(2), 132-143.

37. Seymour, S. C. (2010). Environmental change, family adaptations, and child development: Longitudinal research in India. Journal of cross-cultural psychology, 41(4), 578-591.

38. Krupp, K., Placek, C. D., Wilcox, M., Ravi, K., Srinivas, V., Arun, A., & Madhivanan, P. (2018). Financial decision making power is associated with moderate to severe anemia: A prospective cohort study among pregnant women in rural South India. *Midwifery*, *61*, 15-21.

39. Varghese, R., & Roy, M. (2019). Coresidence with mother-in-law and maternal anemia in rural India. *Social Science & Medicine, 226*, 37-46.

40. Adaval, R., Coupey, E., & Narayanan, S. (2015). Direct mapping of consumer perceptions. Paper presented at the *Proceedings of the 1995 Academy of Marketing Science (AMS) Annual Conference,* pp. 255-263.

41. Hastings, G., Angus, K., & Bryant, C. (Eds.). (2011). *The Sage handbook of social marketing*. Sage.

42. Iacobucci, D., Grisaffe, D., & DeSarbo, W. (2017). Statistical perceptual maps: Using confidence region ellipses to enhance the interpretations of brand positions in multidimensional scaling. *Journal of Marketing Analytics, 5*(3-4), 81-98.

43. Bass, S. B., Jessop, A., Maurer, L., Gashat, M., Al Hajji, M., & Gutierrez, M. (2018). Mapping the barriers and facilitators of HCV treatment initiation in methadone maintenance therapy patients: Implications for intervention development. *Journal of Health Communication, 23*(1), 117-127.

44. Bass, S. B., Wolak, C., Greener, J., Tedaldi, E., Nanavati, A., Ruppert, K., et al. (2016). Using perceptual mapping methods to understand gender differences in perceived barriers and benefits of clinical research participation in urban minority HIV patients. *AIDS Care, 28*(4), 528-536.

45. Pivonka, E., Seymour, J., McKenna, J., Baxter, S. D., & Williams, S. (2011). Development of the behaviorally focused Fruits & Veggies—More Matters public health initiative. Journal of the American Dietetic Association, 111(10), 1570-1577.

46. Ruggieri, D. G., Bass, S. B., Alhajji, M., & Gordon, T. F. (2018). Understanding parents' perceptions of school-based BMI screening and BMI report cards using perceptual mapping: Implications for school nurses. *The Journal of School Nursing*, 1059840518789243.

47. Van Voorhees, B. W., Watson, N., Bridges, J. F., Fogel, J., Galas, J., Kramer, C., ... & Landsback, J. (2010). Development and pilot study of a marketing strategy for primary care/Internet–based depression prevention intervention for adolescents (The

For peer review only - http://bmjopen.bmj.com/site/about/guidelines.xhtml

CATCH-IT Intervention). Primary care companion to the Journal of clinical psychiatry, 12(3).

48. Sedlander, E., Rimal, R. N., Talegawkar, S. A., Yilma, H., & Munar, W. (2018). The RANI Project: A socio-normative intervention to reduce anemia in Odisha, India: A formative research protocol. *Gates open research*, 2.

49. Nguyen, P. H., Sanghvi, T., Kim, S. S., Tran, L. M., Afsana, K., Mahmud, Z., et al. (2017). Factors influencing maternal nutrition practices in a large scale maternal, newborn and child health program in Bangladesh. *PLoS One, 12*(7), e0179873.

50. Tinago, C. B., Annang Ingram, L., Blake, C. E., & Frongillo, E. A. (2017). Individual and structural environmental influences on utilization of iron and folic acid supplementation among pregnant women in Harare, Zimbabwe. *Maternal & child nutrition*, 13(3), e12350.

51. Bijmolt, T. H., & Wedel, M. (1999). A comparison of multidimensional scaling methods for perceptual mapping. *Journal of Marketing Research, 36*(2), 277-285.

52. Guest, G., MacQueen, K. M., & Namey, E. E. (2011). *Applied thematic analysis*. Sage Publications.

53. Das, A., & Sarkar, M. (2014). Pregnancy-related health information-seeking behaviors among rural pregnant women in india: Validating the wilson model in the indian context. *The Yale Journal of Biology and Medicine, 87*(3), 251.

54. Ramachandran, D., Canny, J., Das, P. D., & Cutrell, E. (2010). Mobile-izing health workers in rural india. Paper presented at the *Proceedings of the SIGCHI Conference on Human Factors in Computing Systems,* pp. 1889-1898.

For peer review only - http://bmjopen.bmj.com/site/about/guidelines.xhtml

55. Allendorf, K. (2013). Going nuclear? family structure and young women's health in india, 1992–2006. *Demography, 50*(3), 853-880.

56. Government of India. Census of India: 2011. New Delhi: Office of the Registrar General & Census Commissioner; 2012.

For peer review only

For peer review only - http://bmjopen.bmj.com/site/about/guidelines.xhtml

Table 1. Demographic Information- Perceptual Mapping and Card Sorting

	**Men** (n=30)	**WRA** (n=30)	**MILs** (n=30)
	M (SD)	***M (SD)***	***M (SD)***
**Age**	34.67 (6.00)	27.90 (4.76)	55.87 (7.58)
	**n (%)**	**n (%)**	**n (%)**
**School**			
None	3 (10)	4 (13.3)	21 (70.0)
Up to Primary	12 (40)	8 (26.7)	6 (20.0)
Up to Secondary	6 (20)	7 (23.3)	2 (6.7)
Up to High Secondary	5 (16.6)	10 (33.4)	1 (3.3)
Up to Tertiary	4 (13.3)	1 (3.3)	0 (0)
**Married**	30 (100)	30 (100)	29 (96.7)[a]
**Religion-Hindu**	100 (100)	100 (100)	100 (100)
**Caste**			
Scheduled Caste/ Tribe	18 (60.0)	16 (53.3)	17 (56.6)
Other Backwards Caste	11 (36.7)	12 (40.0)	13 (43.3)
Other Caste	1 (3.3)	2 (6.7)	0 (0)
**Children**			
None	0 (0)	3 (10)	0 (0)
1	4 (13.3)	7 (23.3)	3 (10.0)
2	12 (40.0)	10 (33.3)	3 (10.0)

For peer review only - http://bmjopen.bmj.com/site/about/guidelines.xhtml

FATIGUE AS A CUE TO SEEK TREATMENT

	Men	WRA	MILs
3	10 (33.3)	7 (23.3)	11 (36.7)
4 or more	4 (13.3)	3 (10.0)	13 (43.3)
**Ever taken IFA**	4 (13.3)	26 (86.7)	7 (23.3)
**Currently taking IFA**	0 (0)	3 (10.0)	2 (9.1)
**Diagnoses of anemia ever**	1 (3.3)	16 (53.3)	4 (13.3)
**Currently anemic?**	5 (21.7)	4 (14.3)	3 (13.0)

Note: Under “School”, “I” indicates incomplete and “C” indicates complete; [a] The one mother-in-law who wasn’t married was a widow. WRA = women of reproductive age; MILs = Mothers-in-law; IFA = iron folic acid.

$*p < 0.05$, $**p < 0.01$, $***p < 0.001$

For peer review only

Table 2. Importance to Health and Dislike Rating Across Participant Category

		WRA	Men	MILs	
Item	Rating Parameter	*M* (SD)	*M* (SD)	*M* (SD)	*F*
IFA pill	Importance	1.67 (0.68)	1.87 (0.35)	1.73 (0.52)	2.39
	Dislike	0.53 (0.73)	0.20 (0.48)	0.47 (0.73)	2.15
Clinic	Importance	1.80 (0.48)	1.93 (0.25)	1.83 (0.38)	0.98
	Dislike	0.23 (0.50)	0.20 (0.41)	0.23 (0.50)	0.05
Kiosk	Importance	1.77 (0.50)	1.93 (0.25)	1.87 (0.35)	1.44
	Dislike	0.27 (0.58)	0.17 (0.38)	0.13 (0.43)	0.64
Self-Help Groups	Importance	1.37 (0.72)	1.27 (0.74)	1.57 (0.68)	1.38
	Dislike	0.30 (0.47)[a]	0.73 (0.74)[b]	0.50 (0.68)[a,b]	3.44*
Traditional healer	Importance	0.80 (0.76)	1.23 (0.82)	1.03 (0.67)	2.50
	Dislike	1.07 (0.78)	0.97 (0.85)	1.07 (0.69)	0.17
Physician	Importance	1.80 (0.55)	2.00 (0)	1.90 (0.31)	2.27
	Dislike	0.20 (0.41)	0.10 (0.31)	0.20 (0.48)	0.61
Fatigue	Importance	0.77 (0.73)[a]	0.80 (0.61)[a]	0.17 (0.53)[b]	9.66***
	Dislike	1.47 (0.68)[a]	1.23 (0.73)[a]	1.87 (0.35)[b]	8.29***
Prenatal care	Importance	1.43 (0.73)	1.40 (0.67)	1.67 (0.61)	1.40
	Dislike	0.70 (0.89)	0.57 (0.50)	0.33 (0.66)	2.12
Green veggies	Importance	1.63 (0.62)	1.63 (0.49)	1.80 (0.41)	1.06
	Dislike	0.37 (0.61)	0.30 (0.60)	0.13 (0.35)	1.52
Meat	Importance	1.23 (0.68)	0.97 (0.76)	1.37 (0.61)	2.62
	Dislike	0.77 (0.73)	0.90 (0.76)	0.60 (0.62)	1.36

For peer review only

For peer review only - http://bmjopen.bmj.com/site/about/guidelines.xhtml

Pregnancy	Importance	1.13 (0.82)	1.23 (0.63)	1.57 (0.68)	3.04
	Dislike	0.80 (0.85)[a]	0.80 (0.48)[a]	0.30 (0.60)[b]	5.74**
Stomach-ache	Importance	0.63 (0.67)[a]	0.87 (0.68)[a]	0.30 (0.60)[b]	5.76**
	Dislike	1.47 (0.68)[a]	1.17 (0.65)[b]	1.83 (0.46)[c]	9.15***

Note: The letters a and b denote homogenous subsets yielded from the post-hoc analysis when omnibus significance was detected. WRA = women of reproductive age; MILs = Mothers-in-law; IFA = iron folic acid.

*p < 0.05, **p < 0.01, ***p < 0.001

For peer review only

Figure Captions.

Figure 1. Perceptual Map derived for Women of Reproductive Age. Dissimilarity plot across two-dimensions (corresponding to the x and y axis) as displayed by SPSS v. 25; plot point labels have been edited for reader clarity.

Figure 2. Perceptual Map derived for Men. Dissimilarity plot across two-dimensions (corresponding to the x and y axis) as displayed by SPSS v. 25; plot point labels have been edited for reader clarity.

Figure 3. Perceptual Map derived for Mothers-in-Law. Dissimilarity plot across two-dimensions (corresponding to the x and y axis) as displayed by SPSS v. 25; plot point labels have been edited for reader clarity.

For peer review only

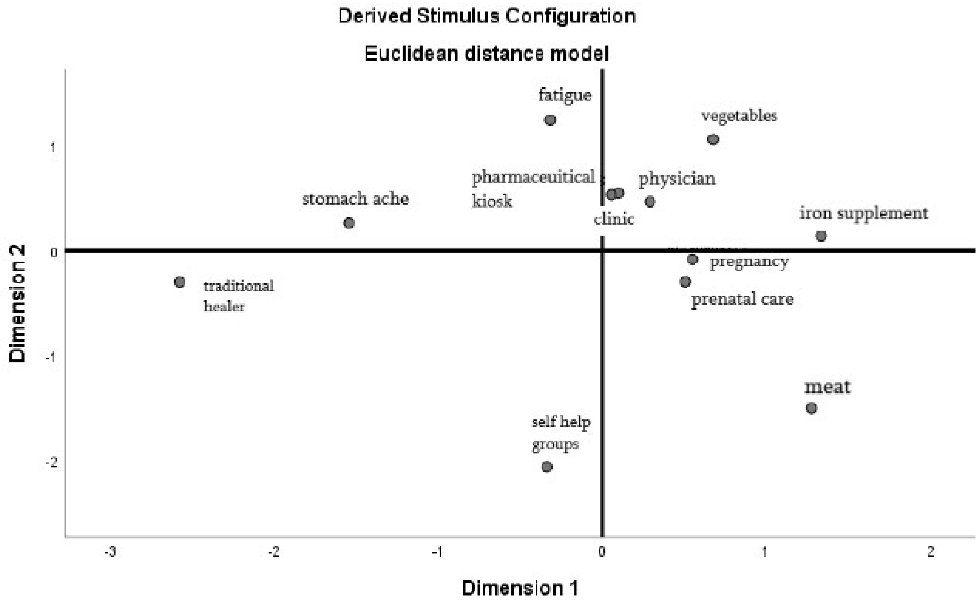

Figure 1. Perceptual Map derived for Women of Reproductive Age. Dissimilarity plot across two-dimensions (corresponding to the x and y axis) as displayed by SPSS v. 25; plot point labels have been edited for reader clarity.

171x107mm (96 x 96 DPI)

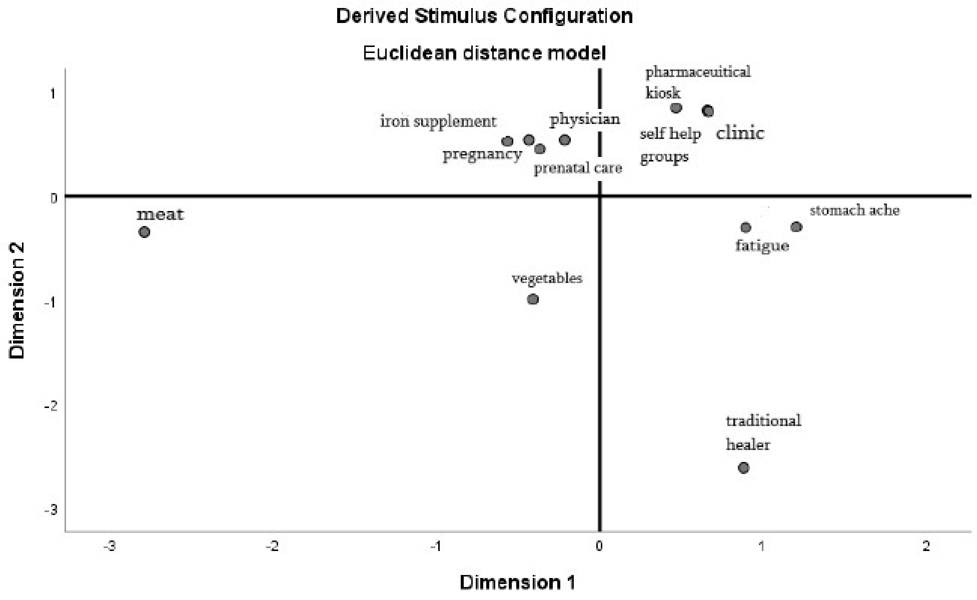

Figure 2. Perceptual Map derived for Males. Dissimilarity plot across two-dimensions (corresponding to the x and y axis) as displayed by SPSS v. 25; plot point labels have been edited for reader clarity.

175x109mm (96 x 96 DPI)

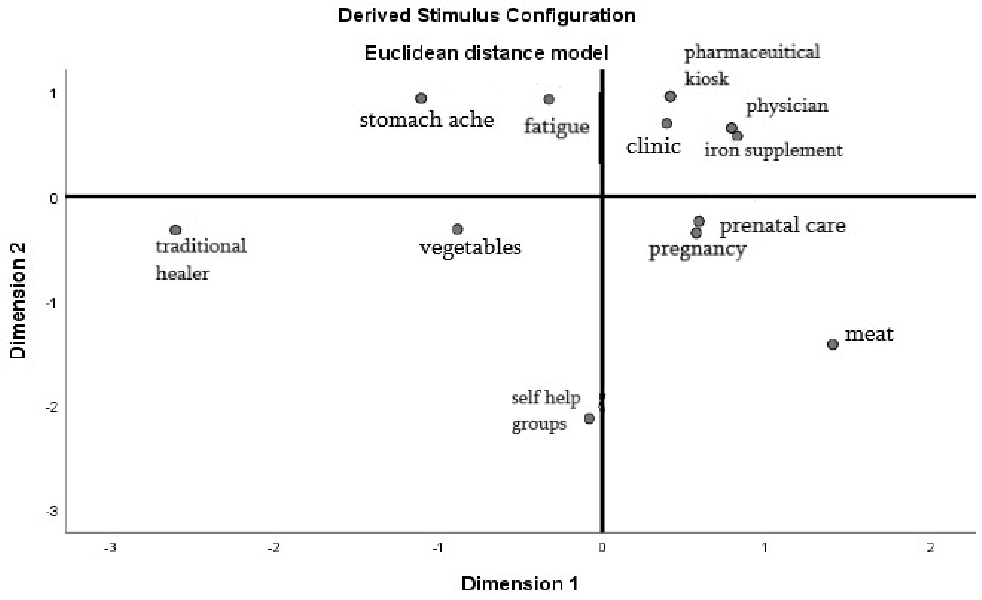

Figure 2. Perceptual Map derived for Males. Dissimilarity plot across two-dimensions (corresponding to the x and y axis) as displayed by SPSS v. 25; plot point labels have been edited for reader clarity.
Figure 3. Perceptual Map derived for Mothers-in-Law. Dissimilarity plot across two-dimensions (corresponding to the x and y axis) as displayed by SPSS v. 25; plot point labels have been edited for reader clarity.

177x111mm (96 x 96 DPI)

Supplement 1

PERCEPTUAL MAPPING AND CARD SORTING QUESTIONNAIRE

FOR RESEARCH TEAM MEMBER USE ONLY/ କେବଳ ଅନୁସନ୍ଧାନକାରୀ ଦଳର ସଦସ୍ୟଙ୍କ ବ୍ୟବହାର ନିମନ୍ତେ:		
BLOCK	Which Block is the respondent from/ଉତ୍ତରଦାତା କେଉଁ ବ୍ଲକରୁ?	....
VILLAGE	Which village is the respondent from/ଉତ୍ତରଦାତା କେଉଁ ଗ୍ରାମରୁ?	
PARTICIPATION	The respondent is participating in a...<br>ଉତ୍ତରଦାତାଜଣକ କେଉଁଥିରେ ଭାଗ ନେଉଛନ୍ତି	Focus group discussion.......................1<br>ଦଳଗତ ଆଲୋଚନା...1<br>Key informant interview........................2<br>ପ୍ରମୁଖ ସୂଚନାଦାତାଙ୍କ ସହିତ ସାକ୍ଷାତକାର ....2<br>Perceptual Mapping -------------------3<br>ଧାରଣାତ୍ମକ ମାନଚିତ୍ରଣ --------------------------3
CODE_PM	What is the code number of the Perceptual Mapping?<br>ଏହି ଧାରଣାତ୍ମକ ମାନଚିତ୍ରଣର କୋଡ କଣ?	Code number/ କୋଡ ନମ୍ବର୍ __________<br>PM1<br>PM2<br>PM3<br>PM4<br>PM5<br>PM6<br>PM7<br>PM8<br>PM9

For peer review only - http://bmjopen.bmj.com/site/about/guidelines.xhtml

		PM10<br>PM11<br>PM12
PARTICIPANT_PM	Participant category of Perceptual Mapping ଧାରଣାତ୍ମକ ମାନଚିତ୍ରଣରେ ଅଂଶଗ୍ରହଣ କରିଥିବା ଉତ୍ତରଦାତାଙ୍କ ପ୍ରକାର	Males 18 – 42 years old --------------------1<br>ପୁରୁଷ (୧୮-୪୨) (ବିବାହିତ) ------------------------1<br>WRA 15 – 35 years old ---------------------2<br>ପ୍ରଜନନ ବୟସ (୧୫-୩୫) ମଧ୍ୟରେ ଥିବା ମହିଳା ------2<br>Mothers in law – any age -----------------3<br>ଶାଶୁ (ସ୍ୱାମୀଙ୍କ ମା) ---------------------------------3
CODE_NUMBER_PM	Participant code number of the Perceptual Mapping ଧାରଣାତ୍ମକ ମାନଚିତ୍ରଣରେ ଭାଗ ନେଇଥିବା ସଦସ୍ୟଙ୍କ କୋଡ଼	
PATRICIPATION_DATE	On what date did the respondent participate/କେଉଁ ତାରିଖରେ ଉତ୍ତରଦାତା ଅଂଶଗ୍ରହଣ କରିଥିଲେ?	DD/MM: ________________<br>ତାରିଖ / ମାସ ________________

Thank you for participating in this study. I want to remind you that there are no right or wrong answers to these questions. We just want to know what your opinions are. Before we ask for your opinions, please tell us a few things about yourself/ଆପଣ ଏହି ଅଧ୍ୟୟନରେ ଅଂଶଗ୍ରହଣ କରିଥିବାରୁ ଧନ୍ୟବାଦ । ମୁଁ ଆପଣଙ୍କୁ ମନେ ପକେଇଦେବାକୁ ଚାହେଁ ଯେ ଏହି ସବୁ ପ୍ରଶ୍ନର ଠିକ କିମ୍ବା ଭୁଲ ଉତ୍ତର ନାହିଁ । ଆମେ କେବଳ ଆପଣଙ୍କ ମତାମତ ଗୁଡିକ ଜାଣିବାକୁ ଚାହୁଁଛୁ, ଦୟାକରି ଆପଣଙ୍କ ବିଷୟରେ ଆମକୁ କିଛି କୁହନ୍ତୁ ।

Section/ବିଭାଗ 1

Demographic Questionnaire/ଜନସଂଖ୍ୟା ସମ୍ବନ୍ଧୀୟ ପ୍ରଶ୍ନାବଳୀ

1	How old are you?<br>ଆପଣଙ୍କର ବୟସ କେତେ?	Age: ________________ (Unknown = -99)<br>ବୟସ: ------------------------ (ଜାଣି ନାହାନ୍ତି = -୯୯)
2	What is your sex?	Female/ମହିଳା 1

For peer review only - http://bmjopen.bmj.com/site/about/guidelines.xhtml

For peer review only

	ଆପଣଙ୍କର ଲିଙ୍ଗ କଣ?	Male/ପୁରୁଷ....................................2<br>No response/କୌଣସି ଉତ୍ତର ନାହିଁ..._......3
3	What is the highest level of school you have attended?<br>ଆପଣଙ୍କର ସର୍ବୋଚ୍ଚ ଶିକ୍ଷାଗତ ଯୋଗ୍ୟତା କଣ?	None/କିଛିନାହିଁ - 0<br>Primary school (incomplete)/ପ୍ରାଥମିକ ବିଦ୍ୟାଳୟ (ଅସମ୍ପୂର୍ଣ) - 1<br>Primary school (complete) /ପ୍ରାଥମିକ ବିଦ୍ୟାଳୟ (ସମ୍ପୂର୍ଣ) - 2<br>Secondary school (incomplete) /ମାଧ୍ୟମିକ ବିଦ୍ୟାଳୟ (ଅସମ୍ପୂର୍ଣ) - 3<br>Secondary school (complete) /ମାଧ୍ୟମିକ ବିଦ୍ୟାଳୟ (ସମ୍ପୂର୍ଣ) - 4<br>High Secondary/ Senior Secondary school (incomplete) /ଉଚ୍ଚ ମାଧ୍ୟମିକ ବିଦ୍ୟାଳୟ (ଅସମ୍ପୂର୍ଣ) - 5<br>High Secondary/ Senior school (complete) /ଉଚ୍ଚ ମାଧ୍ୟମିକ ବିଦ୍ୟାଳୟ (ସମ୍ପୂର୍ଣ) - 6<br>Tertiary/College/University (incomplete)/ମହାବିଦ୍ୟାଳୟ/ବିଶ୍ୱବିଦ୍ୟାଳୟ (ଅସମ୍ପୂର୍ଣ) - 7<br>Tertiary/College/University (complete)/ମହାବିଦ୍ୟାଳୟ/ବିଶ୍ୱବିଦ୍ୟାଳୟ (ସମ୍ପୂର୍ଣ) - 8<br>Other (Specify)/ଅନ୍ୟାନ୍ୟ (ଦର୍ଶାନ୍ତୁ):- 88<br>No response/କୌଣସି ଉତ୍ତରନାହିଁ...........-99
4	Are you currently married or living together with a partner as if married?<br>ଆପଣ ବିବାହିତ କି ବା ଜଣେ ସାଥିଙ୍କ ସହିତ ବିବାହିତ ଭଳି ମିଶିକରି ରୁହନ୍ତି କି ?	Yes, currently married/ହଁ, ବିବାହିତ...........1<br>Yes, living with a partner/ହଁ, ଜଣେ ସାଥୀଙ୍କ ସହିତ ରହୁଛି ....2<br>No/ନାହିଁ...3<br>No response/ କୌଣସି ଉତ୍ତର ନାହିଁ...........-99
4a	IF NO: Are you divorced, separated, widowed, or never married or in a union?<br>(Skip if married or living with a partner.)	Never married or in a union/କେବେ ବିବାହ କରିନାହାନ୍ତି କିମ୍ବା କୌଣସି ସାଥୀଙ୍କ ସହିତ ରଖୁନାହାଁନ୍ତ...1<br>Divorced/separated/ଛାଡପତ୍ର/ଅଲଗା ରହୁଛନ୍ତି...2<br>Widow/widower / ବିଧବା / ବିପତି.............3

For peer review only - http://bmjopen.bmj.com/site/about/guidelines.xhtml

	ଯଦି ନା : ଆପଣ କଣ ଛାଡପତ୍ର ନେଇଛନ୍ତି, ଅଲଗା ରହୁଛନ୍ତି, ବିଧବା ବା କେବେ ବିବାହ କରିନାହାନ୍ତି କିମ୍ବା କୌଣସି ସାଥୀଙ୍କ ସହିତ ନାହାନ୍ତି ?<br>(ପଚାରନ୍ତୁ ନାହିଁ, ଯଦି ବିବାହିତ କିମ୍ବା ସାଥି ସହିତ ରହୁଛନ୍ତି)	No response/ କୌଣସି ଉତ୍ତର ନାହିଁ.............-99
5	What is your religion?<br>ଆପଣଙ୍କର ଧର୍ମ କଣ ?	Hindu/ହିନ୍ଦୁ ....1<br>Muslim/ମୁସଲିମ ....2<br>Christian/ଖ୍ରୀଷ୍ଟିୟାନ..............................3<br>Sikh/ଶିଖ.......................................4<br>Buddhist/ବୁଦ୍ଧ...................................5<br>Jain/ଜୈନ..6<br>Other/ଅନ୍ୟାନ୍ୟ..................................7<br>No response/ କୌଣସି ଉତ୍ତର ନାହିଁ.........-99
CASTE	What is your caste ?<br>ଆପଣଙ୍କର ଜାତି କଣ ?	Scheduled Caste ଅନୁସୂଚିତ ଜାତି - 1<br>Scheduled Tribe ଅନୁସୂଚିତ ଜନଜାତି - 2<br>OBC ଅନ୍ୟାନ୍ୟ ପଛୁଆ ବର୍ଗର ଜାତି - 3<br>Other Caste ଅନ୍ୟାନ୍ୟ ଜାତି - 4<br>No response/ କୌଣସି ଉତ୍ତର ନାହିଁ.........-99
6	How many children do you have?<br>ଆପଣଙ୍କର କେତୋଟି ସନ୍ତାନ ଅଛନ୍ତି ?	None/କିଛିନାହିଁ....................................0<br>One/ଗୋଟିଏ.....................................1<br>Two/ଦୁଇଟି...2<br>Three/ତିନୋଟି....................................3<br>Four/ଚାରିଟି.....................................4<br>Five or more/ପାଞ୍ଚ କିମ୍ବା ଅଧିକ................5<br>No response// କୌଣସି ଉତ୍ତର ନାହିଁ...........-99
7	How often do you watch television?<br>ଆପଣ କେତେ ବ୍ୟବଧାନରେ ଟିଭି ଦେଖନ୍ତି ?	Not at all/କେବେ ନାହିଁ...............................0<br>Less than once a week/ସପ୍ତାହରେ ଥରକରୁ କମ୍...1<br>At least once a week/ ଅତିକମ୍ ରେ ସପ୍ତାହକୁ ଥରେ 2

For peer review only - http://bmjopen.bmj.com/site/about/guidelines.xhtml

		Almost Every Day/ପ୍ରାୟ ପ୍ରତ୍ୟକ ଦିନ............3
7a	On an average day, for how long do you watch television?<br>ଗୋଟିଏ ଦିନରେ, ପାଖାପାଖି କେତେ ସମୟ ଟିଭି ଦେଖନ୍ତି ?	______Hours per day/ OR ____ minutes per day<br>ଦିନରେ .... ଘଣ୍ଟା କିମ୍ବା ଦିନରେ .... ମିନିଟ୍
8	How often do you listen to the radio?<br>ଆପଣ କେତେଥର ରେଡିଓ ଶୁଣନ୍ତି ?	Not at all/କେବେ ନାହିଁ...............................0<br>Less than once a week/ ସପ୍ତାହରେ ଥରକରୁ କମ୍...1<br>At least once a week/ ଅତିକମ୍ ରେ ସପ୍ତାହକୁ ଥରେ ....2<br>Almost Every Day/ ପ୍ରାୟ ସବୁଦିନ.................3
8a	On an average day, for how long do you listen to the radio?<br>ଗୋଟିଏ ଦିନରେ ପାଖାପାଖି କେତେ ସମୟ ରେଡିଓ ଶୁଣନ୍ତି?	______Hours per day OR ____ minutes per day<br>ଦିନରେ .... ଘଣ୍ଟା କିମ୍ବା ଦିନରେ .... ମିନିଟ
9.0	How often do you talk on a mobile phone?<br>ଆପଣ କେତେଥର ମୋବାଇଲରେ କଥା ହୁଅନ୍ତି?	Not at all/କେବେ ନାହିଁ...............................0<br>Less than once a week/ ସପ୍ତାହରେ ଥରକରୁ କମ୍...1<br>At least once a week/ ଅତିକମ୍ ରେ ସପ୍ତାହକୁ ଥରେ ....2<br>Almost Every Day/ପ୍ରାୟ ସବୁଦିନ.................3
9	Do you use a smart phone?<br>ଆପଣ ସ୍ମାର୍ଟ ଫୋନ୍ ବ୍ୟବହାର କରନ୍ତି କି ?	Yes/ହଁ...1<br>No/ନା...0
9a	On an average day, for how long do you talk on a mobile phone?<br>ଗୋଟିଏ ଦିନରେ, ପାଖାପାଖି କେତେ ସମୟ ମୋବାଇଲରେ କଥା ହୁଅନ୍ତି?	______Hours per day OR ____ minutes per day<br>ଦିନରେ .... ଘଣ୍ଟା କିମ୍ବା ଦିନରେ .... ମିନିଟ
10	How often do you read the newspaper?<br>ଆପଣ କେତେଥର ଖବରକାଗଜ ପଢନ୍ତି?	Not at all/କେବେ ନାହିଁ...............................0<br>Less than once a week/ ସପ୍ତାହରେ ଥରକରୁ କମ୍...1<br>At least once a week/ ଅତିକମ୍ ରେ ସପ୍ତାହକୁ ଥରେ ....2<br>Almost Every Day/ପ୍ରାୟ ସବୁଦିନ.................3

For peer review only - http://bmjopen.bmj.com/site/about/guidelines.xhtml
For peer review only

10a	On an average day, for how long do you read the newspaper?<br>ଗୋଟିଏ ଦିନରେ, ପାଖାପାଖି କେତେ ସମୟ ଖବରକାଗଜ ପଢନ୍ତି?	______Hours per day OR ____ minutes per day<br>ଦିନରେ .... ଘଣ୍ଟା କିମ୍ବା ଦିନରେ .... ମିନିଟ
11	Have you ever attended a self-help group meeting?<br>ଆପଣ କେବେ ସ୍ୱୟଂ ସହାୟକ ଦଳର ବୈଠକରେ ଯୋଗ ଦେଇଛନ୍ତି କି ?	Yes/ହଁ..1<br>No/ନା..0
11a	Are you currently a part of a self-help group/ଆପଣ ବର୍ତ୍ତମାନ ସ୍ୱୟଂ ସହାୟକ ଦଳର ଜଣେ /ସଦସ୍ୟ କି?	Yes/ହଁ..1<br>No/ନା..0
12	Have you ever taken iron & folic acid supplements (IFA)/ଆପଣ କେବେ ବି ଆଇରନ ଏବଂ ଫଲିକ ଏସିଡର ପରିପୂରକ ଖାଇଛନ୍ତି କି ?	Yes/ହଁ..1<br>No/ନା..0
12a	Are you currently taking iron & folic acid supplements (IFA)/ଆପଣ ବର୍ତ୍ତମାନ ଆଇରନ ଏବଂ ଫଲିକ ଏସିଡର ପରିପୂରକ ଖାଉଛନ୍ତି କି ?	Yes/ହଁ..1<br>No/ନା..0
13	Has a doctor or healthcare provider ever told you that you have anemia/ଆପଣଙ୍କର ରକ୍ତହୀନତା ଅଛି ବୋଲି ଆପଣଙ୍କୁ ଡାକ୍ତର କିମ୍ବା କୌଣସି ସ୍ୱାସ୍ଥ୍ୟ ପ୍ରଦାନକାରୀ କେବେ କହିଛନ୍ତି କି ?	Yes/ହଁ..1<br>No/ନା..0
13a	Do you currently have anemia/ବର୍ତ୍ତମାନ ଆପଣଙ୍କର ରକ୍ତହୀନତା ରହିଛି କି ?	Yes/ହଁ..1<br>No/ନା..0

For peer review only

For peer review only - http://bmjopen.bmj.com/site/about/guidelines.xhtml

Section/ବିଭାଗ 2

I am now going to show you two cards at a time. One card has the picture of one object and the second card has the picture of a second object. Please tell me how similar the second card is to the first. To make this a bit easier, I will show you the second card and first ask you whether it is similar or different. If you say “similar,” then I will ask you whether it is somewhat similar or very similar; if you say “different,” then I will ask you whether it is somewhat different or very different.

ଏବେ ମୁଁ ଆପଣଙ୍କୁ ଏକ ସମୟରେ ଦୁଇଟି କାର୍ଡ ଦେଖାଇବାକୁ ଯାଉଛି | ଗୋଟିଏ କାର୍ଡରେ ଗୋଟିଏ ବିଷୟରେ ଛବି ଏବଂ ଦ୍ୱିତୀୟ କାର୍ଡରେ ଦ୍ୱିତୀୟ ବିଷୟରେ ଛବି ରହିଛି । ଦୟାକରି ମୋତେ କୁହନ୍ତୁ ଦ୍ୱିତୀୟ କାର୍ଡଟି ପ୍ରଥମ କାର୍ଡ ସହିତ କେତେ ସମାନ ଅଛି । ଆମେ ଏହାକୁ ଆଉ ଟିକେ ସହଜ କରିବା ପାଇଁ ମୁଁ ଆପଣଙ୍କୁ ଦ୍ୱିତୀୟ କାର୍ଡଟି ଦେଖାଇବି ଏବଂ ପ୍ରଥମେ ପଚାରିବି ଏହା ସମାନ କିମ୍ବା ଅଲଗା । ଯଦି ଆପଣ ସମାନ କହନ୍ତି ତେବେ କିଛି ସମାନ ନା ସମ୍ପୂର୍ଣ ସମାନ ବୋଲି ମୁଁ ପଚାରିବି; ଯଦି ଆପଣ ଅଲଗା କହନ୍ତି ତେବେ କିଛି ଅଲଗା ନା ସମ୍ପୂର୍ଣ ଅଲଗା ବୋଲି ମୁଁ ପଚାରିବି ।

[Show respondent each card pair: 1 versus 2, 3, 4,..... 16; 2 versus 3, 4, 5, ... 16; etc.]
[ଉତ୍ତରଦାତାଙ୍କୁ ପ୍ରତ୍ୟେକ କାର୍ଡର ଯୋଡି ଦେଖାନ୍ତୁ: 1 ସହିତ 2, 3, 4 ... 16; 2 ସହିତ 3, 4, 5, ... 16 ଇତ୍ୟାଦି]

1. IFA Pill/ଆଇରନ ବଟିକା
2. Clinic/କ୍ଲିନିକ
3. Kiosk
4. Self-help group/ସ୍ୱୟଂ ସହାୟକ ଦଳ
5. Traditional healer/ ପାରମ୍ପରିକ / ଚିକିତ୍ସକ / ବୈଦ୍ୟ
6. Physician/ ଚିକିତ୍ସକ / ଡାକ୍ତର
7. Fatigue/କ୍ଲାନ୍ତି
8. Prenatal care/ ଗର୍ଭାବସ୍ଥା ସମୟରେ ଯତ୍ନ
9. Green vegetables/ସବୁଜ ପନିପରିବା
10. Meat/ମାଂସ
11. Pregnancy/ଗର୍ଭାବସ୍ଥା
12. Stomach ache/ପେଟ କାଟିବା

[For each pair, first ask: Is XXXX similar or different from YYYY/ପ୍ରତ୍ୟେକ ଯୋଡି ପାଇଁ ପ୍ରଥମେ ପଚାରନ୍ତୁ XXXX ସମାନ କିମ୍ବା YYYY ଠାରୁ ଅଲଗା ।

For peer review only - http://bmjopen.bmj.com/site/about/guidelines.xhtml
For peer review only

If Respondent says, “similar,” then ask: Would you say just a bit similar or very similar?

ଯଦି ଉତ୍ତରଦାତା ସମାନ କହନ୍ତି ତେବେ ପଚାରନ୍ତୁ ଆପଣ କିଛି ସମାନ କହୁଛନ୍ତି କିମ୍ବା ସମ୍ପୂର୍ଣ ସମାନ କହୁଛନ୍ତି?

If Respondent says, “different,” then ask: Would you say just a bit different or very different?

ଯଦି ଉତ୍ତରଦାତା ଅଲଗା କହନ୍ତି ତେବେ ପଚାରନ୍ତୁ ଆପଣ କିଛି ଅଲଗା କହୁଛନ୍ତି କିମ୍ବା ସମ୍ପୂର୍ଣ ଅଲଗା କହୁଛନ୍ତି?]

For peer review only

For peer review only - http://bmjopen.bmj.com/site/about/guidelines.xhtml

Card 1	Card 2	1 = very different/ସମ୍ପୂର୍ଣ ଅଲଗା<br>2 = somewhat differentକିଛି ଅଲଗା<br>3 = somewhat similarକିଛି ସମାନ<br>4 = very similarସମ୍ପୂର୍ଣ ସମାନ
1. IFA pill/ଆଇରନ ବଟିକା	2	
	3	
	4	
	5	
	6	
	7	
	8	
	9	
	10	
	11	
	12	
2. Clinic କ୍ଲିନିକ	3	
	4	
	5	
	6	
	7	
	8	
	9	
	10	
	11	
	12	

Card 1	Card 2	1 = very different/ ସମ୍ପୂର୍ଣ ଅଲଗା<br>2 = somewhat different କିଛି ଅଲଗା<br>3 = somewhat similar କିଛି ସମାନ<br>4 = very similar ସମ୍ପୂର୍ଣ ସମାନ
3. Kiosk	4	
	5	
	6	
	7	
	8	
	9	
	10	
	11	
	12	
4. SHG ସ୍ୱୟଂ ସହାୟକ ଦଳ	5	
	6	
	7	
	8	
	9	
	10	
	11	
	12	

Card 1	Card 2	1 = very different/ସମ୍ପୂର୍ଣ ଅଲଗା<br>2 = somewhat differentକିଛି ଅଲଗା<br>3 = somewhat similar କିଛି ସମାନ<br>4 = very similar ସମ୍ପୂର୍ଣ ସମାନ
5. Traditional healer ପାରମ୍ପାରିକ ଚିକିତ୍ସକ / ବୈଦ୍ୟ	6	
	7	
	8	
	9	
	10	
	11	
	12	
6. Physician ଡାକ୍ତର	7	
	8	
	9	
	10	
	11	
	12	
7. Fatigue କ୍ଲାନ୍ତି	8	
	9	
	10	
	11	
	12	

For peer review only

For peer review only - http://bmjopen.bmj.com/site/about/guidelines.xhtml

Card 1	Card 2	1 = very different/ ସମ୍ପୂର୍ଣ୍ଣ ଅଲଗା<br>2 = somewhat different କିଛି ଅଲଗା<br>3 = somewhat similar କିଛି ସମାନ<br>4 = very similar ସମ୍ପୂର୍ଣ୍ଣ ସମାନ
8. Prenatal care ଗର୍ଭାବସ୍ଥା ସମୟରେ ଯତ୍ନ	9	
	10	
	11	
	12	
9. Green vegetables ସବୁଜ ପନିପରିବା	10	
	11	
	12	
10. Meat ମାଂସ	11	
	12	
11. Pregnancy/ଗର୍ଭାବସ୍ଥା	12	

For peer review only

For peer review only - http://bmjopen.bmj.com/site/about/guidelines.xhtml

Section/ବିଭାଗ 3

Importance for Health and Well-Being/ସ୍ୱାସ୍ଥ୍ୟ ଏବଂ ଭଲ ରହିବା ପାଇଁ ମହତ୍ୱ

I will now show you the same 12 cards. Please put these cards in three different piles. In the first pile, put all the cards that you think are very important for your health and well-being. In the second pile, put all the cards that you think are just a little bit important for your health and well-being. In the third pile, put all the cards that you think are not important at all for your health and well-being. [Do the exercise and write response in the first table. Then shuffle the cards.]
ଏବେ ମୁଁ ଆପଣଙ୍କୁ ୧୨ଟି କାର୍ଡ ଦେଖାଇବି । ଏହି କାର୍ଡଗୁଡିକ ତିନୋଟି ଅଲଗା ଗଦାରେ ରଖନ୍ତୁ | ଆପଣ ଯେଉଁ ସବୁକାର୍ଡ ଗୁଡିକ ଭାବୁଛନ୍ତି ଆପଣଙ୍କର ସ୍ୱାସ୍ଥ୍ୟ ଏବଂ ଭଲରହିବା ପାଇଁ ଜରୁରୀ ସେଗୁଡିକ ପ୍ରଥମ ଗଦାରେ ରଖନ୍ତୁ | ଯେଉଁସବୁ କାର୍ଡଗୁଡିକ ଆପଣଙ୍କ ସ୍ୱାସ୍ଥ୍ୟ ଏବଂ ଭଲ ରହିବା ପାଇଁ କମ ଜରୁରୀ ସେଗୁଡିକ ଦ୍ୱିତୀୟ ଗଦାରେ ରଖନ୍ତୁ | ସେହିପରି, ଯେଉଁସବୁ କାର୍ଡଗୁଡିକ ଆପଣଙ୍କ ସ୍ୱାସ୍ଥ୍ୟ ଏବଂ ଭଲରହିବା ପାଇଁ ଜମା ଜରୁରୀ ନୁହେଁ, ସେଗୁଡିକ ତୃତୀୟ ଗଦାରେ ଅଛି ? (ଏହି ଖେଳଟିକୁ କରାନ୍ତୁ ଏବଂ ଉତ୍ତରକୁ ପ୍ରଥମ ଟେବୁଲରେ ଲେଖନ୍ତୁ | ତାହାପରେ କାର୍ଡଗୁଡିକ ମିଶାଇଦିଅନ୍ତୁ |)

Easy/ସହଜ

This time please think about how easy it is to get (or to use or to do) each item shown in the card. If you think the item is very easy to get (or to use or to do), put it in the first pile. If you think the item is sometimes easy and sometimes difficult to get (or to use or to do), put it in the second pile. If you think it is very hard to get (or to use or to do), put it in the third pile. [Write response in the second table. Then shuffle.]
ଦୟାକରି ଏଥର ଚିନ୍ତା କରନ୍ତୁ କାର୍ଡରେ ଦେଖା ଯାଇଥିବା ପ୍ରତ୍ୟେକ ଜିନିଷକୁ କେତେ ସହଜରେ ପାଇ ପାରିବେ । ଯଦି ଭାବୁଛନ୍ତି ଏହି ଜିନିଷଗୁଡ଼ିକ ବହୁତ ସହଜରେ ପାଇପାରିବେ (ବା ବ୍ୟବହାରରେ ଲଗାଇପାରିବେ ବା କାମରେ ଲଗାଇପାରିବେ) ସେଗୁଡିକ ପ୍ରଥମ ଗଦାରେ ରଖନ୍ତୁ | ଯଦି ଭାବୁଛନ୍ତି ଏହି ଜିନିଷଗୁଡ଼ିକ ପାଇବାକୁ ବେଳେବେଳେ ସହଜ ଏବଂ ବେଳେବେଳେ କଷ୍ଟ (ବା ବ୍ୟବହାରରେ ଲଗାଇପାରିବେ ବା କାମରେ ଲଗାଇପାରିବେ), ତାହାକୁ ତୃତୀୟ ଗଦାରେ ରଖନ୍ତୁ | ଯଦି ଭାବୁଛନ୍ତି, ଏହା ପାଇବାକୁ (ବା ବ୍ୟବହାରରେ ଲଗାଇପାରିବେ ବା କାମରେ ଲଗାଇପାରିବେ) ବହୁତ କଷ୍ଟସାଧ ତାହାକୁ ତୃତୀୟ ଗଦାରେ ରଖନ୍ତୁ | (ଉତ୍ତରଗୁଡିକ ଦ୍ୱିତୀୟ ଟେବୁଲ ରେ ଲେଖନ୍ତୁ ଏବଂ ସେଗୁଡିକ ପରେ ମିଶାଇଦିଅନ୍ତୁ |)

Like/ପସନ୍ଦ

Finally, we will now sort these into three piles according to how much you like each card. If you like it a lot, put it in the first pile. If you like it just a bit or dislike it just a bit, put it in the second pile. And, if you dislike it a lot, put it in the third pile. [Record in the third table] ଶେଷରେ, ଆପଣ ପ୍ରତ୍ୟକ କାର୍ଡକୁ କେତେ ପସନ୍ଦ କଲେ, ସେହି ଅନୁସାରେ ଆଗେ ଏହାକୁ ତିନୋଟି ଗଦାରେ ସଜାଇ ରଖିବା | ଯଦି ଆପଣ ଏହାକୁ ବହୁତ ପସନ୍ଦ କରନ୍ତି, ତାହେଲେ

For peer review only - http://bmjopen.bmj.com/site/about/guidelines.xhtml

ତାକୁ ପ୍ରଥମ ଗଦାରେ ରଖନ୍ତୁ | ଯଦି ଆପଣ ଏହାକୁ କମ ପସନ୍ଦ କରନ୍ତି ବା କମ ପସନ୍ଦ କରନ୍ତି ନାହିଁ, ତାହାକୁ ଦ୍ୱିତୀୟ ଗଦାରେ ରଖନ୍ତୁ | ଯଦି ଆପଣ ସମ୍ପୂର୍ଣ୍ଣ ପସନ୍ଦ କରନ୍ତି ନାହିଁ, ତାହାକୁ ତୃତୀୟ ଗଦାରେ ରଖନ୍ତୁ) (ତୃତୀୟ ଟେବୁଲରେ ଲେଖନ୍ତୁ)

Write the card number in each column.

PILE 1: Very important ବହୁତ ଜରୁରୀ	PILE2: Somewhat important କିଛିମାତ୍ରାରେ ଜରୁରୀ	PILE 3: Unimportant ଜରୁରୀ ନୁହେଁ

PILE 1: Very easy to get ବହୁତ ସହଜରେ ପାଇ ହେବ	PILE 2: Somewhat easy କମ୍ ସହଜରେ ପାଇ ହେବ	PILE 3: Very hard to get ପାଇବାକୁ ବହୁତ କଷ୍ଟସାଧ୍ୟ

PILE 1: Like a lot ବହୁତ ପସନ୍ଦ କରନ୍ତି	PILE 2: Like somewhat କିଛି ପସନ୍ଦ କରନ୍ତି	PILE 3: Dislike ପସନ୍ଦ କରନ୍ତି ନାହିଁ

For peer review only

For peer review only - http://bmjopen.bmj.com/site/about/guidelines.xhtml

Supplement 2 – Focus Group Guide

Before Beginning the Interview:

- Welcome participant and introduce yourself

Hello, and thank you for speaking with me today. My name is ______ and I am a field researcher at DCOR. I am conducting a research study to understand the attitudes and beliefs of people in this community. Thank you so much for agreeing to participate and taking time out of your day.

Do you have any questions about the research and your participation before we start?

Warm-up Questions

I'm new to this community, [name District], can you tell me a little bit about it? For example, what kind of food do people eat here? And what do people do to earn money?

Women's Role in the Community

What are some of the typical things that women in this community do throughout the day? (probe: things like cooking, cleaning, taking care of kids, working outside the home, spending time with her husband).

Do women in this community have enough energy or time to these things each day?

- Look after her kids?
- Work outside the home?
- Cook?
- Spend time with her husband?
- Have more intimate time with her husband?

How are men and women treated differently in this community?
For women's life in general, are things getting better or worse?
- in her role as a mother?
- in her role as a wife?
- in her role as a daughter in law?

What are some of the concerns that women in this community have? (probe: concerns about health, money, their family, themselves, their future)

- Some people think that (woman's name) should first take care of her husband and kids before she worries about her own health. Other people think that she should first take care of her own health.

  How do you think most women in this community feel about that? What about most men?

  In some families in Odisha, women eat after their husband, children and mother in law eat. In others, women eat at the same time. What do you think about that? Is this changing?

For peer review only - http://bmjopen.bmj.com/site/about/guidelines.xhtml

Anemia and Iron Folic Acid (IFA) Knowledge

(Show an IFA tablet and liquid IFA)
Can someone tell me what this is? (probe: What does it do?)
Have you ever heard of anemia?
Many people in this community may not use the word "anemia" but they may have other words or phrases to talk about anemia. Can you tell me what some of those phrases are?
Tell me a little bit about what happens when someone has anemia (use the word or phrase identified above instead of "anemia"). Tell me how this person feels or how this person acts when they have anemia.
What do you think causes anemia? (probe: Not eating enough iron rich foods?)
What do you think makes anemia go away? (Probe: Can IFA tablets help? How about changing your diet? What kind of foods might help it go away?)

Anemia Related Behavior

Ok, now we're going to make up a character. She is a female aged 23 years old. What should we call her? (Ask for suggestions and decide on a name together). She just got married and is 3 months pregnant with her first child. Her doctor just told her she has anemia.

The doctor told her to take iron tablets.

- If (same woman's name) wanted to get IFA tablets, where could she get them? How difficult is it to *get* IFA tablets?
  - Can you tell me what she likes about taking the tablets and what she doesn't like? (PROBE by asking more likes and dislikes)
  - Apparently, some women stop taking those tablets. Can you tell me why they stop?
  - Can you tell me what good things may happen if someone takes the tablets?
  - What do you think would help her take the tablets? Do you think (woman's name)'s husband will support her to take the tablets? Not support her to take them? Or not say anything? (Ask for examples)
  - What about (woman's name)'s mother in law? Will she support her in taking the tablets? Why or why not?

IFA Norms

- Is Anemia is a problem in the community? If yes How much of a problem?
- In general, who is typically expected to take IFA tablets? (Probe: pregnant women, adolescent girls, non-pregnant women?)
- Please think about most women like (name) who live in this community. How many of them take IFA tablets? Some? Few? Most?
- Is there anyone in (woman's name) family (or her husband's family) that she can talk to about taking IFA tablets? How much do you think she'll listen to what they have to say about IFA tablets?

Information Sources

For peer review only

For peer review only - http://bmjopen.bmj.com/site/about/guidelines.xhtml

Imagine another woman who is not pregnant but interested in learning more about her health. What should we call her? (Ask for suggestions and decide on a name together).

Where does she go for information about health related matters?

Where can she get information about pregnancy, anemia, and IFA tablets from?

- How easy is it for her to get information about pregnancy, anemia, and/or IFA tablets? What difficulties would come up when trying to get this information?
- When a woman gets IFA tablet, what kind of information does she get about them?
- Probe: Dose, when to take or why to take it?
- For those of you that participate in self-help groups, can you tell me what kind of things you discuss?

Closing

That is the end of the questions I have for you, but do you have anything else you'd like to add to the discussion? Any little stories about anemia or IFA use in this community?

As a reminder, please do not share anything we spoke about today with anyone outside of this group.

Any questions?

Thank you for your time.

For peer review only

Is fatigue a cue to take iron supplements in Odisha India? A mixed methods investigation

SRQR Topic number	SRQR Topic	Page numbers (and line numbers if relevant)
Title and Abstract		
S1.	Title	p. 1; line 1
S2.	Abstract	p. 2
Introduction		
S3.	Problem formulation	p. 5 - 8
S4.	Purpose or research question	p. 9; lines 1-2 & 5-6
Methods		
S5.	Qualitative approach and research paradigm	p. 9 - 14
S6.	Research characteristics and reflexivity	p. 9 - 10
S7.	Context	p. 9 - 10
S8.	Sampling strategy	p. 10 – 11
S9.	Ethical issues pertaining to human subjects	p. 11; lines 13 - 23
S10.	Data collection methods	p. 12 - 13
S11.	Data collection instruments and technologies	bottom of page 12 to top of page 13
S12.	Units of study	p.14
S13.	Data processing	p.14
S14.	Data analysis	p.14
S15.	Techniques to enhance trustworthiness	p.9-10
Results/Findings		
S16.	Synthesis and interpretation	p. 15 - 18
S17.	Links to empirical data	p. 15 - 18
Discussion		
S18.	Integration with prior work, implications, transferability, and contributions to the field	p. 21 - 22
S19.	Limitations	p. 22
Other		
S20.	Conflicts of interest	p. 24
S21.	funding	p. 24

For peer review only
